# Sequence variants in *HECTD1* result in a variable neurodevelopmental disorder

**DOI:** 10.1016/j.ajhg.2025.01.001

**Published:** 2025-01-28

**Authors:** Gazelle Zerafati-Jahromi, Elias Oxman, Hieu D. Hoang, Wu-Lin Charng, Tanvitha Kotla, Weimin Yuan, Keito Ishibashi, Sonia Sebaoui, Kathryn Luedtke, Bryce Winrow, Rebecca D. Ganetzky, Anna Ruiz, Carmen Manso-Basúz, Nino Spataro, Peter Kannu, Taryn Athey, Christina Peroutka, Caitlin Barnes, Richard Sidlow, George Anadiotis, Kari Magnussen, Irene Valenzuela, Alejandro Moles-Fernandez, Seth Berger, Christina L. Grant, Eric Vilain, Gudny A. Arnadottir, Patrick Sulem, Telma S. Sulem, Kari Stefansson, Shavonne Massey, Natalie Ginn, Annapurna Poduri, Alissa M. D’Gama, Rozalia Valentine, Sara K. Trowbridge, Chaya N. Murali, Rachel Franciskovich, Yen Tran, Bryn D. Webb, Kim M. Keppler-Noreuil, April L. Hall, Bobbi McGivern, Kristin G. Monaghan, Maria J. Guillen Sacoto, Dustin Baldridge, Gary A. Silverman, Sonika Dahiya, Tychele N. Turner, Tim Schedl, Joshua G. Corbin, Stephen C. Pak, Irene E. Zohn, Christina A. Gurnett

**Affiliations:** 1 Department of Neurology, Washington University in St. Louis, St. Louis, MO, USA; 2 Department of Pathology, Washington University in St. Louis, St. Louis, MO, USA; 3 Department of Genetics, Washington University in St. Louis, St. Louis, MO, USA; 4 Department of Pediatrics, Washington University in St. Louis, St. Louis, MO, USA; 5 Center for Genetic Medicine Research, Children’s National Hospital, Washington, DC, USA; 6 Center for Neuroscience Research, Children’s National Hospital, Washington, DC, USA; 7 Mitochondrial Medicine Frontier Program, Division of Human Genetics, Children’s Hospital of Philadelphia, Philadelphia, PA, USA; 8 Department of Pediatrics, University of Pennsylvania Perelman School of Medicine, Philadelphia, PA, USA; 9 Center for Computational Genomics Medicine, Children’s Hospital of Philadelphia, Philadelphia, PA, USA; 10 Center for Genomic Medicine, Parc Taulí Hospital University, Parc Taulí Institute of Research and Innovation (I3PT-CERCA), Autonomous University of Barcelona, Sabadell, Spain; 11 Department of Medical Genetics, Alberta Health Services, Edmonton, AB, Canada; 12 Department of Pediatrics, University of Virginia, Charlottesville, VA, USA; 13 Department of Medical Genetics and Metabolism, Valley Children’s Hospital, Madera, CA, USA; 14 Department of Genetics and Metabolism, Randall Children’s Hospital at Legacy Emanuel, Portland, OR, USA; 15 Department of Clinical and Molecular Genetics, University Hospital Vall d’Hebron and Medicine Genetics Group, Valle Hebron Research Institute, Barcelona, Spain; 16 Rare Disease Institute, Children’s National Hospital, Washington, DC, USA; 17 deCODE Genetics/Amgen Inc., Reykjavik, Iceland; 18 Division of Neurology, Children’s Hospital of Philadelphia, Philadelphia, PA, USA; 19 Department of Neurology, Boston Children’s Hospital, Boston, MA, USA; 20 Department of Neurology, Harvard Medical School, Boston, MA, USA; 21 Division of Newborn Medicine, Boston Children’s Hospital, Boston, MA, USA; 22 Epilepsy Genetics Program, Department of Neurology, Boston Children’s Hospital, Boston, MA, USA; 23 Department of Pediatrics, Harvard Medical School, Boston, MA, USA; 24 Department of Molecular and Human Genetics, Baylor College of Medicine, Houston, TX, USA; 25 Department of Neurology, Baylor College of Medicine, Houston, TX, USA; 26 Department of Pediatrics, University of Wisconsin School of Medicine and Public Health, Madison, WI, USA; 27 Institute for Clinical and Translational Science, University of California, Irvine, Irvine, CA, USA; 28 GeneDx, Gaithersburg, MD, USA

**Keywords:** HECTD1, ubiquitin-proteasome system, neurodevelopmental disorders, epilepsy, autism

## Abstract

Dysregulation of genes encoding the homologous to E6AP C-terminus (HECT) E3 ubiquitin ligases has been linked to cancer and structural birth defects. One member of this family, the HECT-domain-containing protein 1 (*HECTD1*), mediates developmental pathways, including cell signaling, gene expression, and embryogenesis. Through GeneMatcher, we identified 14 unrelated individuals with 15 different variants in *HECTD1* (10 missense, 3 frameshift, 1 nonsense, and 1 splicing variant) with neurodevelopmental disorders (NDDs), including autism, attention-deficit/hyperactivity disorder, and epilepsy. Of these 15 *HECTD1* variants, 10 occurred *de novo*, 3 had unknown inheritance, and 2 were compound heterozygous. While all individuals in this cohort displayed NDDs, no genotype-phenotype correlation was apparent. Conditional knockout of *Hectd1* in the neural lineage in mice resulted in microcephaly, severe hippocampal malformations, and complete agenesis of the corpus callosum, supporting a role for *Hectd1* in embryonic brain development. Functional studies of select variants in *C*. *elegans* revealed dominant effects, including either change-of-function or loss-of-function/haploinsufficient mechanisms, which may explain phenotypic heterogeneity. Significant enrichment of *de novo* variants in *HECTD1* was also shown in an independent cohort of 53,305 published trios with NDDs or congenital heart disease. Thus, our clinical and functional data support a critical requirement of *HECTD1* for human brain development.

## Teaser

Dysregulation of homologous to E6AP C-Terminus (HECT) E3 ubiquitin ligases is linked to cancer and structural birth defects. Zerafati-Jahromi et al. describe *HECTD1* variants in 14 individuals with neurodevelopmental disorders and enrichment in 53,305 published trios. Critical for brain development, *HECTD1* variants show both change-of-function and loss-of-function mechanisms.

## Introduction

Many neurodevelopmental disorders (NDDs) result from genetic differences in the ubiquitin-proteasome system (UPS).^1^ Ubiquitylation is critical for almost all fundamental cellular processes by targeting substrate proteins to the proteasome for degradation or to modify their function by altering intracellular localization, activity, or protein interactions.^2^ E3 ubiquitin ligases provide selectivity to the ubiquitin cascade by imparting substrate specificity and comprise three subgroups: RING (really interesting new gene), HECT (homologous to E6AP C terminus), and RBR (RING between RING). HECT domain ligases have a C-terminal HECT domain E3, which interacts with the E2 ubiquitin-conjugating enzyme and accepts and transfers ubiquitin to substrates.^3^ *UBE3A* (MIM: 601623), responsible for Angelman syndrome (MIM: 105830), was the first HECT domain E3 ubiquitin ligase associated with an NDD.^4^^,^^5^ Genes encoding ubiquitin ligases, deubiquitylating enzymes, and proteasome subunits now comprise a significant proportion of known NDD genes.^1^^,^^6^

*HECTD1* (MIM: 618649) encodes a large protein (2,610 amino acids) with a C-terminal HECT domain and multiple protein-protein interaction domains. The protein interaction domains include two armadillo repeat domains, ankyrin-repeat domain, SUN (SAD1/UNC) domain, MIB (MIB/HERC2) domain, and basic tilted helix bundle (BTHB) domain. Studies in mouse models with loss-of-function mutations in *Hectd1* demonstrate a requirement for this gene in neural tube, hematopoietic stem cell, heart, and placental development.^7^^,^^8^^,^^9^^,^^10^^,^^11^^,^^12^ Moreover, sequence variants in *HECTD1* have been reported in human neural tube defects (NTDs) and a single congenital heart defect (CHD) case, as well as in large autism and NDD cohorts.^13^^,^^14^^,^^15^^,^^16^ A machine-learning model that considered gene expression patterns in the brain, network features, and constraint scores demonstrated that *HECTD1* was among the top ten candidates for autism genes.^17^ However, the role of *HECTD1* in brain development and the etiology of human NDDs remains largely unknown.

Here, we describe a cohort of 14 individuals with 15 variants of uncertain significance in the *HECTD1* gene, including 10 missense, 3 frameshift, 1 nonsense, and 1 splicing variant associated with a spectrum of NDD phenotypes. These phenotypes, observed in 10 individuals with *de novo* variants, include autism, attention deficit/hyperactivity disorder (ADHD [MIM: 143465]), and epilepsy. A role for *HECTD1* variants in human neurobehavioral phenotypes is further supported by a significant enrichment of *HECTD1 de novo* variants in an independent cohort of 53,305 published trio sequencing cases involving probands diagnosed with either NDDs or congenital heart disease, brain abnormalities in *Hectd1* mutant mice, and variable effects of select missense variants in *C*. *elegans* models.

## Subjects and methods

### Subject recruitment and sequencing

The index individual was identified through a research sequencing study at Washington University in St. Louis and confirmed by clinical sequencing at GeneDx. The other 13 individuals were identified through GeneMatcher.^18^ The initial GeneMatcher entry occurred on November 28, 2016. This study was conducted with informed consent of research participants under the Washington University School of Medicine Institutional Review Board (IRB) or the IRB affiliated with each participating center. The clinician for each included individual obtained consent for publication under their own site’s protocols. Genome and exome-wide sequencing was performed in clinical diagnostic or research laboratories at each participating center using standard sequencing and analysis methods. The *de novo* inheritance of variants was confirmed with a trio design whenever parents were available for testing. Clinical data were gathered by means of retrospective chart review using a standardized survey completed by each clinician. Histological images of the resected cortex were of samples taken during epilepsy surgery in individual 2.

### *In silico* analysis

Sequence data were acquired from National Center for Biotechnology Information (NCBI), and references were from GenBank using the GRCh28p.13 primary assembly. NCBI Reference Sequence accession RefSeq: NM_015382.4 was used to define the cDNA and amino acid changes. These variants were analyzed *in silico* using multiple pathogenicity scores, including Combined Annotation-Dependent Depletion (CADD),^19^ REVEL,^20^ and PolyPhen2.^21^ Variants were also annotated with Genome Aggregation Database (gnomAD) minor allele frequencies.^22^

The predicted *HECTD1* three-dimensional (3D) structure (AF-Q9ULT8-F1) was obtained from the AlphaFold Protein Structure database.^23^^,^^24^ Annotation of the variant locations and motifs was performed using iCn3D (https://www.ncbi.nlm.nih.gov/Structure/icn3d/full.html). We acknowledge that AlphaFold may represent a 3D model unconfirmed for purposes of functional analysis; the figure shown depicts a basic representation of the spatial and secondary structure orientation of individual missense variants. Sequence alignment focused on the amino acid sequences around missense variants using a previously conducted blastp and MUSCLE (multiple sequence comparison by log-expectation) analysis.^14^ Multiple sequence alignment was visualized using SnapGene Software (from Dotmatics; available at Snapgene.com).

### Analysis of mouse models

All mouse work was performed in accordance with protocols approved by the Institutional Animal Care and Use Committee of the Children’s National Research Institute. The *Hectd1*^*opm*^ and *Sox1*^*tm1*(*cre*)*Take*^ (*Sox1cre*) lines were previously described.^7^^,^^25^ The *Hectd1*^*flox*^ line was generated by crossing the previously described *Hectd1*^*tm1a*(*EUCOMM*)*Hmgu*^ line to a FLPo deleter strain (*Gt*(*ROSA*)*26Sor*^*tm2*(*FLP∗*)*Sor*^; JAX stock #012930).^10^^,^^26^ All mice were maintained on a congenic 129/SvJ background following 5–10 generations of backcrossing. On postnatal days 27–37 (P27–P37), mice were weighed, anesthetized, and transcardially perfused with 4% paraformaldehyde in PBS. Brains were removed, weighed, post-fixed overnight, cryoprotected in 30% sucrose in PBS, embedded in OCT, cryosectioned at a thickness of 30 μm, mounted on microscope slides, and allowed to dry. Nissl staining with cresyl violet was followed by dehydration in an alcohol gradient, xylene clearance, and coverslipping of the slides. Brain weights were taken from eight cKO and eight wild-type mice across 11 litters. Of these, Nissl staining was done on seven cKO and three wild-type brains. Images were acquired with a Leica DM6B microscope at 10× and processed in ImageJ and Adobe Photoshop, and figures were assembled in Microsoft PowerPoint. Images of dissected brains were acquired with an iPhone 14. Statistical analysis of brain and body weights was performed in GraphPad Prism version 10.2.3, and significance was determined by Student’s t test. The chi-squared test was used to determine the significance of morphological phenotypes in Nissl-stained brains using an online calculator (http://physics.csbsju.edu/stats/).

### *C*. *elegans* strains and culture conditions

*C*. *elegans* were cultured on nematode growth media (NGM) plates seeded with *E*. *coli* strain OP50 as previously described.^27^ All experiments were performed using homozygous animals at 20°C except when indicated that heterozygotes were used. Heterozygotes were generated by crossing homozygous (control or variant) females with wild-type males. Females were generated by treating animals with *fem-1* RNAi to abolish spermatogenesis as previously described.^28^ VC2010 (wild-type parental strain) and deletion (Δ) allele (*ok1437*), which carries a 1,239 bp deletion in *hecd-1* gene and results in a truncated HECD-1 protein losing its C-terminal half from residue 1,153 to the end, were obtained from the *Caenorhabditis* Genetics Center (CGC; www.cgc.umn.edu). All strains were backcrossed a minimum of two times with the wild-type strain (VC2010) prior to phenotyping. A complete list of strains used in this study and their genotypes are shown in Table S1.

### *C*. *elegans* genome editing using CRISPR-Cas9

Cas9 protein, guide RNAs, and single-stranded 100-base DNA oligos were microinjected into gonads of young adult hermaphrodites to generate *hecd-1* human variant and control edit strains. Cas9 protein (S.p. HiFi Cas9 Nuclease V3), tracRNA, crRNAs, and DNA repair template oligos were purchased from IDT (www.idtdna.com). The sequences of crRNA specific to the target loci and the corresponding repair templates are listed in Table S2. Of the multiple isoforms of *hecd-1* reported on the WormBase website (http://www.wormbase.org), isoform d was used, as it is the most abundant.^29^ Independently isolated variant or control strains were labeled with # and a number (e.g., Arg350Gly #1 and Arg350Gly #2). All strains were outcrossed two times with the wild type (VC2010 strain) to reduce genetic background differences. The entire *hecd-1* gene was sequenced in all edited strains to ensure that no off-target changes were inadvertently introduced during editing.

### Measurement of *C*. *elegans* crawl speed, thrash rate, and body length

WormLab (MBF Bioscience) was used to acquire videos of worms (i.e., 24 h post L4 larval stage) crawling on 60-mm NGM plates seeded with *E*. *coli* and thrashing in PBS liquid as previously described.^28^^,^^30^ WormLab software (version 2019.1.1) was used to calculate crawl speed, head turn count, and body length. Thrash rate is defined as the number of head turn cycles per minute (where 1 turn cycle is equal to 2 turn counts).

### Quantification of *C*. *elegans* ubiquitin-GFP accumulation

Ubiquitin green fluorescent protein (GFP) marker strain (allele *odIs77*) was characterized by Liu et al.^31^ The strain expresses UB^G76V^-GFP (abbreviated as UbGFP) and mRFP (red fluorescent protein) under the control of the *col-19p* promoter, which drives expression in the hypoderm. HECD-1 polyubiquitylates the UbGFP and therefore marks it for degradation. mRFP was used to control for gene expression. UbGFP levels were quantified in 2-day-old adult animals (i.e., 48 h post L4 larval stage). Animals were homozygous or heterozygous for *hecd-1* (controls or variants) and homozygous for the UbGFP marker. UbGFP and mRFP were quantified using the CellInsight CX7 high-content imager (Thermo Scientific). Approximately ten worms were placed into a well of a 384-well plate containing PBS buffer + 0.05% pluronic acid, a surfactant to prevent worms from sticking to the wall of the well. The worms were then anesthetized with levamisole (1.5 mg/mL) in the same buffer for 5 min before image acquisition. GFP and RFP thresholds were set at 25% maximum intensity of wild-type worms expressing the *odIs77* transgene. Total intensities from GFP and RFP channels were measured, and UbGFP accumulation was expressed as a ratio of GFP/RFP signal intensities.

### Proteasomal inhibitor (bortezomib) treatment of *C*. *elegans* expressing Ub-GFP

Bortezomib eight-point dose-response studies were performed in 384-well plates. One-day-old adult animals were treated with 100 μM, 31.25 μM, 10 μM, 3.125 μM, 1 μM, 0.3125 μM, 0.1 μM, and 0.01 μM concentrations of bortezomib (Millipore Sigma, cat. #5.04314) or 0.2%, 0.0625%, 0.02%, 0.00625%, 0.002%, 0.000625%, 0.0002%, or 0.00002% DMSO (which corresponds to the DMSO concentration in the corresponding drug concentrations). Animals were exposed to the drug or DMSO for 18 h, anesthetized using levamisole, and imaged using the CellInsight CX-7 high-content imager as previously described.^32^ Levels of UbGFP and mRFP were quantified and expressed as a ratio of GFP/mRFP.

### Quantification of *C*. *elegans* unfolded protein response using HSP-4/GFP

The HSP-4/GFP reporter line (allele *zcIs4*) was used as a marker for endoplasmic reticulum (ER) stress and unfolded protein response (UPR) as previously described.^33^ HSP-4/GFP was driven under *hsp-4* promoter and was expressed at low intensity under normal growing conditions. HSP-4/GFP expression was measured in 3-day-old adult animals. GFP total intensity was measured on the CellInsight CX7 high-content imager as described above. Data were expressed as GFP total intensity per animal.

### Statistics and graphs

Data from at least three biological replicates from separate individual worms or worm populations were reported. Normality was determined by QQ plots, D’Agostino and Pearson tests, Anderson-Darling tests, Shapiro-Wilk tests, and Kolmogorov-Smirnov tests. Datasets with non-normal distribution were graphed as median + interquartile range. Kruskal-Wallis tests (non-parametric alternative of ANOVA) were used to determine statistical significance between different genotypes. For normally distributed datasets, equal variance was determined using Bartlett’s tests. Normally distributed datasets were graphed as mean + 95% confidence interval. Of those, ordinary one-way ANOVA was used to determine difference between genotypes in equal-variance datasets. Brown-Forsythe and Welch one-way ANOVA tests were used to determine statistical significance between genotypes in unequal-variance datasets. All statistical tests and graphs were generated using GraphPad Prism version 10.2.3.

## Results

### Genetics findings

Our index individual (individual 2) presented for evaluation at 8 years of age with new-onset focal epilepsy. Clinical trio exome sequencing reported a *de novo* missense variant of uncertain significance in the candidate gene *HECTD1*. Through responses to GeneMatcher, we identified 13 additional unrelated individuals with *HECTD1* variants (Table 1). The individuals’ current ages range from 6 months to 28 years old. There were eight males and six females. From these 14 individuals, we identified 15 *HECTD1* variants, including compound heterozygous frameshift and missense variants in individual 14, both inherited from apparently healthy parents. Of the other 13 variants, ten were *de novo* and three were of unknown inheritance.

**Table 1:** Proband *HECTD1* variants

**Individual ID**	**Sequence variant (c.DNA)**	**Amino acid change**	**Inheritance**	**gnomAD MAF**	**Other variants**
1	c.140C>T	p.Thr47Ile	unknown	0	–
2	c.710T>C	p.Leu237Ser	*de novo*	0	–
3	c.1111C>G	p.Arg371Gly	*de novo*	0	–
4	c.2082C>G	p.Phe694Leu	*de novo*	0	–
5	c.3346G>A	p.Asp1116Asn	*de novo*	0	*COL5A1* VUS
6	c.3350T>C	p.Ile1117Thr	*de novo*	0	–
7	c.3709T>A	p.Tyr1237Asn	*de novo*	0	–
8	c.3994C>T	p.Leu1332Phe	*de novo*	0	–
9	c.7135C>T	p.Leu2379Phe	*de novo*	0	*TTN* likely pathogenic
10	c.1333−6_1333−4del	Splice variant (in intron 8 from −4 to −6 of splice site)	*de novo*	0	–
11	c.2349dup	p.Asp784ArgfsTer8	unknown	0	*DLL1* likely pathogenic
12	c.3670G>T	p.Gly1224Ter	*de novo*	0	–
13	c.7433dup	p.Leu2478PhefsTer7	unknown	0	–
14	c.6536delC	p.Ala2179ValfsTer35	compound heterozygous	0	–
14	c.476A>G	p.His159Arg	compound heterozygous	0	–

RefSeq mRNA: NM_015382.4; RefSeq protein: NP_056197.3; VUS, variant of uncertain significance; MAF, minor allele frequency.

Five variants were predicted to result in a non-functional ubiquitin ligase due to truncation before the catalytic HECT domain. Among these were three frameshift variants (including one in individual 14 that occurred as a compound heterozygote with a missense variant), one nonsense, and one splicing variant (Table 1). None of these variants are in the last exon of the gene, and thus the variant-containing mRNAs are likely to undergo nonsense-mediated decay. Notably, as reported in the gnomAD database, *HECTD1* is highly intolerant to loss-of-function variation, with a loss-of-function-intolerant (pLI) score of 1 and a loss-of-function observed/expected upper bound fraction (LOEUF) score of 0.27, consistent with *HECTD1* being a likely haploinsufficient disease gene.^14^^,^^34^^,^^35^

Additional genetic findings that may be relevant to clinical phenotypes in this cohort include a paternally inherited variant of uncertain significance (VUS) in *COL5A1* (MIM: 120215) (Ehlers-Danlos syndrome, classic type [MIM: 130000]) in individual 5, which may explain his arachnodactyly, pectus carinatum, kyphosis, and aortic dilatation. A likely pathogenic frameshift variant in *DLL1* (MIM: 606582) of unknown inheritance was identified in individual 11, which was previously associated with neurodevelopmental phenotypes (MIM: 618709), including epilepsy and cortical malformations.^36^ Individual 3 had severe mitochondrial complex I deficiency and moderate mitochondrial complex IV deficiency identified in liver electron transport chain enzymology, with no known genetic etiology, which likely explains his persistent transaminase elevations. No other individuals had reportable genetic findings that could explain the observed NDDs.

### Human missense variants

**Figure 1:**
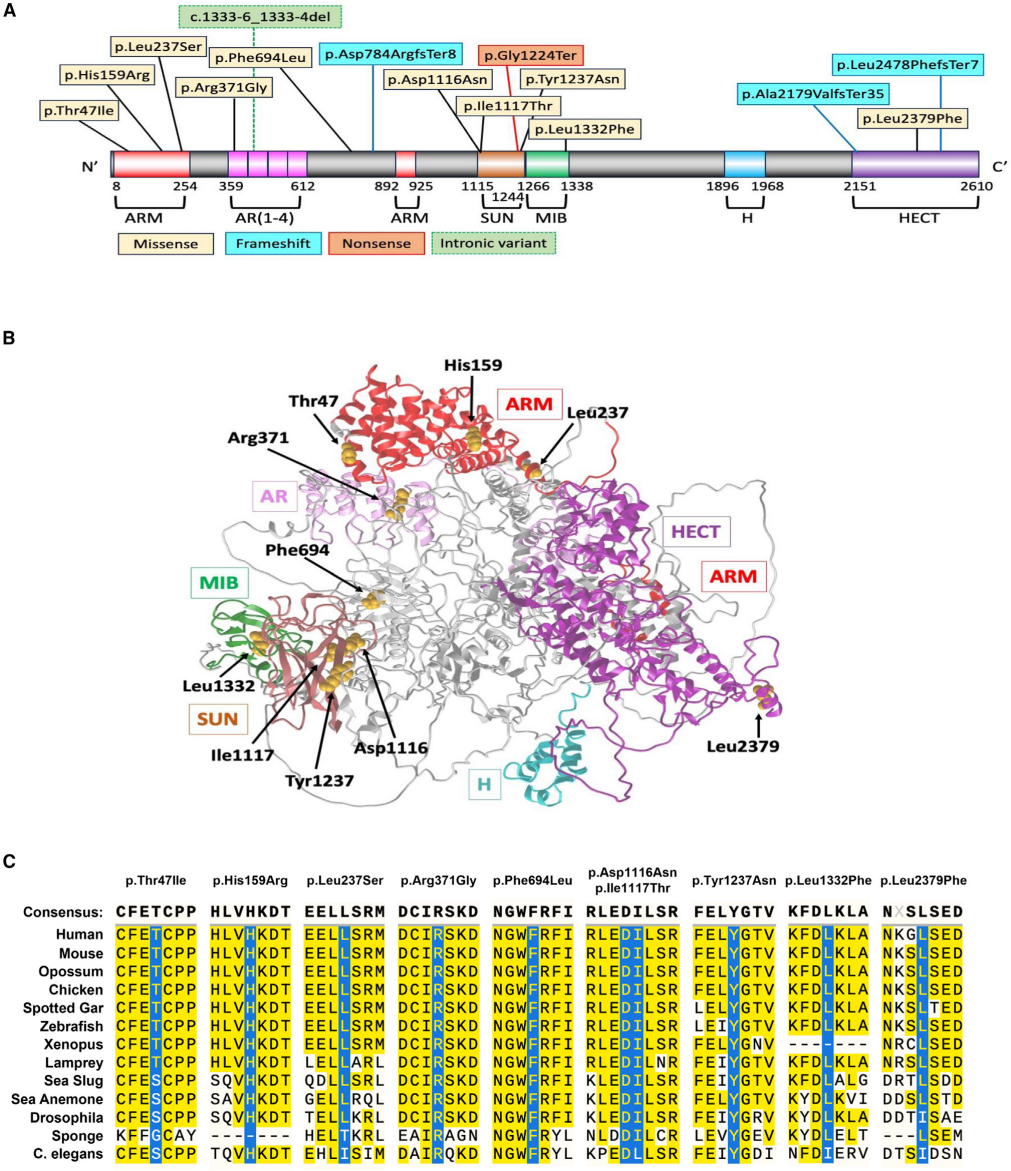
Location of *HECTD1* variants (A) Variant subtypes and their locations within protein domains. (B) Three-dimensional model showing location of variants near protein domains. (C) Multiple sequence alignment for missense variants obtained through taxonomic filters on blastp with MUSCLE. Variant p.His159Arg is a compound heterozygote with p.Ala2179Vfs^∗^35 in individual 14.

The *HECTD1* gene (RefSeq: NP_056197.3; RefSeq: NM_015382.4) is highly constrained for missense variation, with a missense *Z* score of 6.42.^34^^,^^35^ Ten missense variants occurred throughout multiple domains of the protein (Figure 1A) with no apparent three-dimensional clustering (Figure 1B). Only one missense variant (c.7135C>T [p.Leu2379Phe]) was identified in the HECT domain, which is essential for the ubiquitylation activity of HECTD1.^37^ The majority of missense variants localize to protein interaction domains within HECTD1. Three variants (c.140C>T [p.Thr47Ile], c.476A>G [p.His159Arg], and c.710T>C [p.Leu237Ser]) are localized within the N-terminal ARM (armadillo-like helical) domain. The c.1111C>G (p.Arg371Gly) and c.2082C>G (p.Phe694Leu) variants flank the ankyrin repeat domain. Two adjacent amino acids within the SUN (Sad1 and UNC-84) domain, which may play a role in the localization of HECTD1 within the cell,^38^ are altered by variants c.3346G>A (p.Asp1116Asn) and c.3350T>C (p.Ile1117Thr), with a third variant c.3709T>A (p.Tyr1237Asn) in close physical proximity. The final missense variant, c.3994C>T (p.Leu1332Phe), is localized within the MIB-HERC2 (mind bomb and HECT and RLD domain containing E3 ubiquitin protein ligase 2) domain, which is present in many ubiquitin ligases and may mediate interactions with substrates.^39^ The localization of missense variants within or near key protein interaction domains suggests they may disrupt interaction with specific HECTD1-interacting proteins and substrates.

Numerous *in silico* models were used to predict the impact of the *HECTD1* missense variants. Multiple sequence alignment demonstrated a high degree of conservation between numerous vertebrate and invertebrate species (Figure 1C), and missense variants were predicted to be damaging by most algorithms (Table S3). All missense variants altered amino acids that are conserved among vertebrates. Variants p.Arg371Gly, p.Phe694Leu, p.Asp1116Asn, and p.Tyr1237Asn changed amino acids that are conserved across all species examined (Figure 1C). Moreover, residues not conserved in invertebrates were similar and had identical flanking residues.

### Clinical phenotypes

Clinical descriptions for each of the 14 individuals are available in the supplemental case reports. Overall, abnormal growth parameter trends were not identified within this cohort of individuals with *HECTD1* variants, and no distinctive facial features were noted. Congenital anomalies were infrequent but diverse (Table 2). Oropharyngeal dysphagia, manifesting as a poor latch in infancy, aspiration, or failure to thrive, was common, resulting in gastric feeding tube placement in 5 of the 14 individuals (36%). Three of eight males had cryptorchidism (38%). Individual 9 had multiple congenital anomalies resulting in demise at age 6 months, including severe pulmonary vein stenosis, enlarged left kidney with cyst, duodenal atresia with tracheoesophageal fistula, biliary atresia with absent gallbladder, and imperforate anus. Individual 7 had tetralogy of Fallot and Pierre Robin sequence with facial clefting and developed postnatal microcephaly. Individual 4 had a laryngeal cleft. Individual 14, who had compound heterozygous variants consisting of both frameshift and missense variants that were inherited from each parent, had significant growth impairment affecting weight (−4.3 SD) and height (−5.5 SD), global developmental disabilities, and microcephaly. There were no consistent phenotypic differences between individuals with predicted loss-of-function variants and those with missense variants (Table 3).

**Table 2:** Clinical phenotypes of individuals with *HECTD1* variants

	**Individual**													
	**1**	**2**	**3**	**4**	**5**	**6**	**7**	**8**	**9**	**10**	**11**	**12**	**13**	**14**
	**Missense**									**Likely gene disruptive**				
Amino acid change	p.Thr47Ile	p.Leu237Ser	p.Arg371Gly	p.Phe694Leu	p.Asp1116Asn	p.Ile1117Thr	p.Tyr1237Asn	p.Leu1332Phe	p.Leu2379Phe	splice var.	p.Asp784ArgfsTer8	p.Gly1224Ter	p.Leu2478PhefsTer7	p.Ala2179ValfsTer35, p.His159Arg
Sex	F	M	M	F	M	M	M	F	M	M	F	F	M	F
Age	10 years	28 years	5 years	7 years	18 years	6 months	9 months	9 years	6 months	17 years	5 years	19 years	10 years	5 years

**Development**														

Language	+	+	−	+	+	−	+	−	N/A	+	+	+	+	+
Fine motor	−	+	−	+	+	−	+	+	N/A	N/A	−	+	+	+
Gross motor	−	−	−	+	−	−	+	−	N/A	N/A	−	+	−	+
Intellectual disability	N/A	+	N/A	N/A	+	N/A	N/A	N/A	N/A	N/A	N/A	+	N/A	N/A

**Neurological and psychiatric phenotypes**														

Microcephaly	−	−	−	−	−	−	+	−	−	−	+	−	−	+
Epilepsy	+	+	+	−	−	+	−	+	−	N/A	+	−	−	−
Abnormal muscle tone	−	−	−	+	−	+	+	+	−	N/A	−	+	+	−
ADD/ADHD	N/A	+	−	N/A	+	N/A	N/A	+	N/A	+	−	+	+	+
ASD	+	−	−	+	−	N/A	N/A	−	N/A	+	+	−	+	−
Anxiety	N/A	−	−	−	−	N/A	N/A	+	N/A	N/A	+	+	+	−
Self-injurious behavior	N/A	−	−	+	−	N/A	N/A	−	N/A	N/A	−	+	−	−
Hallucinations	N/A	+	−	−	−	N/A	N/A	−	N/A	N/A	−	−	+	−
Aggressive behavior	N/A	+	−	+	−	N/A	N/A	−	N/A	N/A	−	−	−	−
Abnormal brain MRI	+	+	−	+	−	−	−	−	+	N/A	+	−	N/A	−

**Systemic**														

Dysphagia	−	−	−	+	+	+	+	−	−	N/A	−	−	−	−
Tube feeding	−	−	+	+	−	−	+	+	+	N/A	−	−	−	−
Cryptorchidism	N/A	−	−	N/A	+	−	+	N/A	−	−	N/A	N/A	+	N/A
Congenital heart disease	−	−	−	−	−	−	+	−	+	−	−	−	−	−

RefSeq protein: NP_056197.3; N/A, data was not reported; ADD, attention deficit disorder; AHDH, attention deficit/hyperactivity disorder; ASD, autism spectrum disorder; MRI, magnetic resonance imaging.

**Table 3:** Comparison of clinical features of individuals with *HECTD1* missense and likely gene-disruptive variants

**Features (HPO code)**	**No. with missense variants affected**	**No. with likely gene-disruptive variants affected**
**Development**^a^		

Speech and language development (HP:0000750)	5/8	5/5
Fine motor development (HP:0010862)	5/8	3/4
Gross motor development (HP:0002194)	2/8	2/4
Intellectual disability (HP:0001249)	2/2	1/1

**Psychiatric**^a^		

ADD/ADHD (HP:0007018)	3/4	4/5
ASD (HP:0000729)	2/6	3/5
Anxiety (HP:0000739)	1/5	3/4
Self-injurious behavior (HP:0100716)	1/5	1/4
Hallucinations (HP:0000738)	1/5	1/4
Aggressive behavior (HP:0000718)	2/5	0/4

**Neurological**		

Epilepsy (HP:0001250)	5/9	1/4
Abnormal muscle tone (HP:0003808)	4/9	2/4
Gastrointestinal	–	–
Dysphagia (HP:0002015)	4/9	0/4
Tube feeding (HP:0033454)	5/9	0/4

**Other**		

Cryptorchidism (HP:0000028) in males	2/6	1/2

HPO, human phenome ontology; ADD, attention deficit disorder; ADHD, attention deficit/hyperactivity disorder; ASD, autism spectrum disorder.

a Not assessed in all due to age; individuals 10 (splice variant) and 14 (compound heterozygous variants) are included in the second column.

### Neurological and neurodevelopmental disability phenotypes

Eleven of the 14 individuals (79%) were reported to have varying degrees of NDDs (Table 2). Individual 9 was unable to be assessed due to death at age <6 months from multiple congenital anomalies, including severe pulmonary vein stenosis. Individual 6 had normal development at the last clinical update at 6 months of age. Individual 3 had global developmental delays that normalized by 3.5 years of age. NDDs primarily involved speech and language, with all but two persons of verbal age displaying impaired acquisition of language. The degree of language impairment was mild to moderate, with most speaking in single words and short phrases. Fine motor skills were impaired in 8/12 (67%) individuals. Gross motor skills were impaired in 4/12 (33%) individuals, and abnormal muscle tone was noted in 6/13 (46%).

### Behavioral and psychiatric phenotypes

Psychiatric outcomes were reported for 11 individuals who were older than 1 year. Of these, 10 (91%) carried one or more diagnoses of psychiatric or behavioral conditions. In addition to the developmental delays described above, nearly half carried a diagnosis of autism spectrum disorder (5/11, 45%). Seven individuals (7/11, 64%) were diagnosed with ADHD. Six individuals displayed behavioral issues (6/11, 55%), including aggression (2/9, 22%), auditory hallucinations (2/9, 22%), self-injurious behavior (2/9, 22%), and anxiety (4/9, 44%).

### Epilepsy

**Figure 2:**
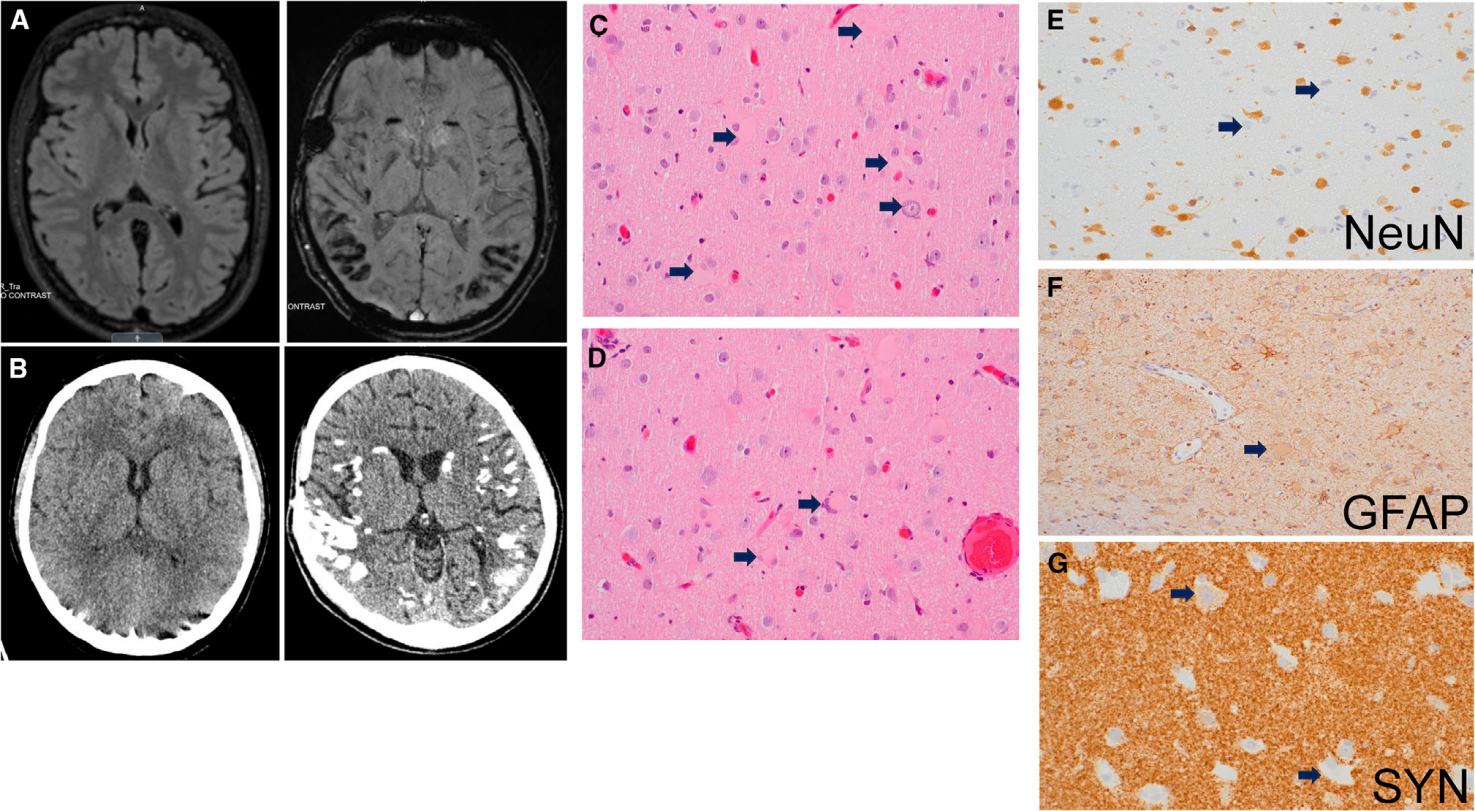
Cortical abnormalities in temporal lobe resected for drug-resistant epilepsy in individual 2 with *de novo HECTD1* p.Leu237Ser variant (A) Magnetic resonance and (B) computed tomographic brain images in an axial section comparing control (left) to individual 2 (right). (C and D) Hematoxylin and eosin-stained sections demonstrating dysmorphic and bi- to multinucleate balloon neurons with moderate to abundant glassy eosinophilic cytoplasm (arrows). (E–G) Immunostaining highlights architectural disarray, i.e., cortical dyslamination (NeuN) and weak labeling of dysmorphic/balloon neurons with glial fibrillary acidic protein (GFAP) and synaptophysin (SYN).

Six individuals in our cohort (6/14, 43%) developed epilepsy and had abnormal electroencephalograms (EEGs). Individual 1 had seizures and an abnormal EEG with rare left frontal sharp waves. Brain MRI demonstrated a 3-mm non-specific focus of increased T2 signal of the anterior left temporal lobe. Individual 2 began having focal seizures with dyscognitive features at 8 years of age. EEG demonstrated focal epileptiform abnormalities and seizure onset in the occipital and temporal lobes. Brain MRI demonstrated focal cerebral calcifications, multiple progressive subcortical white matter T2/FLAIR hyperintense foci, cystic encephalomalacia, and volume loss of the occipital lobes (Figures 2A and 2B). Cortical resection for intractable epilepsy demonstrated International League Against Epilepsy (ILAE) Type IIb focal cortical dysplasia characterized by the presence of dysmorphic and balloon neurons (Figures 2C and 2D) that were further variably highlighted by glial fibrillary acidic protein (GFAP), synaptophysin, and neuronal nuclei (NeuN) immunostainings (Figures 2E–2G). Individual 3 developed temporal lobe seizures, which were recorded on EEG; however, the brain MRI was normal. Individual 6 developed neonatal seizures on day 3 of life, characterized by staring and slow blinking, behavioral arrest, and apnea followed by profound desaturation and whole-body cyanosis. Two seizures, lasting between 1.5 and 2.5 min, were recorded on EEG, which demonstrated evolving sharp waves in the occipital regions, with spread into the left temporal region. Brain MRI on day 5 of life was unremarkable, demonstrating a trace (1–2 mm) right posterior convexity subdural hematoma. Individual 8 had seizure onset at 15 months old, with EEG demonstrating both generalized and bi-occipital interictal activity. The brain MRI was unremarkable, and the individual was treated with a vagal nerve stimulator for intractable epilepsy. Individual 11 had clinical seizures with EEGs showing mild diffuse background slowing as well as left occipital epileptiform discharges and brain MRI reportedly demonstrating right hippocampal atrophy. However, this individual also has a likely pathogenic variant in *DLL1* that also is associated with epilepsy.^36^ Individual 7 had abnormal movements in infancy but a normal EEG.

### Brain abnormalities

In addition to the variable cortical abnormalities identified for three of the six individuals with epilepsy described above, brain abnormalities were also reported at the age of 5 years old in individual 4, who had a small pars intermedia pituitary cyst and mild diffuse thickening of the corpus callosum but was not reported to have seizures. Three individuals had microcephaly.

### *Hectd1* is required for normal hippocampal and corpus callosum morphogenesis in a mouse model

**Figure 3:**
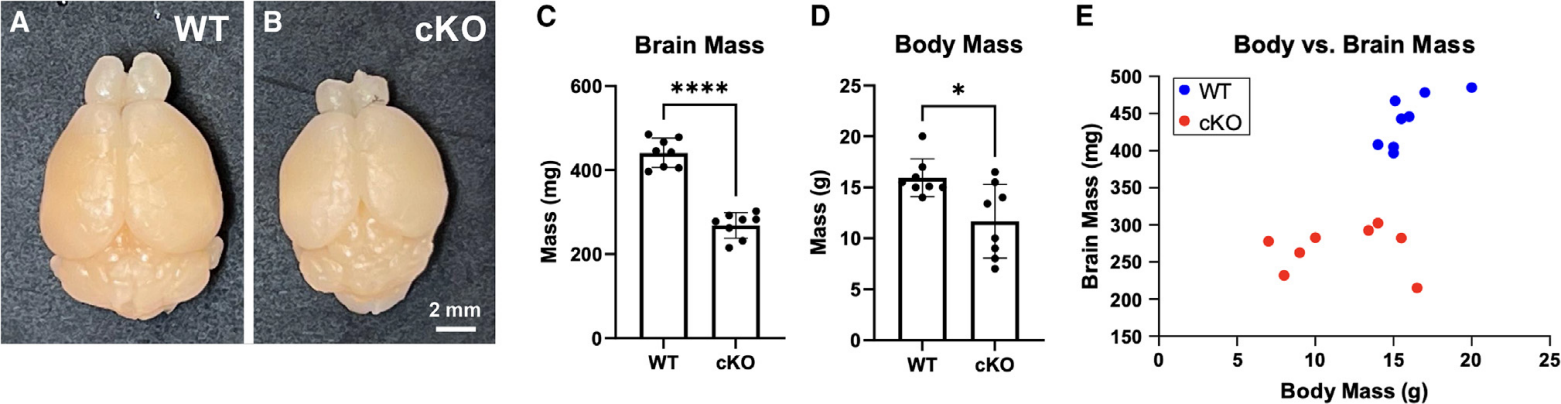
Sox1-Cre-mediated conditional knockout of *Hectd1* results in microcephaly (A and B) Representative wild-type (WT) and conditional KO brains showing gross microcephaly. (C) P30 cKO mice have significantly smaller brain mass compared to WT (*n* = 8, p < 1e−4). (D) P30 cKO mice have variable but significantly diminished body weight compared to WT (*n* = 8, *p* = 0.01). (E) No correlation between brain and body mass as all cKO brains are smaller, but only half of the mice had reduced body mass.

As the function of Hectd1 in the nervous system is currently unknown, we assessed whether mutations in *Hectd1* in the mouse resulted in structural brain abnormalities. The *Hectd1*^*opm*^ allele was generated in an ENU mutagenesis screen, which introduced a stop codon at amino acid 144 (p.Leu144Ter), resulting in a null allele.^7^^,^^8^ Homozygous *Hectd1* loss-of-function mutant mice die during mid-gestation due to placental and heart defects.^7^^,^^8^^,^^9^^,^^11^^,^^12^ To investigate the requirement of *Hectd1* for early mouse brain development, we generated a conditional knockout model using *Sox1-cre*-mediated recombination of a floxed *Hectd1* allele. *Sox1-cre* drives recombination in the neural lineage beginning at the neural plate stage.^25^^,^^40^ *Hectd1*^*flox/flox*^ females were mated with *Hectd1*^*opm/*+^;*Sox1-cre* males and the brains of litters examined. The brain mass of conditional knockout mice that survived to P30 was reduced by ∼30% compared to their wild-type littermates (*n* = 8, *p* < 0.0001) (Figures 3A–3C). In contrast, the body mass of *Hectd1*^*opm/flox*^;*Sox1-cre* mice at P30 was variable, with some conditional knockout pups weighing less than their littermate controls and others with similar weights (Figure 3D). Thus, microcephaly was constant and uncorrelated with body mass (Figure 3E).

**Figure 4:**
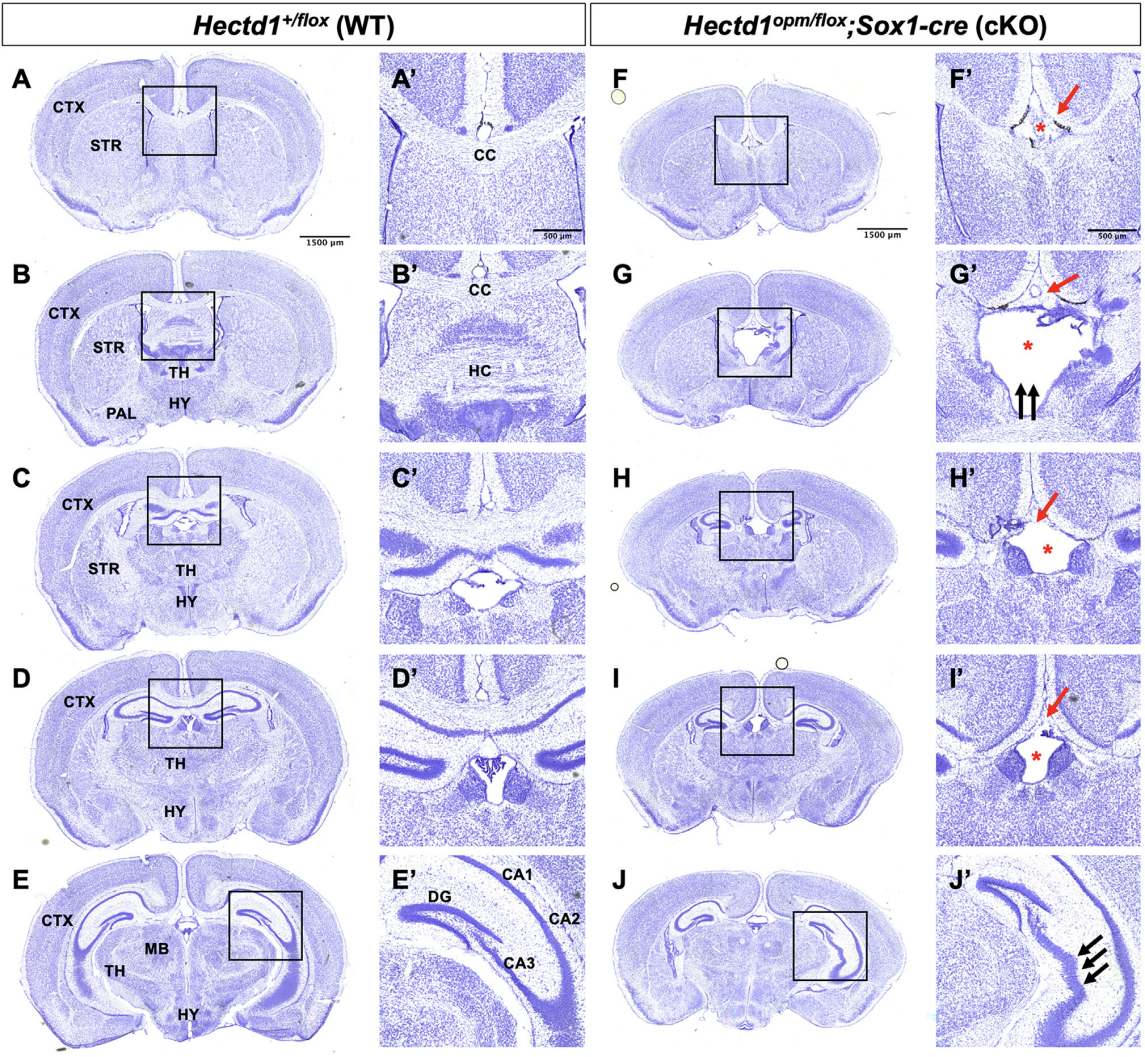
*Sox1-Cre*-mediated conditional knockout of *Hectd1* results in brain morphological phenotypes (A–E) Nissl staining of a P30 wild-type (WT) littermate brain shown in serial sections from rostral to caudal. (F–J) cKO sections are also shown from rostral to caudal. cKO brains appear smaller and exhibit several morphological abnormalities. Complete agenesis of the corpus callosum and ventricular dilation in cKO brain are shown by red arrow and star in (F′), (G′), (H′), and (I′). The CA3 hippocampal layer exhibits dysmorphology with abnormal bending and dispersion (black triple arrow in J′). Data are representative of seven cKO and three WT sectioned brains from 11 litters, respectively. CTX, cerebral cortex: STR, striatum; TH, thalamus; HY, hypothalamus; PAL, pallidum; CC, corpus callosum; HC, hippocampal commissure; MB, midbrain; DG, dentate gyrus; CA1, CA2, and CA3 subfields.

Compared to wild-type brain (Figures 4A–4E), histological analysis of surviving cKO P30 brains revealed significant morphological phenotypes that did not correlate with body mass (Figures 4F–4J). Complete agenesis of the corpus callosum was fully penetrant in the conditional knockout model (Figures 4F′–4I′) and was accompanied by other midline white matter tract defects, including dysgenesis of the hippocampal commissure and dilation of the third ventricle. The conditional knockout mice also exhibited abnormal organization of the hippocampal CA3 region that was most prominently observed in caudal sections (Figure 4J′). Corpus callosal and hippocampal dysgenesis were fully penetrant and consistent across all cKO mice (*n* = 7) (chi-squared = 10.0, degrees of freedom = 1, probability = 0.002).

### Functional analysis of *HECTD1* variants in *C*. *elegans*

To provide support of the human variants being damaging to HECD-1 function and to determine whether the genetic mechanism is consistent with heterozygous inheritance, we performed functional studies in *C*. *elegans*. We modeled two missense variants, p.Arg371Gly (individual 3) and p.Asp1116Asn (individual 5), and one nonsense variant, p.Gly1224Ter (individual 12), in the *C*. *elegans hecd-1*, which is the ortholog of *HECTD1*. These three variants had conserved amino acids at the corresponding locations in HECD-1 (Figure 1C), allowing CRISPR-Cas9 genome editing to precisely introduce the variants into the corresponding residues of *hecd-1* to generate p.Arg350Gly, p.Asp1239Asn, and p.Gly1345Ter variant edits in *C*. *elegans* (Figure S1A). We also generated control edits, p.Arg350Arg, p.Asp1239Asp, and p.Gly1345Gly, that are wild type at the variant residue in *C*. *elegans* but carry the same synonymous changes introduced during CRISPR-Cas9 editing to prevent Cas9 recleavage following homology-directed repair (Figure S1B). The premature termination codon, p.Gly1345Ter in HECD-1, located in the middle of the gene, likely leads to nonsense-mediated mRNA decay. A *hecd-1* null mutant (Δ) (*ok1437*) was also obtained from the CGC for comparison.

**Figure 5:**
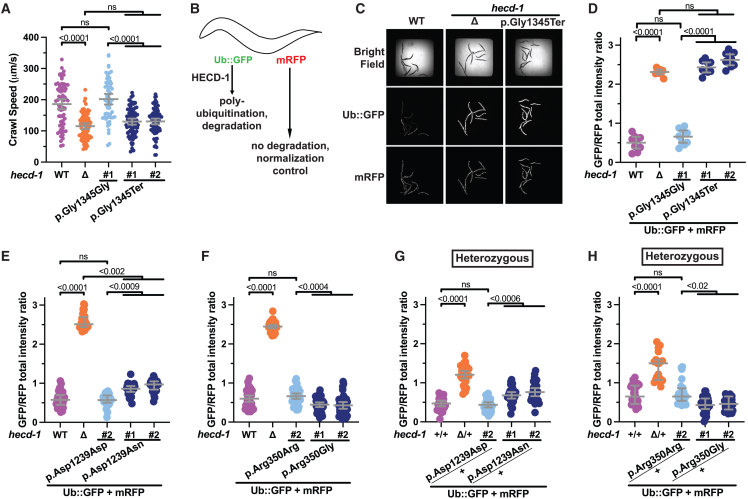
Functional analysis in *C*. *elegans* indicates that missense and nonsense variants are damaging to HECD-1 function (A) Crawl speed of homozygous wild-type (WT, purple), null (Δ) mutants (orange), control edit (light blue), and two independent CRISPR-edited variant lines with the p.Gly1345Ter variant (dark blue) worms on agar plates determined by WormLab (MBF Bioscience). Each dot represents an animal. *n* = 61–83 for each genotype. (B) Schematic showing how the Ub^G76V^-GFP (abbreviated as UbGFP) reporter assay works. HECD-1 mediates the polyubiquitylation of the mutant, UbGFP. The mutation targets GFP for proteasomal degradation but not mRFP protein, which serves as a normalization control. (C) Representative bright-field, GFP, and RFP images of UbGFP animals captured on the CX7 high-content imager (Thermo Fisher Scientific). (D–F) Quantification of UbGFP accumulation (expressed as a ratio of GFP/RFP) in homozygous *hecd-1* p.Gly1345Ter (D), p.Asp1239Asn (E), and p.Arg350Gly (F) variants. Each dot represents a well containing approximately ten animals. *n* = 9 wells for (D); *n* = 30–31 wells for (E); *n* = 35 wells for (F). (G and H) Quantification of UbGFP accumulation in heterozygous p.Asp1239Asn (G) and p.Arg350Gly (H) variants. *n* = 25–27 wells for (G); *n* = 19–32 wells for (H). Error bars display the mean plus 95% confidence interval in (A), (D), (F), and (G) or the median plus interquartile range in (E) and (H).

We evaluated the variant effects on *C*. *elegans* development and locomotion by measuring crawl speed and body length using WormLab (MBF Bioscience) (Figure S2A).^28^ The homozygous null mutant (Δ) and two independent homozygous nonsense p.Gly1345Ter variant strains (#1 and #2) displayed significantly decreased crawl speed (Figure 5A) and reduced body length (Figure S2B) compared to the wild type and p.Gly1345Gly controls. The null mutants and the p.Gly1345Ter variant animals also had significantly reduced thrashing (swimming) when placed in liquid (Figure S2C). The p.Gly1345Ter variant animals behaved similarly to the null mutant for the three phenotypes tested. No crawling, thrashing, or body length phenotypes were detected in the two missense variants, p.Arg350Gly and p.Asp1239Asn (Figures S2D–S2F).

To test whether the variants alter the known ubiquitin (Ub)-mediated protein degradative function of HECD-1,^31^^,^^41^^,^^42^ we utilized a previously described reporter, Ub^G76V^-GFP, for assaying polyubiquitylation and proteasomal degradation to measure the loss of *hecd-1* activity in *C*. *elegans*.^31^ In this assay, worms expressed an engineered non-cleavable variant of ubiquitin where the C-terminal Gly76 residue of Ub is mutated and fused to GFP (hereafter referred to as UbGFP) (Figure 5B), resulting in the accumulation of polyubiquitin chains and fusion to GFP protein, resulting in UbGFP being targeted to the proteasome for degradation.^31^^,^^43^ The reporter strain also co-expresses mRFP, which is not readily degraded by the UPS and serves as a normalization control. Proteasomal inhibition using bortezomib almost completely inhibited UbGFP degradation in wild-type animals, confirming that UbGFP was degraded by UPS (Figure S3). Moreover, Liu et al. also showed that loss of *hecd-1* in *C*. *elegans* led to a significant increase in accumulation of UbGFP and endogenous polyubiquitylated proteins.^31^ Consistent with these findings, UbGFP levels (expressed as a ratio of GFP/mRFP) were elevated in the *hecd-1* (Δ) null mutants (Figures 5C and 5D). Treatment of *hecd-1* null mutants with bortezomib did not increase UbGFP levels (Figure S3), indicating that HECD-1-mediated degradation of UbGFP was proteasome dependent. UbGFP was also elevated in the p.Gly1345Ter variant animals at a similar level as the null (Δ) mutants (Figures 5C and 5D). A modest but significant increase in UbGFP accumulation was detected in the p.Asp1239Asn variant homozygous animals (Figure 5E). In contrast, the UbGFP level in the p.Arg350Gly variant homozygous animals was lower than in the control (p.Arg350Arg) and wild-type animals (Figure 5F), contrary to that of the p.Gly1345Ter variant homozygotes. Together, these results suggest the p.Gly1345Ter, p.Asp1239Asn, and p.Arg350Gly variants are all damaging to HECD-1 function. Moreover, the magnitude and direction of the changes of the GFP/mRFP ratio suggest that the p.Gly1345Ter variant behaves like a loss-of-function allele, while the p.Asp1239Asn and p.Arg350Gly variants behave like a weak hypomorph and a change of function (possibly a hyperactive gain of function), respectively.

Accumulation of ubiquitin-tagged proteins can also trigger cellular stress and activate the UPR pathway,^44^ which can be studied in *C*. *elegans* using the *hsp-4p*::*GFP* reporter.^33^ *hsp-4p*::*GFP* expression was modestly upregulated in p.Gly1345Ter variant homozygous animals compared to the wild-type controls (Figure S2G). No upregulation of *hsp-4*::*GFP* was observed in the p.Asp1239Asn variant. In contrast, *hsp-4*::*GFP* was decreased in the p.Arg350Gly variant compared to the wild-type control (Figure S2G). The opposing phenotype of the p.Arg350Gly variant compared to the p.Gly1345Ter loss-of-function variant provides additional support for the p.Arg350Gly variant being a change-of-function allele.

To determine whether the genetic mechanisms of the variants in *C*. *elegans* are consistent with a dominant presentation in humans, we assessed the UbGFP phenotype in variant heterozygotes. The UbGFP fluorescence in the *hecd-1* null heterozygotes was significantly elevated compared to the wild-type controls, indicating that *hecd-1* is haploinsufficient in *C*. *elegans* for this phenotype (Figure 5G). Elevated UbGFP fluorescence was also detected in the p.Asp1239Asn heterozygotes, albeit to a lesser extent. In contrast, the UbGFP fluorescence was decreased in the p.Arg350Gly heterozygotes (Figure 5H), again supporting this variant being a change-of-function allele.

In summary, evaluation of the p.Arg350Gly, p.Asp1239Asn, and p.Gly1345Ter variants indicated that all three variants act dominantly and are damaging to HECD-1 function in *C*. *elegans*. The p.Gly1345Ter variant was indistinguishable from the null mutant (Δ) for the phenotypes tested. The p.Asp1239Asn variant was characteristic of a weak hypomorphic allele, while the p.Arg350Gly variant behaved as a change-of-function variant. All variants showed phenotypes in heterozygous *C*. *elegans*. By extension, the human p.Arg371Gly (individual 3), p.Asp1116Asn (individual 5), and p.Gly1224Ter (individual 12) variants are likely damaging to *HECTD1* function and may act dominantly. Our data suggest that the human p.Arg371Gly variant may behave as a gain of function, while the p.Asp1116Asn and p.Gly1224Ter variants may behave in a haploinsufficient manner. While we did not specifically test the compound heterozygous variants found in individual 14, this individual had a frameshift variant inherited from an apparently healthy parent, which may suggest incomplete penetrance of the dominant inheritance in humans as well as the potential for additive effects of two damaging alleles.

### Enrichment of *HECTD1* variants in published NDD and CHD cohorts

To further investigate the role of *HECTD1* variants in human disease, we performed an enrichment analysis of *de novo* variants in an independent cohort of 53,305 unique individuals diagnosed with either NDDs or CHD within published cohorts (Table S4) whose trio-based sequencing results are publicly available (Table S5). Only missense or likely gene-disruptive (stop-gained, splice-donor, splice-acceptor, frameshift) variants in *HECTD1* were included.^45^ There were 12 missense and two likely gene-disruptive *de novo* variants in *HECTD1* in these published cohorts (Table S6). To test for enrichment, variants were analyzed with denovolyzeR.^46^ These results show a 1.65-fold enrichment in *de novo* variants in *HECTD1* in the aggregate published cohorts using the denovolyzeR analysis (*p* = 0.05) (Table S7) and significant enrichment (4.02 × 10^−6^) in the chimpanzee-human *de novo* variant test (Table S8), which incorporates locus-specific transition/transversion/indel rates and chimpanzee-human coding sequence divergence to estimate the expected *de novo* rates.^47^ Both statistical tests showed greater enrichment of *HECTD1* missense variants than likely gene-disruptive variants.

## Discussion

Our data reveal a critical role for *HECTD1*, a key mediator of the UPS pathway, in neural development and a genetic cause of human NDDs. Individuals with *HECTD1* variants exhibit a range of phenotypes, including neurodevelopmental disability, autism, ADHD, and epilepsy. In addition to our discovery of *de novo* likely gene-disruptive and missense variants and our demonstration of enrichment of *HECTD1* variants in published cohorts, several metrics of genomic constraint from gnomAD population data support *HECTD1* as a disease-causing gene.^14^ First, *HECTD1* is constrained for missense variantion with a missense *Z* score of 6.43. Second, HECTD1 is constrained for loss-of-function variation with an observed/expected ratio of 0.27, indicating that it is likely a haploinsufficient gene.^22^ In addition to the 14 individuals described here, at least four affected individuals in the DECIPHER database have <4 Mb deletions that include *HECTD1*, with no other known NDD genes located within the deleted interval.^48^ Cross-disorder dosage sensitivity maps compiled from rare copy number variant data also support *HECTD1* as having a high probability of being both haploinsufficient (0.99) and triplosensitive (0.99).^49^

*HECTD1* regulates numerous pathways that may contribute to NDDs, including signal transduction pathways essential for brain development, such as Notch, Wnt, retinoic acid, and estrogen receptor signaling.^41^^,^^50^^,^^51^^,^^52^^,^^53^ *HECTD1* regulates the striatin-interacting phosphatase and kinase (STRIPAK) complex, chromatin remodeling, cholesterol export, and protein translation,^10^^,^^42^^,^^50^^,^^54^^,^^55^^,^^56^ influencing proliferation, apoptosis, autophagy, DNA-damage repair, and mitochondrial function.^55^^,^^56^^,^^57^^,^^58^^,^^59^^,^^60^^,^^61^^,^^62^ *HECTD1* is also involved in inflammation in astrocytes, microglia, and other cell types and can regulate synaptic function by regulating the expression of glutamate transporter 1 (GLT-1) in astrocytes.^59^^,^^63^^,^^64^^,^^65^^,^^66^ Effects on human development are not surprising given that *hecd-1*, the *C*. *elegans* ortholog of human *HECTD1*, also regulates Notch signaling, chromatin remodeling, and the expression of developmentally critical genes via the switch/sucrose non-fermentable (SWI/SNF) chromatin remodeling complex and katanin in mitosis and meiosis, thereby impacting the cell cycle.^41^^,^^42^^,^^56^

Additional individuals will need to be identified to establish genotype-phenotype correlations with specific *HECTD1* variants, particularly as this large protein regulates multiple substrates and pathways described above. While our conclusions are limited by the small number of variants we studied, our identification of variants that increase and decrease ubiquitylation in *C*. *elegans* suggests that there may be multiple genetic mechanisms of disease pathogenesis, including change of function and loss of function. While we saw no apparent phenotypic differences between individuals with missense variants and those with likely gene-disruptive variants, the one individual we identified with compound heterozygous inheritance had growth impairment along with NDD. Notably, this child inherited the missense and frameshift variants from apparently healthy parents, suggesting that genetic or environmental modifiers may be required to develop the phenotype. Microcephaly was present in this individual and two others, which may indicate more severe impairment in HECTD1 function, as seen with *Hectd1* deletion in the brain of the conditional mouse model. Although not all individuals underwent brain imaging, the imaging abnormalities were heterogeneous and more subtle than the striking hippocampal and callosal abnormalities observed in the conditional mouse model, likely due to the presence of a wild-type allele.

The phenotypic variability, ranging from mild speech delay to multiple congenital anomalies, suggests that individual variants may alter specific HECTD1 interactions with substrates and co-factors or that genetic or environmental modifiers contribute to these differences. For example, in mice, a heterozygous mutation of *Hectd1* interacts with vitamin A deficiency to modify aortic arch development.^12^^,^^52^ Congenital cardiac anomalies were present in only two of our 14 individuals, suggesting a higher penetrance of NDD phenotypes. Prior data from a study of >7,000 individuals with congenital heart disease described a single individual with compound heterozygous splice variants in *HECTD1*,^13^ and our recent study identified rare *HECTD1* variants in individuals with NTDs.^14^ Unfortunately, the co-occurrence of NDD phenotypes in the small number of individuals with CHD or NTDs and *HECTD1* variants was not explored in the prior studies, likely due to their ascertainment in infancy. Neural tube defects are notorious for complex oligogenic patterns of inheritance, where multiple genetic variants interact with environmental factors, and *HECTD1* has been proposed to contribute to human NTDs in this manner.^67^^,^^68^ In aggregate, the human and mouse data demonstrate a requirement for *HECTD1* for neural tube, hematopoietic stem cell, heart, brain, and placental development^7^^,^^8^^,^^9^^,^^10^^,^^11^^,^^12^ and suggest that *HECTD1* contributes to critical aspects of multiple early developmental processes.

### Conclusions

We report here data indicating a role for *HECTD1* variants in neural development, including significant enrichment of *HECTD1 de novo* variants in an independent cohort of 53,305 published individuals with NDDs and CHD as well as brain abnormalities in conditional *Hectd1* mutant mice. Our investigation of a limited number of variants in a *C*. *elegans* model supports likely dominant inheritance with both change-of-function and loss-of-function/haploinsufficient mechanisms. Further work is needed to identify genotype-phenotype correlations for *HECTD1* variants, particularly because of the potential for distinct mechanisms caused by individual missense variants.

## Data and code availability

This study did not generate or analyze datasets.

## Acknowledgments

We thank the individuals and their family members who participated in this study.

Research reported in this publication was supported by the Washington University Institute of Clinical and Translational Sciences (UL1TR002345) from the National Center for Advancing Translational Sciences of the National Institutes of Health and the Eunice Kennedy Shriver National Institute of Child Health & Human Development (NICHD) of the National Institutes of Health under award number P50HD103525 to the Intellectual and Developmental Disabilities Research Center at Washington University. Support was also provided by R01HD110556 from NICHD (S.C.P. and colleagues L.S.-K., A.N.J., K.L.K., and D.M.O.) and by the 10.13039/100009340Children’s Discovery Institute, Brendan’s Buddies, and St. Louis Children’s Hospital Foundation (G.A.S. and S.C.P.). This work was supported by R01HD098861 from NICHD (I.Z.) and the molecular imaging core of the District of Columbia Intellectual and Developmental Disabilities Research Center (DC-IDDRC) award P50HD105328 from NICHD. One individual was sequenced by UCI-GREGoR funded by the National Human Genome Research Institute of the 10.13039/100000002National Institutes of Health through the following grant, as part of GREGoR Consortium: 1U01HG011745. One individual underwent genome sequencing through the International Precision Child Health Partnership Gene-STEPS study, supported by the Boston Children’s Hospital Children’s Rare Disease Collaborative, the One8 Foundation, and the Robinson Family Initiative for Transformational Research. We thank Suk Regmi for technical assistance.

## Author contributions

E.O., H.D.H., T.K., W.Y., J.G.C., K.I., S.S., K.L., B.W., and I.E.Z. undertook laboratory work and/or data analysis. G.Z.-J., E.O., H.D.H., T.S., S.C.P., I.E.Z., and C.A.G. conceived and led the study. G.Z.-J., E.O., T.N.T., J.G.C., S.C.P., I.E.Z., and C.A.G. wrote the manuscript. The other authors contributed individual data and case reports. All authors read and approved the manuscript prior to submission.

## Declaration of interests

K.G.M. and M.J.G.S. are employees of GeneDx, LLC.

## Web resources

NCBI RefSeq database, https://www.ncbi.nlm.nih.gov/refseq/.

## Supplemental information

Document S1. Figures S1–S3, Tables S1–S8, and supplemental case reports

Document S2. Article plus supplemental information
