# Supplementary material for: Sequence variants in HECTD1 result in a variable neurodevelopmental disorder
Source: Am J Hum Genet. 2025 Jan 28;112(3):537–53. doi: 10.1016/j.ajhg.2025.01.001 (PMC11947180; doi:10.1016/j.ajhg.2025.01.001)
Supplement: Document S1. Figures S1–S3, Tables S1–S8, and supplemental case reports [file mmc1.pdf]

The American Journal of Human Genetics, Volume *112*

Supplemental information

Sequence variants in *HECTD1* result in a variable neurodevelopmental disorder

Gazelle Zerafati-Jahromi, Elias Oxman, Hieu D. Hoang, Wu-Lin Charng, Tanvitha Kotla, Weimin Yuan, Keito Ishibashi, Sonia Sebaoui, Kathryn Luedtke, Bryce Winrow, Rebecca D. Ganetzky, Anna Ruiz, Carmen Manso-Basúz, Nino Spataro, Peter Kannu, Taryn Athey, Christina Peroutka, Caitlin Barnes, Richard Sidlow, George Anadiotis, Kari Magnussen, Irene Valenzuela, Alejandro Moles-Fernandez, Seth Berger, Christina L. Grant, Eric Vilain, Gudny A. Arnadottir, Patrick Sulem, Telma S. Sulem, Kari Stefansson, Shavonne Massey, Natalie Ginn, Annapurna Poduri, Alissa M. D'Gama, Rozalia Valentine, Sara K. Trowbridge, Chaya N. Murali, Rachel Franciskovich, Yen Tran, Bryn D. Webb, Kim M. Keppler-Noreuil, April L. Hall, Bobbi McGivern, Kristin G. Monaghan, Maria J. Guillen Sacoto, Dustin Baldridge, Gary A. Silverman, Sonika Dahiya, Tychele N. Turner, Tim Schedl, Joshua G. Corbin, Stephen C. Pak, Irene E. Zohn, and Christina A. Gurnett

Case Reports

Individual 1

Individual 1 is a 10 year old female with epilepsy, autism and language delay. She was born at 37 weeks gestational age via C-section due to maternal diabetes. Growth parameters at the time of last evaluation at 9 years of age included OFC of 55.5cm (>97%ile), weight of 80.7kg (100%ile) and height of 145.4cm (86%ile). She has been diagnosed with obesity. Hearing and vision assessments were normal. Her first words were at 3 years of age, and presently she does not speak in full sentences. She has also been diagnosed with autism. She receives PT, OT, ST and also has an IEP at school which includes special education. She has frontal lobe epilepsy, with EEG demonstrating left frontal focal epileptiform abnormalities. Brain MRI demonstrated a 3mm area of T2 signal abnormality in the inferior left temporal lobe. Cardiac evaluation was normal.

Individual 2

Individual 2 is a 28 year old male who presented with focal onset status epilepticus at age 8. He was diagnosed with ADHD and mild cognitive impairment prior to the onset of epilepsy. He had short stature but otherwise normal growth parameters. By age 9, he developed drug resistant epilepsy with seizures arising from the left temporal lobe. MRI showed bilateral posterior temporooccipital lobe cerebral calcifications and white matter T2/FLAIR hyperintense foci involving the left temporal lobe. Left temporal lobe resection at age 9 for intractable epilepsy demonstrated ILAE Type IIb focal cortical dysplasia characterized by presence of dysmorphic and balloon neurons (Figure 2). After surgery, there were no further generalized tonic clonic seizures, but other seizures continued. His course has subsequently been characterized by progressive neurocognitive dysfunction, epilepsy, stroke-like episodes particularly of the occipital lobes, cortical visual impairment, suspected transient ischemic attacks with vision loss, weakness or right facial droop. His seizures are characterized by (1) visual hallucinations lasting up to hours with retained awareness, (2) staring and unresponsiveness, and (3) rare generalized tonic clonic activity. He has transient episodes of unclear etiology with sudden full loss of vision in both eyes or inability to walk lasting several minutes with nearly complete resolution. Brain MRIs have shown progressive occipital white matter hyperintensities and subcortical white matter T2/FLAIR hyperintense foci that progressively evolved over time to cystic encephalomalacia with volume loss of the occipital lobes (Figure 2). In addition to multiple antiepileptic medications, he was treated with IVIG and mycophenolate mofetil which decreased the frequency of the episodes for a presumed autoimmune condition despite negative autoantibodies. He also has kidney stones with end stage kidney failure, and bradycardia of unclear etiology with normal echocardiogram. He is employed part-time at a grocery store.

Individual 3

Individual 3 is a 5-year-old male with epilepsy, mild global developmental delay and persistently elevated transaminases. He was born at 37 weeks via induced vaginal delivery. Growth parameters at delivery included weight of 3232g (74%ile, Z-score 0.63) and length of 45.7cm (14%ile, Z-score -1.06). At time of last evaluation at 3 years of age, OFC was at 20%ile (Z-score 0.86), weight was at 29%ile (Z-score 0.55) and height was at 15%ile (Z-score 1.00). No facial dysmorphisms were noted. Hearing and vision evaluations were normal. However, developmental abnormalities were reported in all domains, requiring physical therapy and speech therapy. He walked at 15 months, but he was noted to have imbalance out of proportion to his age. He demonstrated left hand preference at 12

months of age. He said his first word at 15 months of age and was not speaking in full sentences at 20 months old, however speech rapidly normalized with placement of myringotomy tubes. He also required G-tube feedings until 3 years of age. He developed temporal lobe epilepsy, with EEG demonstrating seizures arising from the left temporal lobe at one month of age, with clinical semiology of spasticity and cyanosis. Brain MRI, however, was normal. At the time of last evaluation at 3.5 years of age, he had complete seizure control and normalization of the EEG on levetiracetam, as well as normal development. Bloodwork demonstrated elevated transaminases, therefore liver biopsy was performed, demonstrating abnormal mitochondria on electron microscopy, absence of complex I activity, decreased complex IV activity, and abnormal complex V assembly. Cardiac evaluation was normal.

Individual 4

Individual 4 is a 7 year old girl with global developmental delay, behavioral problems as well as movement disorder. She was born at 41 weeks gestational age by vaginal delivery. She was noted to be macrocephalic at birth and weighed 3940g (74%ile). At the time of last evaluation, OFC was 54cm (>97%ile), weight was 29.8kg (92%ile), and height was 124cm (67%ile). On exam she was noted to have bitemporal narrowing, high palate and hypotonia. She demonstrated poor latch and choking in infancy, which progressed to oral dysphagia by age two, thereby necessitating placement of a G-tube by which she receives all nutrition. The most recent swallow evaluation within the last year was normal. She was also diagnosed with severe central and obstructive sleep apnea at 14 months of age. She underwent tonsillectomy and adenoidectomy but was not able to tolerate BiPAP. Presently her central sleep apnea has mostly resolved. Although she walked on time around 13-14 months of age, she is noted to be clumsy and displays toe walking. She does not use any tools such as utensils. She spoke her first words around 12 months of age but subsequently stopped gaining new words, and now is non-verbal aside from the use of an augmentative communication device for single word communication. She was initially diagnosed with sensorineural hearing loss, however at age 4 was found to have normal hearing at which point hearing aids were discontinued. She has been diagnosed with severe autism and has self-injurious behaviors, intense tantrums, rigidity, aggression, poor emotional regulation, and poor frustration tolerance. She also displays stereotypic movements diagnosed as possible tic disorder. She receives PT, OT, ST and ABA, and also receives special education. Brain MRI at 5 years old demonstrated stable small pars intermedia pituitary cyst, as well as mild diffuse thickening of the corpus callosum. EEG at 5 years old was normal. At age 7, she had 3 short events concerning for seizure activity characterized by eyelid fluttering, and 1 event with lack of awareness, incontinence and full body shaking. Repeat EEG was again normal. She is not prescribed any anti-seizure medications and has not had any further events. The most recent brain MRI at 7 years old demonstrated mild under rotation and dysgenesis of the left hippocampus, which was felt to be unrelated to these events. ECHO and EKG were normal. Holter monitor demonstrated sinus bradycardia.

Individual 5

Individual 5 is an 18 year old male with mild intellectual disability and language impairments. He was born at 40 weeks by vaginal delivery. Growth parameters at birth included weight of 3317g (29%ile, Z-score -0.54). He had poor latch in infancy, and as an older child has frequent GI symptoms concerning for irritable bowel syndrome. He was noted to have fine motor difficulties as well as difficulties with verbal communication. Hearing and vision evaluations were normal. He has also been

diagnosed with ADHD and mild intellectual disability, although he did not receive therapy services or special education. At the time of the last evaluation at 18 years of age, OFC was 53cm (<3%ile), weight was 57kg (13%ile), and height was 185cm (89%ile). Malar hypoplasia, prominent jaw, arachnodactyly, pectus carinatum, pes planus, mild kyphosis, and cryptorchidism were noted on exam. Brain MRI was normal. ECHO demonstrated mild dilation of the ascending aorta. Marfan/LDS panel was sent, demonstrating paternally-inherited VUS in COL5A1. Bloodwork demonstrated decreased level of IgA.

Individual 6

Individual 6 is a 9 month old full-term boy with epilepsy and sleep apnea. He was born at 40 weeks gestational age by spontaneous vaginal delivery. Growth parameters at delivery were OFC of 36cm (75%ile), weight of 3480g (43%ile) and length of 47cm (3%ile). At 2 months of age, OFC was 40.5cm (65%ile), weight was 4920g (31%ile) and length was 57.5cm (40%ile). Exam demonstrated mildly low axial tone. No facial dysmorphisms were noted on exam. He passed a hearing screening on day of life 1. On day of life 3 he developed focal seizures characterized by staring, paucity of movement, apnea, profound desaturation and whole-body cyanosis. EEG captured 2 focal seizures arising from the left occipital region which evolved to spread into the left temporal region. Brain MRI on day of life 5 demonstrated trace right posterior convexity subdural hematoma but was otherwise unremarkable. Sleep study at 15 days old demonstrated moderate to severe central apnea and mild obstructive apnea, requiring continuous supplemental oxygen by nasal cannula. Modified barium swallow study at 16 days old demonstrated aspiration, which resolved with use of preemie flow nipple. As of the most recent clinical update at 6 months of age, he was weaned off supplemental oxygen and thus far his development has been normal.

Individual 7

Individual 7 is a 9 month old male with history of Tetralogy of Fallot status post cardiac surgery, Pierre Robin sequence (with Veau class II cleft palate), and dysmorphic features. Pregnancy was complicated by mother with type 2 diabetes mellitus and obesity. Prenatal history is notable for diagnosis of a congenital heart defect after 20-week anatomy scan and fetal echocardiogram. He was born via C-section at 38 weeks gestational age due to breech presentation, prolonged rupture of membranes, and known congenital heart disease. Growth parameters at delivery included weight of 3.18kg (36 %ile), length of 50cm (52%ile) and OFC of 33cm (12 %ile). He was diagnosed with Tetralogy of Fallot (TOF) with severe pulmonary vein stenosis and small right ventricular outflow tract. His first cardiac surgery was at 2 weeks of age, with subsequent repair of TOF completed at 4 months of age. He also was found to have Pierre-Robin sequence with Veau class II cleft palate and had reflux and aspiration. Newborn hearing screen was normal. At 3 weeks of age a G-tube was placed, a right inguinal hernia was repaired, and left orchioplexy was attempted but was not successful. At 5 weeks of age, a hip ultrasound revealed congenital bilateral dislocation of the hips. At 7 weeks of age, a supraglottoplasty was performed due to laryngomalacia. At 6 months old, OFC was 40.2 cm (O.44%, Z=-2.62), weight was 6.18 kg (0.32%, Z=-2.72), and height was 65.8 cm (4.75%, Z=-1.67). Dysmorphic features included microcephaly, large, simple, and protruding ears, blue sclera, downslanting palpebral fissures, epicanthal folds, long philtrum, a small, downturned mouth, and microretrognathia. He had prominent truncal and axial hypotonia and was unable to roll over or push himself up when prone. He is G-tube dependent. Due to concerns for abnormal movements an EEG was obtained, which was normal. At 8 months old, a sleep study revealed sleep apnea with tongue

obstruction. At 9 months of age, he underwent bilateral mandibular distractor placement for mandibular distraction osteogenesis prior to cleft palate repair. He has gross motor delays and is unable to sit unsupported. He has fine motor delays and is now starting to reach for objects with one hand. He vocalizes with different vowel sounds and is receiving speech therapy to improve lingual movement and support oral motor skills.

Individual 8

Individual 8 is a 9 year old female with epilepsy, ADHD and psychiatric abnormalities. She was born via C-section at 40 weeks gestational age. Birth weight was 3260g (38%ile) and length was 48.3cm (17%ile). At time of last evaluation at 9 years of age OFC was 51.5cm (~62.5%ile), weight was 25.8kg (25%ile) and height was 129cm (26%ile). She developed epilepsy at 15 months of age, necessitating VNS placement. Several EEGs have demonstrated both generalized and focal interictal activity. Brain MRI at around 5 years old was unremarkable. She has been diagnosed with ARFID and failure to thrive, necessitating G-tube placement. She also has ADHD, anxiety, depression, and behavioral outbursts. She receives occupational therapy for fine motor difficulties. She has an IEP at school which includes special education and is currently performing at the kindergarten level despite being in second grade for the second time.

Individual 9

Individual 9 is a former 31 week gestational age male who died at 6 months of age. He was delivered via C-section due to prolonged rupture of membranes and non-reassuring fetal heart tones. At birth OFC was 30.7cm (77%ile), weight was 2720g (99%ile) and length was 44.5cm (79%ile) based on Fenton growth charts. A hearing assessment was normal. On exam he was noted to have dysconjugate gaze, hypertelorism, long eyelashes, upturned ears with fleshy lobes and mid-lobe indentation, a short, upturned nose, and wide philtrum with deep grooves. At 6 months of age, OFC was 45.5cm (89%ile), weight was 9.8kg (96%ile) and length was 61.2cm (0.51%ile) based on CDC growth charts. He had numerous congenital anomalies including severe bilateral pulmonary vein stenosis, enlarged left kidney with cortical cyst, 8th thoracic hypoplastic butterfly vertebrae, duodenal atresia, esophageal atresia with tracheoesophageal fistula, biliary atresia with absent gallbladder, and imperforate anus with rectocutaneous fistula. He ultimately underwent a Kasai procedure for biliary atresia, tracheostomy, G-tube placement and colostomy. Serial head ultrasounds demonstrated mildly prominent lateral and third ventricles and enlarged extra-axial spaces with evolving left caudothalamic subependymal hemorrhage. He subsequently was diagnosed with portal hypertension, cholestasis, necrotizing enterocolitis, ascites, and abdominal abscesses. A CMA and karyotype were normal. A cholestasis gene panel demonstrated likely pathogenic variant in *TTN* gene. He died at 6 months of age due to pulmonary hypertension secondary to inoperable bilateral pulmonary vein stenosis.

Individual 10

Individual 10 is an 18 year old male with language delays, ADHD and autism. He was born at term after induction of labor. Growth parameters at delivery included weight of 3460g (41%ile) and length of 51cm (47%ile). Audiology evaluation at 5 years of age was normal. At the time of the last evaluation at 9 years old, his weight was 27.2kg (38%ile) and height was 138.2cm (77%ile).

Individual 11

Individual 11 is a 6 year old female with epilepsy, autism and speech delay. She was born at 39 weeks gestational age by vaginal delivery. At birth, she weighed 2920g (24.1%ile). At the most recent evaluation at 5 years of age, OFC was 48.2cm (2.78%ile), weight was 17kg (35%ile) and height was 109cm (61%ile). Exam was notable for deep-set eyes, long philtrum and full cheeks. In terms of development, she was noted to have speech delay necessitating speech therapy. She was ultimately diagnosed with autism. She also displays disruptive behaviors at school and labile mood. Presently she is in a mixed classroom with mainstream and special education support. She has been diagnosed with epilepsy, with EEGs demonstrating left occipital epileptiform discharges as well as mild diffuse background slowing. Brain MRI reportedly demonstrated right hippocampal atrophy.

Individual 12

Individual 12 is a 19 year old female with global developmental delay, intellectual disability and ADHD. She was born at 37 weeks via vaginal delivery. Weight at delivery was 2140g (5%ile, Z-score -1.66). She was noted to have gross motor delay and severe language delay, did not walk until 20 months of age and did not speak until 2 years old. She was ultimately diagnosed with mild intellectual disability requiring special education and speech therapy. She also has fine motor difficulties. She was diagnosed with ADHD and noted to have several psychiatric abnormalities including anxiety, self-injurious behavior and behavioral disorder. At the time of the last evaluation at 19 years of age, OFC was 55cm (50%ile), weight was 46.1kg (5%ile, Z-score -1.62), and height was 158.9cm (8.7%ile, Z-score -1.36). Facial dysmorphisms were noted, including long face, broad forehead, epicanthus and hypertelorism with low set ears, narrow palate, bulbous nose and thick lips. Evaluation of hearing was normal, however she was noted to have strabismus. Brain MRI was normal.

Individual 13

Individual 13 is a 10 year old male with autism, ADHD, language delay, and psychiatric abnormalities. He was born at 40 weeks gestational age. Weight at time of delivery was 3345g (32%ile) and length was 49.5cm (23%ile). Exam demonstrates cryptorchidism as well as wide nasal bridge, broad nasal tip, and a long upper lip. He demonstrated delayed language acquisition for which he required speech therapy. His first words were at 2 years of age and he did not speak in sentences until around 4 years of age. He also displayed fine motor delays. He was diagnosed with autism, ADHD, anxiety, and also has auditory hallucinations. He is currently receiving special education.

Individual 14

Individual 14 is a 5 year old girl with global developmental delay and ADHD. She was born at 38 weeks gestational age following an uncomplicated delivery. Growth parameters at birth included OFC of 32cm (13%ile), weight of 2530g (12%ile) and length of 41cm (0%ile). At the time of last evaluation at 4 years of age, OFC was 42.5cm (<3%ile), weight was 9.9kg (0%ile), and height was 84.4cm (0%ile). She is currently undergoing evaluation for growth hormone therapy. Exam at that time demonstrated mildly low hanging columella, proximally placed thumb, and 5th finger clinodactyly. She has had normal cardiac evaluation, renal ultrasound, and brain MRI. She demonstrated delays in all domains and requires PT and OT. She has been diagnosed with ADHD which is being treated with stimulant medication as well as an IEP at school.

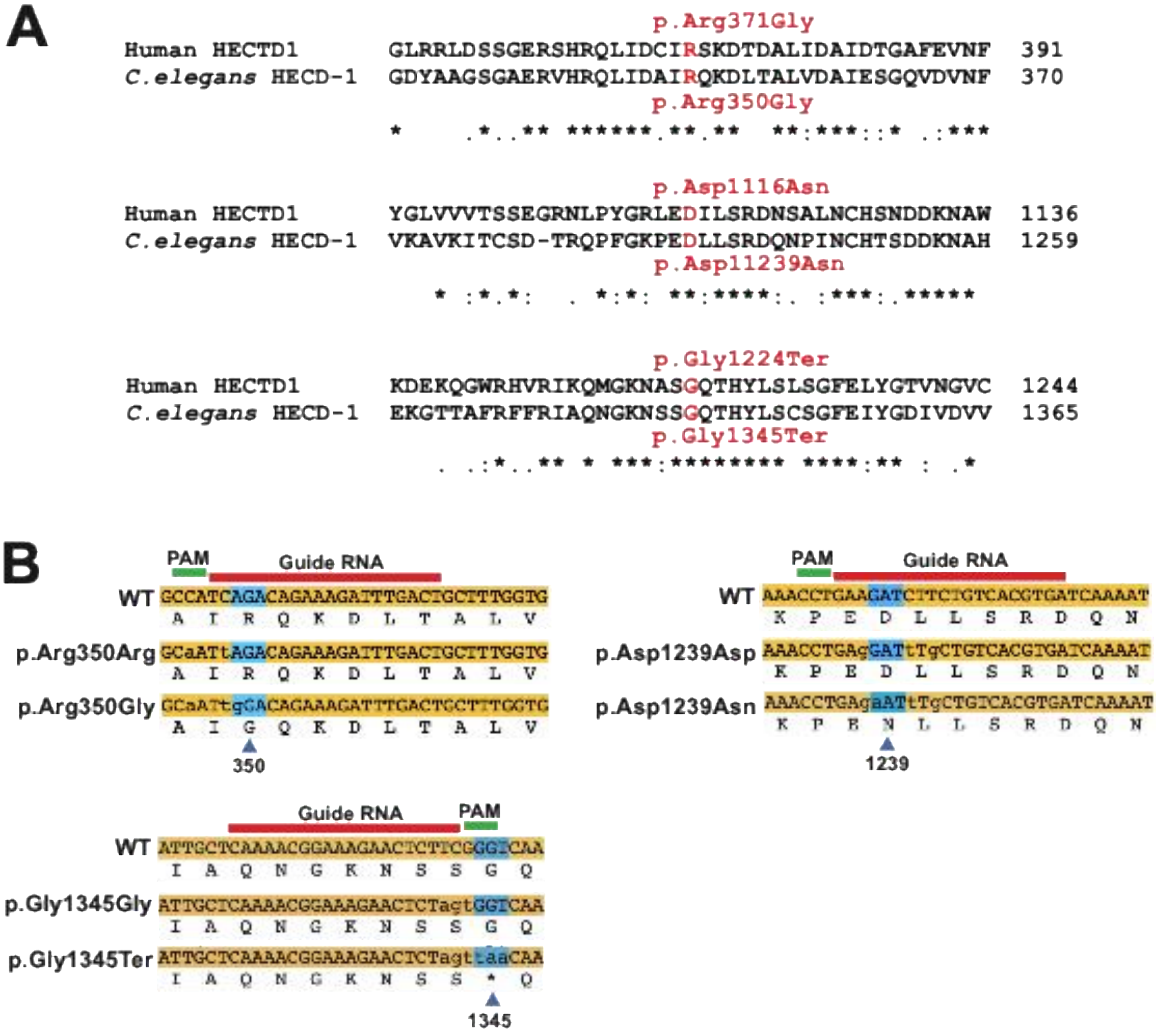

Figure S1. Comparison of human *HECTD1* and *C. elegans* HECD-1 and CRISPR/Cas9 editing details.

(A) The *C. elegans* ortholog of *HECTD1* is *hecd-1*. Human *HECTD1* variants p.Arg371Gly, p.Asp1116Asn and p.Gly1224Ter correspond to *C. elegans* HECD-1 p.Arg350Gly, p.Asp1239Asn, and p.Gly1345Ter, respectively. (B) CRISPR/Cas9 genome editing details. Variant residues are highlighted in *blue* and indicated by an *arrowhead*. Guide RNAs are indicated with a *red bar*. PAM sites are indicated with a *green bar*. Synonymous changes introduced during the CRISPR/Cas9 editing to disrupt gRNA binding following homology directed repair and facilitate allele genotyping are shown in *lower case*.

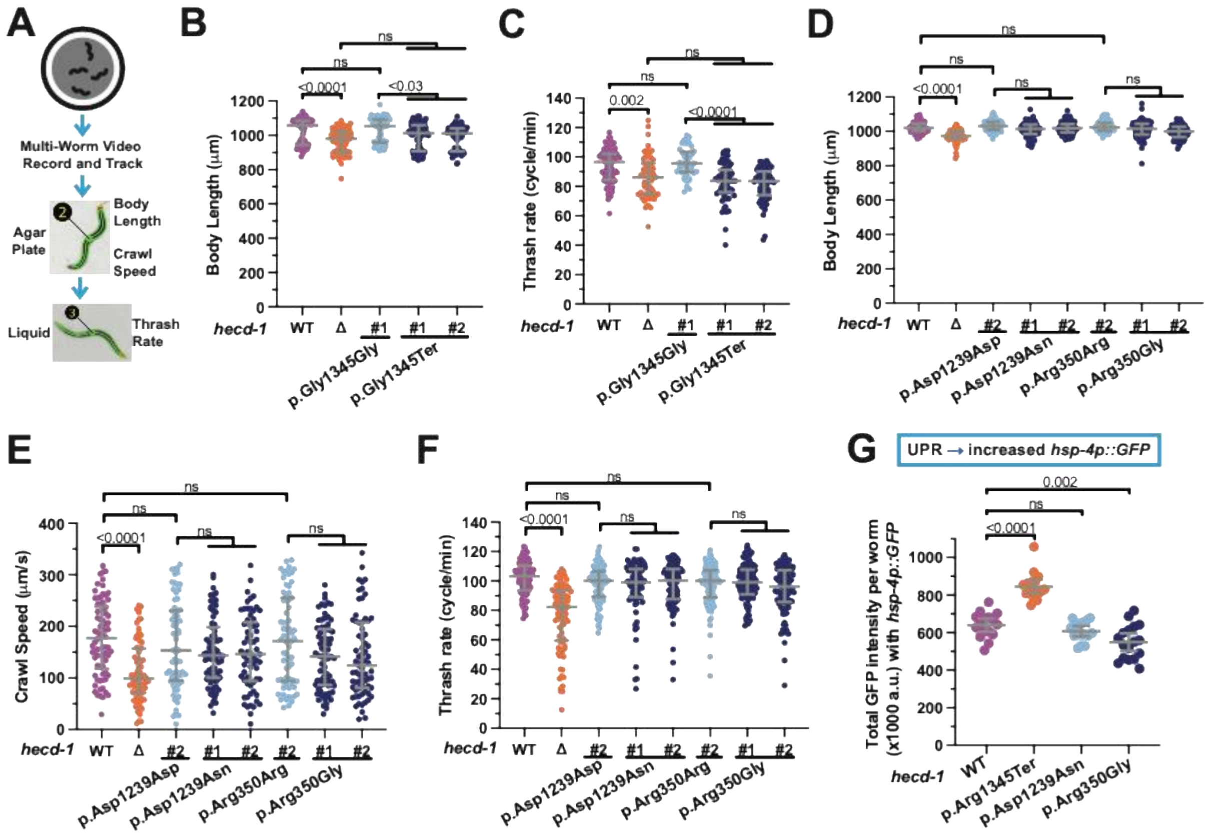

Figure S2. Phenotypic analyses of *hecd-1* variants in *C. elegans*
(A) A schematic of the WormLab workflow. One-minute videos of multiple worms crawling on an agar plate and thrashing in liquid were acquired using the WormLab (MBF Bioscience). Video analysis using the built-in software was used to calculate the body size, crawl speed and thrash (swimming) rates. (B) Body lengths and (C) thrash rates of p.Gly1345Ter variant animals. Each dot represents one animal. [n= 61-83 animals for (B). n=44-91 for (C)]. (D) Body lengths, (E) crawl speeds, and (F) thrash rates of p.Asp1239Asn and p.Arg350Gly variant animals. n=65-78 animals for (D) and (E). n=75-87 animals for (F). (G) UPR activation as measured by HSP-4::GFP expression. Each dot represents the average GFP intensity per worm in a well containing about 10 worms. n=25-27 wells for (G). In (B)-(F), error bars show median plus interquartile range. In (G), error bars show mean plus 95% confidence interval.

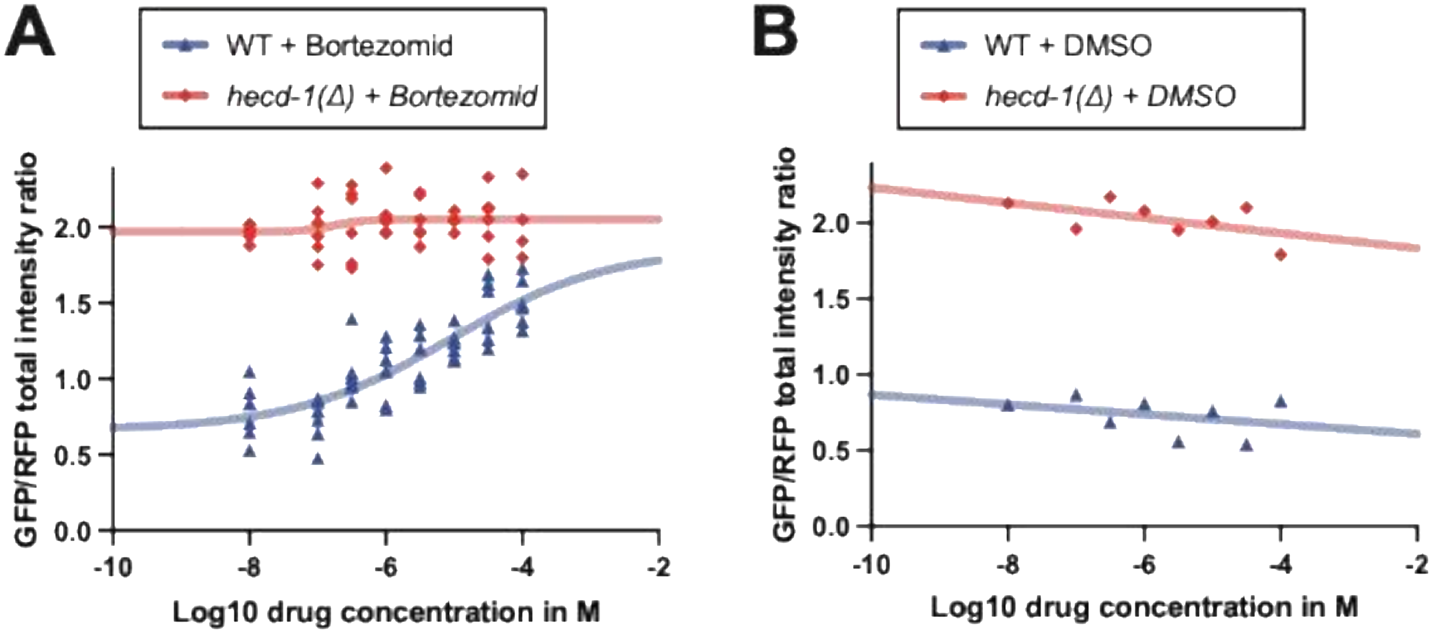

Figure S3. Bortezomib inhibition of proteasome activity in *C. elegans* as measured by Ub::GFP degradation

(A) Treatment of wild type animals expressing Ub::GFP with proteasomal inhibitor bortezomib (dissolved in DMSO) resulted in a dose-dependent increase in Ub::GFP accumulation. *hecd-1* null *(Δ)* animals have elevated Ub::GFP (see Figure 5) which was not altered by bortezomib treatment. Each dot represents a well containing about 10 animals. (n=4-6 wells) (B) Control experiment showing DMSO treatment does not change Ub::GFP levels in wild type or *hecd-1* null *(Δ)* animals. Each dot represents a well containing about 10 animal (n=1 well).

STRAIN NAME	GENOTYPE	DESCRIPTION AND REFERENCES
VC2010	wild type	WT[1]
UDN100517	*hecd-1(udn257)*	p.Arg350Gly #1 (variant, line #1)
UDN100518	*hecd-1(udn258)*	p.Arg350Gly #2 (variant, line #2)
UDN100521	*hecd-1(udn261)*	p.Arg350Arg #2 (control, line #2)
UDN100512	*hecd-1(udn252)*	p.Asp1239Asn #1 (variant, line #1)
UDN100513	*hecd-1(udn253)*	p.Asp1239Asn #2 (variant, line #2)
UDN100516	*hecd-1(udn256)*	p.Asp1239Asp #2 (control, line #2)
UDN100545	*hecd-1(udn262)*	p.Gly1345Ter #1 (variant, line #1)
UDN100540	*hecd-1(udn263)*	p.Gly1345Ter #2 (variant, line #2)
UDN100546	*hecd-1(udn265)*	p.Gly1345Gly #1 (control, line #1)
UDN100522	*hecd-1(ok1437)*	Deletion (Δ) allele. Carries a 1239bp deletion in *hecd-1* gene and results in a truncated HECD-1 protein losing its C-terminal half, from residue 1153 to the end. Outcrossed from strain RB1319.
UDN100523	*odIs77 [col−19p∷UbG76V-GFP, col−19p∷mRFP]*	Ub::GFP reporter alone
UDN100529	*hecd-1(udn257); odIs77*	p.Arg350Gly #1 with Ub::GFP reporter
UDN100530	*hecd-1(udn258); odIs77*	p.Arg350Gly #2 with Ub::GFP reporter
UDN100531	*hecd-1(udn261); odIs77*	p.Arg350Arg #2 with Ub::GFP reporter
UDN100526	*hecd-1(udn252); odIs77*	p.Asp1239Asn #1 with Ub::GFP reporter
UDN100527	*hecd-1(udn253); odIs77*	p.Asp1239Asn #2 with Ub::GFP reporter
UDN100528	*hecd-1(udn256); odIs77*	p.Asp1239Asp #2 with Ub::GFP reporter
UDN100551	*hecd-1(udn262); odIs77*	p.Gly1345Ter #1 with Ub::GFP reporter
UDN100561	*hecd-1(udn263); odIs77*	p.Gly1345Ter #2 with Ub::GFP reporter
UDN100552	*hecd-1(udn265); odIs77*	p.Gly1345Gly #1 with Ub::GFP reporter

UDN100525	*hecd-1(ok1437); odIs77*	Deletion (Δ) allele with Ub::GFP reporter
UDN100498	*zcIs4 [hsp-4p::GFP] V*	*HSP-4P::GFP* marker alone
UDN100584	*hecd-1(udn257); zcIs4*	p.Arg350Gly #1 with *hsp-4p::GFP* marker
UDN100583	*hecd-1(udn252); zcIs4*	p.Asp1239Asn #1 with *hsp-4p::GFP* marker
UDN100585	*HECD-1(UDN262); ZCIS4*	p.Gly1345Ter #1 with *hsp-4p::GFP* marker

Table S1. *C. elegans* strains
Description of *C. elegans* strains used in this study. *odls77* allele carries a multicopy insertion of the Ub::GRP reporter and mRFP under the *col-19* promoter. This reporter was used to assay protein degradation by the ubiquitin-proteosome system (UPS). *zcls4* allele carries a multicopy insertion of the plasmid, *hsp-4p*::GRP, which is activated during the unfolded protein response.

hecd-1(p.Gly1345) gRNA	CAAAACGGAAAGAACTCTTC
p.Gly1345Ter repair template	CAACCGCATTCAGATTCTTCAGAATTGCTCAAAACGG AAAGAACTCTagttaaCAAACACATTACTTGTCTTGTTCC GGATTCGAGATTTACGGAGACAT
p.Gly1345Gly repair template	CAACCGCATTCAGATTCTTCAGAATTGCTCAAAACGG AAAGAACTCTagtGGTCAAACACATTACTTGTCTTGTTC CGGATTCGAGATTTACGGAGACAT
hecd-1(p.Asp1239) gRNA	TCACGTGACAGAAGATCTTC
p.Asp1239Asn repair template	GTTAAAATCACTTGTTCGGATACTCGCCAACCATTCG GTAAACCTGAgaATtTgCTGTCACGTGATCAAAATCCTA TCAATTGTCATACTTCCGATGATA
p.Asn1239Asn repair template	GTTAAAATCACTTGTTCGGATACTCGCCAACCATTCG GTAAACCTGAgGATtTgCTGTCACGTGATCAAAATCCTA TCAATTGTCATACTTCCGATGATA
hecd-1(p.Arg350) gRNA	AGTCAAATCTTTCTGTCTGA
p.Arg350Gly repair template	TGCAGCAGGTAGTGGAGCTGAACGTGTTCATAGACA ATTGATTGACGCaATtgGACAGAAAGATTTGACTGCTTT GGTGGATGCCATCGAAAGTGGACAG
p.Arg350Arg repair template	TGCAGCAGGTAGTGGAGCTGAACGTGTTCATAGACA ATTGATTGACGCaATtAGACAGAAAGATTTGACTGCTT TGGTGGATGCCATCGAAAGTGGACAG

Table S2. CRISPR/Cas9 genome editing reagents for hecd-1

gRNA and repair template sequences used for *C. elegans* genome editing are shown. Single strand repair templates were used to introduce the variants and control edits. Lower-case letters in the repair templates denote changes compared to the wild-type sequence.

ID	Genomic location (hg38)	mRNA	Protein	Domain	Inheritance evidence	Inheritance	gnomAD MAF	Effect on protein	Splice AI	REVEL score	CADD phred	Poly phen 2
1	14:31205797_G>A	NM_015382.4:c.140C>T	NP_056197.3:p.(Thr47Ile)	ARM	NA	unknown	0			0.412	33	D
2	14:31173700_A>G	NM_015382.4:c.710T>C	NP_056197.3:p.(Leu237Ser)	ARM	PS2	*de novo*	0			0.514	27.4	D
3	14:31173201_G>C	NM_015382.4:c.1111C>G	NP_056197.3:p.(Arg371Gly)	AR	PS2	*de novo*	0			0.392	26.3	D
4	14:31150072_G>C	NM_015382.4:c.2082C>G	NP_056197.3:p.(Phe694Leu)	NA	PS2	*de novo*	0			0.379	24.6	D
5	14:31135104_C>T	NM_015382.4:c.3346G>A	NP_056197.3:p.(Asp1116Asn)	SUN	PS2	*de novo*	0			0.407	27.7	D
6	14:31135100_A>G	NM_015382.4:c.3350T>C	NP_056197.3:p.(Ile1117Thr)	SUN	PS2	*de novo*	0			0.555	27.1	D
7	14:31133545_A>T	NM_015382.4:c.3709T>A	NP_056197.3:p.(Tyr1237Asn)	SUN	PS2	*de novo*	0			0.68	29.5	D
8	14:31129377_G>A	NM_015382.4:c.3994C>T	NP_056197.3:p.(Leu1332Phe)	MIB	PS2	*de novo*	0			0.624	28	D
9	14:31106737_G>A	NM_015382.4:c.7135C>T	NP_056197.3:p.(Leu2379Phe)	HECT	PS2	*de novo*	0			0.203	24.5	D
10	14:31169471_AGAG>A	NM_015382.4:c.1333-6_1333-4del	splice variant	NA	PS2	*de novo*	0	BP4	0.01	NA	NA	NA
11	14:31148966_C>CT	NM_015382.4:c.2349dup	NP_056197.3:p.(Asp784ArgfsTer8)	NA	NA	unknown	0	PVS1		NA	NA	NA
12	14:31133584_C>A	NM_015382.4:c.3670G>T	NP_056197.3:p.(Gly1224Ter)	SUN	PM6	*de novo*	0	PVS1		NA	38	NA
13	14:31103032_T>TA	NM_015382.4:c.7433dup	NP_056197.3:p.(Leu2478PhefsTer7)	HECT	NA	unknown	0	PVS1		NA	NA	NA
14	14:31107648_AG>A	NM_015382.4:c.6536delC	NP_056197.3:p.(Ala2179ValfsTer35)	HECT	PM3	compound het	0	PVS1		NA	NA	NA
14	14:31175038_T>C	NM_015382.4:c.476A>G	NP_056197.3:p.(His159Arg)	ARM	PM3	compound het	0			0.735	26.1	P

Table S3: *HECTD1* Variant information and functional prediction scores

The variant classifications in “Inherit evidence” and “Effect on protein” columns are based on the ACMG criteria[2]. Truncating variants are classified as PVS1 while the splice variant with benign predicted Splie AI score is classified as BP4. Splice AI[3] , CADD phred[4], REVEL[5] and Polyphen2[6] are prediction programs for pathogenicity of variants. Domain abbreviations: SUN=Sad1 and UNC-84; ARM=armadillo-like helical; AR=ankyrin repeat; HECT=homologous to E6AP C-terminus; MIB= MIB/HERC2. MAF=minor allele frequency from gnomAD, het=heterozygous, D=damaging, P=possibly damaging, NA=Not applicable.

PMID	Phenotype
35654974	Autism
30675382	Autism
37543562	Autism
35982159	Autism
27525107	Autism
28263302	Autism
26582266	Autism
23260136	Autism
25363760	Autism
26749308	Autism
24650168	Autism
24501278	Autism
25418537	Autism
26352270	Autism
28965761	Autism
25961944	Autism
23160955	Autism
30504930	Autism
30545852	Autism
26785492	Congenital Heart Disease
27479907	Congenital Heart Disease
23665959	Congenital Heart Disease
33057194	Developmental Disorder
23934111	Epilepsy
23647072	Epilepsy
26795593	Epilepsy
22365152	Epilepsy

23086397	Epilepsy
27479843	Intellectual Disability
23033978	Intellectual Disability
27334371	Intellectual Disability
37158973	Neurodevelopmental Disorder
38114583	Neurodevelopmental Disorder

Table S4. Source and phenotype of published cohort data used in DenovolyzeR and chimpanzee-human *de novo* variant (DNV) analysis

Phenotype	Unique Number of Individuals
Autism	19,391
Congenital Heart Disease	2,072
Developmental Disorder	31,058
Epilepsy	532
Intellectual Disability	190
Neurodevelopmental Disorder	62
Total	53,305

Table S5. Phenotypes and counts of unique individuals in published cohorts after removing overlaps between studies

Chrom	Pos (b38)	Ref	Alt	Sample	PMID	Phenotype	Consequence	HGVSc	HGVSp
chr14	31106802	G	C	1-02563	26785492	congenital_heart_disease	missense	NM_015382.4:c.7070C>G	NP_056197.3:p.(Ala2357Gly)
chr14	31107685	T	C	DDD13k.02131	33057194	developmentalDisorder	missense	NM_015382.4:c.6500A>G	NP_056197.3:p.(Glu2167Gly)
chr14	31109507	G	A	DDD13k.04533	33057194	developmentalDisorder	stop_gained	NM_015382.4:c.6370C>T	NP_056197.3:p.(Arg2124Ter)
chr14	31112423	C	T	rumc_patient_476	33057194	developmentalDisorder	missense	NM_015382.4:c.6271G>A	NP_056197.3:p.(Ala2091Thr)
chr14	31113390	G	A	3456	33057194	developmentalDisorder	missense	NM_015382.4:c.5951C>T	NP_056197.3:p.(Pro1984Leu)
chr14	31121394	T	C	SP0130244	35982159	autism	missense	NM_015382.4:c.5227A>G	NP_056197.3:p.(Ile1743Val)
chr14	31128819	C	A	SP0123550	35982159	autism	missense	NM_015382.4:c.4552G>T	NP_056197.3:p.(Val1518Leu)
chr14	31133321	A	C	27672	33057194	developmentalDisorder	missense	NM_015382.4:c.3839T>G	NP_056197.3:p.(Val1280Gly)
chr14	31136577	T	C	71821	33057194	developmentalDisorder	missense	NM_015382.4:c.3068A>G	NP_056197.3:p.(Glu1023Gly)
chr14	31144125	G	GT	13711.p1	25363768	autism	frameshift	NM_015382.4:c.2764dup	NP_056197.3:p.(Thr922AsnfsTer6)
chr14	31144245	C	G	rumc_patient_872	33057194	developmentalDisorder	missense	NM_015382.4:c.2644G>C	NP_056197.3:p.(Glu882Gln)
chr14	31173239	G	A	5352	33057194	developmentalDisorder	missense	NM_015382.4:c.1073C>T	NP_056197.3:p.(Ser358Phe)
chr14	31173538	G	A	117451	33057194	developmentalDisorder	missense	NM_015382.4:c.872C>T	NP_056197.3:p.(Pro291Leu)
chr14	31173700	A	G	53609	33057194	developmentalDisorder	missense	NM_015382.4:c.710T>C	NP_056197.3:p.(Leu237Ser)

Table S6. Missense and likely gene disrupting *HECTD1 de novo* variants in published cohorts

Gene	Class	Observed	Expected	Enrichment	P-value
HECTD1	mis	12	7.5	1.6	0.0785
HECTD1	lof	2	1	2.03	0.259
HECTD1	prot	14	8.5	1.65	0.0504

Table S7. Enrichment of *HECTD1 de novo* variants in published cohorts derived from DenovolyzeR analysis

GeneName	Nonsynon	LoF	Allp	Nsp	Truncp
HECTD1	12	2	4.02E-06	2.47E-05	0.05218636

Table S8. Enrichment of *HECTD1 de novo* variants in published cohorts derived from Chimpanzee-Human De Novo Variant (DNV) test

REFERENCES

1. Yoshimura, J., Ichikawa, K., Shoura, M.J., Artiles, K.L., Gabdank, I., Wahba, L., Smith, C.L., Edgley, M.L., Rougvie, A.E., Fire, A.Z., et al. (2019). Recompleting the Caenorhabditis elegans genome. Genome Res *29*, 1009-1022. 10.1101/gr.244830.118.
2. Richards, S., Aziz, N., Bale, S., Bick, D., Das, S., Gastier-Foster, J., Grody, W.W., Hegde, M., Lyon, E., Spector, E., et al. (2015). Standards and guidelines for the interpretation of sequence variants: a joint consensus recommendation of the American College of Medical Genetics and Genomics and the Association for Molecular Pathology. Genet Med *17*, 405-424. 10.1038/gim.2015.30.
3. Jaganathan, K., Kyriazopoulou Panagiotopoulou, S., McRae, J.F., Darbandi, S.F., Knowles, D., Li, Y.I., Kosmicki, J.A., Arbelaez, J., Cui, W., Schwartz, G.B., et al. (2019). Predicting Splicing from Primary Sequence with Deep Learning. Cell *176*, 535-548 e524. 10.1016/j.cell.2018.12.015.
4. Kircher, M., Witten, D.M., Jain, P., O'Roak, B.J., Cooper, G.M., and Shendure, J. (2014). A general framework for estimating the relative pathogenicity of human genetic variants. Nat Genet *46*, 310-315. 10.1038/ng.2892.
5. Ioannidis, N.M., Rothstein, J.H., Pejaver, V., Middha, S., McDonnell, S.K., Baheti, S., Musolf, A., Li, Q., Holzinger, E., Karyadi, D., et al. (2016). REVEL: An Ensemble Method for Predicting the Pathogenicity of Rare Missense Variants. Am J Hum Genet *99*, 877-885. 10.1016/j.ajhg.2016.08.016.
6. Adzhubei, I.A., Schmidt, S., Peshkin, L., Ramensky, V.E., Gerasimova, A., Bork, P., Kondrashov, A.S., and Sunyaev, S.R. (2010). A method and server for predicting damaging missense mutations. Nat Methods *7*, 248-249. 10.1038/nmeth0410-248.
