# Supplementary material for: Sequence variants in HECTD1 result in a variable neurodevelopmental disorder
Source: Am J Hum Genet. 2025 Jan 28;112(3):537–53. doi: 10.1016/j.ajhg.2025.01.001 (PMC11947180; doi:10.1016/j.ajhg.2025.01.001)
Supplement: Document S2. Article plus supplemental information [file mmc2.pdf]

Sequence variants in *HECTD1* result in a variable neurodevelopmental disorder

Authors

Gazelle Zerafati-Jahromi, Elias Oxman,
Hieu D. Hoang, ..., Stephen C. Pak, Irene E. Zohn,
Christina A. Gurnett

Correspondence

izohn@childrensnational.org (I.E.Z.),
gurnettc@wustl.edu (C.A.G.)

Dysregulation of homologous to E6AP C-Terminus (HECT) E3 ubiquitin ligases is linked to cancer and structural birth defects. Zerafati-Jahromi et al. describe *HECTD1* variants in 14 individuals with neurodevelopmental disorders and enrichment in 53,305 published trios. Critical for brain development, *HECTD1* variants show both change-of-function and loss-of-function mechanisms.

Zerafati-Jahromi et al., 2025, The American Journal of Human Genetics *112*, 537–553
March 6, 2025

© 2025 The Authors. Published by Elsevier Inc. on behalf of American Society of Human Genetics.
https://doi.org/10.1016/j.ajhg.2025.01.001

Sequence variants in *HECTD1* result in a variable neurodevelopmental disorder

Gazelle Zerafati-Jahromi,[1,29] Elias Oxman,[5,29] Hieu D. Hoang,[4,29] Wu-Lin Charng,[1] Tanvitha Kotla,[4] Weimin Yuan,[4] Keito Ishibashi,[5] Sonia Sebaoui,[6] Kathryn Luedtke,[5] Bryce Winrow,[5] Rebecca D. Ganetzky,[7,8,9] Anna Ruiz,[10] Carmen Manso-Basúz,[10] Nino Spataro,[10] Peter Kannu,[11] Taryn Athey,[11] Christina Peroutka,[12] Caitlin Barnes,[12] Richard Sidlow,[13] George Anadiotis,[14] Kari Magnussen,[14] Irene Valenzuela,[15] Alejandro Moles-Fernandez,[15] Seth Berger,[16] Christina L. Grant,[16] Eric Vilain,[27] Gudny A. Arnadottir,[17] Patrick Sulem,[17] Telma S. Sulem,[17] Kari Stefansson,[17] Shavonne Massey,[18] Natalie Ginn,[18] Annapurna Poduri,[19,22] Alissa M. D'Gama,[21,22,23] Rozalia Valentine,[22] Sara K. Trowbridge,[19,20] Chaya N. Murali,[24] Rachel Franciskovich,[24] Yen Tran,[25] Bryn D. Webb,[26] Kim M. Keppler-Noreuil,[26] April L. Hall,[26] Bobbi McGivern,[28] Kristin G. Monaghan,[28] Maria J. Guillen Sacoto,[28] Dustin Baldridge,[4] Gary A. Silverman,[4] Sonika Dahiya,[2] Tychele N. Turner,[3] Tim Schedl,[3] Joshua G. Corbin,[6] Stephen C. Pak,[4,30,31] Irene E. Zohn,[5,30,31,*] and Christina A. Gurnett[1,30,31,*]

Summary

Dysregulation of genes encoding the homologous to E6AP C-terminus (HECT) E3 ubiquitin ligases has been linked to cancer and structural birth defects. One member of this family, the HECT-domain-containing protein 1 (*HECTD1*), mediates developmental pathways, including cell signaling, gene expression, and embryogenesis. Through GeneMatcher, we identified 14 unrelated individuals with 15 different variants in *HECTD1* (10 missense, 3 frameshift, 1 nonsense, and 1 splicing variant) with neurodevelopmental disorders (NDDs), including autism, attention-deficit/hyperactivity disorder, and epilepsy. Of these 15 *HECTD1* variants, 10 occurred *de novo*, 3 had unknown inheritance, and 2 were compound heterozygous. While all individuals in this cohort displayed NDDs, no genotype-phenotype correlation was apparent. Conditional knockout of *Hectd1* in the neural lineage in mice resulted in microcephaly, severe hippocampal malformations, and complete agenesis of the corpus callosum, supporting a role for *Hectd1* in embryonic brain development. Functional studies of select variants in *C. elegans* revealed dominant effects, including either change-of-function or loss-of-function/haploinsufficient mechanisms, which may explain phenotypic heterogeneity. Significant enrichment of *de novo* variants in *HECTD1* was also shown in an independent cohort of 53,305 published trios with NDDs or congenital heart disease. Thus, our clinical and functional data support a critical requirement of *HECTD1* for human brain development.

Introduction

Many neurodevelopmental disorders (NDDs) result from genetic differences in the ubiquitin-proteasome system (UPS).[1] Ubiquitylation is critical for almost all fundamental cellular processes by targeting substrate proteins to the proteasome for degradation or to modify their function by altering intracellular localization, activity, or protein interactions.[2] E3 ubiquitin ligases provide selectivity to the ubiquitin cascade by imparting substrate specificity

[1]Department of Neurology, Washington University in St. Louis, St. Louis, MO, USA; [2]Department of Pathology, Washington University in St. Louis, St. Louis, MO, USA; [3]Department of Genetics, Washington University in St. Louis, St. Louis, MO, USA; [4]Department of Pediatrics, Washington University in St. Louis, St. Louis, MO, USA; [5]Center for Genetic Medicine Research, Children's National Hospital, Washington, DC, USA; [6]Center for Neuroscience Research, Children's National Hospital, Washington, DC, USA; [7]Mitochondrial Medicine Frontier Program, Division of Human Genetics, Children's Hospital of Philadelphia, Philadelphia, PA, USA; [8]Department of Pediatrics, University of Pennsylvania Perelman School of Medicine, Philadelphia, PA, USA; [9]Center for Computational Genomics Medicine, Children's Hospital of Philadelphia, Philadelphia, PA, USA; [10]Center for Genomic Medicine, Parc Taulí Hospital University, Parc Taulí Institute of Research and Innovation (I3PT-CERCA), Autonomous University of Barcelona, Sabadell, Spain; [11]Department of Medical Genetics, Alberta Health Services, Edmonton, AB, Canada; [12]Department of Pediatrics, University of Virginia, Charlottesville, VA, USA; [13]Department of Medical Genetics and Metabolism, Valley Children's Hospital, Madera, CA, USA; [14]Department of Genetics and Metabolism, Randall Children's Hospital at Legacy Emanuel, Portland, OR, USA; [15]Department of Clinical and Molecular Genetics, University Hospital Vall d'Hebron and Medicine Genetics Group, Valle Hebron Research Institute, Barcelona, Spain; [16]Rare Disease Institute, Children's National Hospital, Washington, DC, USA; [17]deCODE Genetics/Amgen Inc., Reykjavik, Iceland; [18]Division of Neurology, Children's Hospital of Philadelphia, Philadelphia, PA, USA; [19]Department of Neurology, Boston Children's Hospital, Boston, MA, USA; [20]Department of Neurology, Harvard Medical School, Boston, MA, USA; [21]Division of Newborn Medicine, Boston Children's Hospital, Boston, MA, USA; [22]Epilepsy Genetics Program, Department of Neurology, Boston Children's Hospital, Boston, MA, USA; [23]Department of Pediatrics, Harvard Medical School, Boston, MA, USA; [24]Department of Molecular and Human Genetics, Baylor College of Medicine, Houston, TX, USA; [25]Department of Neurology, Baylor College of Medicine, Houston, TX, USA; [26]Department of Pediatrics, University of Wisconsin School of Medicine and Public Health, Madison, WI, USA; [27]Institute for Clinical and Translational Science, University of California, Irvine, Irvine, CA, USA; [28]GeneDx, Gaithersburg, MD, USA
[29]These authors contributed equally
[30]These authors contributed equally
[31]Senior author
*Correspondence: izohn@childrensnational.org (I.E.Z.), gurnettc@wustl.edu (C.A.G.)
https://doi.org/10.1016/j.ajhg.2025.01.001.
© 2025 The Authors. Published by Elsevier Inc. on behalf of American Society of Human Genetics.
This is an open access article under the CC BY license (http://creativecommons.org/licenses/by/4.0/).

and comprise three subgroups: RING (really interesting new gene), HECT (homologous to E6AP C terminus), and RBR (RING between RING). HECT domain ligases have a C-terminal HECT domain E3, which interacts with the E2 ubiquitin-conjugating enzyme and accepts and transfers ubiquitin to substrates.[3] *UBE3A* (MIM: 601623), responsible for Angelman syndrome (MIM: 105830), was the first HECT domain E3 ubiquitin ligase associated with an NDD.[4,5] Genes encoding ubiquitin ligases, deubiquitylating enzymes, and proteasome subunits now comprise a significant proportion of known NDD genes.[1,6]

HECTD1 (MIM: 618649) encodes a large protein (2,610 amino acids) with a C-terminal HECT domain and multiple protein-protein interaction domains. The protein interaction domains include two armadillo repeat domains, ankyrin-repeat domain, SUN (SAD1/UNC) domain, MIB (MIB/HERC2) domain, and basic tilted helix bundle (BTHB) domain. Studies in mouse models with loss-of-function mutations in *Hectd1* demonstrate a requirement for this gene in neural tube, hematopoietic stem cell, heart, and placental development.[7–12] Moreover, sequence variants in *HECTD1* have been reported in human neural tube defects (NTDs) and a single congenital heart defect (CHD) case, as well as in large autism and NDD cohorts.[13–16] A machine-learning model that considered gene expression patterns in the brain, network features, and constraint scores demonstrated that *HECTD1* was among the top ten candidates for autism genes.[17] However, the role of *HECTD1* in brain development and the etiology of human NDDs remains largely unknown.

Here, we describe a cohort of 14 individuals with 15 variants of uncertain significance in the *HECTD1* gene, including 10 missense, 3 frameshift, 1 nonsense, and 1 splicing variant associated with a spectrum of NDD phenotypes. These phenotypes, observed in 10 individuals with *de novo* variants, include autism, attention deficit/hyperactivity disorder (ADHD [MIM: 143465]), and epilepsy. A role for *HECTD1* variants in human neurobehavioral phenotypes is further supported by a significant enrichment of *HECTD1 de novo* variants in an independent cohort of 53,305 published trio sequencing cases involving probands diagnosed with either NDDs or congenital heart disease, brain abnormalities in *Hectd1* mutant mice, and variable effects of select missense variants in *C. elegans* models.

Subjects and methods

Subject recruitment and sequencing

The index individual was identified through a research sequencing study at Washington University in St. Louis and confirmed by clinical sequencing at GeneDx. The other 13 individuals were identified through GeneMatcher.[18] The initial GeneMatcher entry occurred on November 28, 2016. This study was conducted with informed consent of research participants under the Washington University School of Medicine Institutional Review Board (IRB) or the IRB affiliated with each participating center. The clinician for each included individual obtained consent for publication under their own site's protocols. Genome and exome-wide sequencing was performed in clinical diagnostic or research laboratories at each participating center using standard sequencing and analysis methods. The *de novo* inheritance of variants was confirmed with a trio design whenever parents were available for testing. Clinical data were gathered by means of retrospective chart review using a standardized survey completed by each clinician. Histological images of the resected cortex were of samples taken during epilepsy surgery in individual 2.

In silico analysis

Sequence data were acquired from National Center for Biotechnology Information (NCBI), and references were from GenBank using the GRCh28p.13 primary assembly. NCBI Reference Sequence accession RefSeq: NM_015382.4 was used to define the cDNA and amino acid changes. These variants were analyzed *in silico* using multiple pathogenicity scores, including Combined Annotation-Dependent Depletion (CADD),[19] REVEL,[20] and PolyPhen2.[21] Variants were also annotated with Genome Aggregation Database (gnomAD) minor allele frequencies.[22]

The predicted *HECTD1* three-dimensional (3D) structure (AF-Q9ULT8-F1) was obtained from the AlphaFold Protein Structure database.[23,24] Annotation of the variant locations and motifs was performed using iCn3D (https://www.ncbi.nlm.nih.gov/Structure/icn3d/full.html). We acknowledge that AlphaFold may represent a 3D model unconfirmed for purposes of functional analysis; the figure shown depicts a basic representation of the spatial and secondary structure orientation of individual missense variants. Sequence alignment focused on the amino acid sequences around missense variants using a previously conducted blastp and MUSCLE (multiple sequence comparison by log-expectation) analysis.[14] Multiple sequence alignment was visualized using SnapGene Software (from Dotmatics; available at Snapgene.com).

Analysis of mouse models

All mouse work was performed in accordance with protocols approved by the Institutional Animal Care and Use Committee of the Children's National Research Institute. The $Hectd1^{opm}$ and $Sox1^{tm1(cre)Take}$ (*Sox1cre*) lines were previously described.[7,25] The $Hectd1^{flox}$ line was generated by crossing the previously described $Hectd1^{tm1a(EUCOMM)Hmgu}$ line to a FLPo deleter strain (*Gt*(*ROSA*)$26Sor^{tm2(FLP*)Sor}$; JAX stock #012930).[10,26] All mice were maintained on a congenic 129/SvJ background following 5–10 generations of backcrossing. On postnatal days 27–37 (P27–P37), mice were weighed, anesthetized, and transcardially perfused with 4% paraformaldehyde in PBS. Brains were removed, weighed, post-fixed overnight, cryoprotected in 30% sucrose in PBS, embedded in OCT, cryosectioned at a thickness of 30 μm, mounted on microscope slides, and allowed to dry. Nissl staining with cresyl violet was followed by dehydration in an alcohol gradient, xylene clearance, and coverslipping of the slides. Brain weights were taken from eight cKO and eight wild-type mice across 11 litters. Of these, Nissl staining was done on seven cKO and three wild-type brains. Images were acquired with a Leica DM6B microscope at 10× and processed in ImageJ and Adobe Photoshop, and figures were assembled in Microsoft PowerPoint. Images of dissected brains were acquired with an iPhone 14. Statistical analysis of brain and body weights was performed in GraphPad Prism version 10.2.3, and significance was determined by Student's t test. The chi-squared test was used to determine the significance of

morphological phenotypes in Nissl-stained brains using an online calculator (http://physics.csbsju.edu/stats/).

C. elegans strains and culture conditions

C. elegans were cultured on nematode growth media (NGM) plates seeded with *E. coli* strain OP50 as previously described.[27] All experiments were performed using homozygous animals at 20°C except when indicated that heterozygotes were used. Heterozygotes were generated by crossing homozygous (control or variant) females with wild-type males. Females were generated by treating animals with *fem-1* RNAi to abolish spermatogenesis as previously described.[28] VC2010 (wild-type parental strain) and deletion (Δ) allele (*ok1437*), which carries a 1,239 bp deletion in *hecd-1* gene and results in a truncated HECD-1 protein losing its C-terminal half from residue 1,153 to the end, were obtained from the *Caenorhabditis* Genetics Center (CGC; www.cgc.umn.edu). All strains were backcrossed a minimum of two times with the wild-type strain (VC2010) prior to phenotyping. A complete list of strains used in this study and their genotypes are shown in Table S1.

C. elegans genome editing using CRISPR-Cas9

Cas9 protein, guide RNAs, and single-stranded 100-base DNA oligos were microinjected into gonads of young adult hermaphrodites to generate *hecd-1* human variant and control edit strains. Cas9 protein (S.p. HiFi Cas9 Nuclease V3), tracRNA, crRNAs, and DNA repair template oligos were purchased from IDT (www.idtdna.com). The sequences of crRNA specific to the target loci and the corresponding repair templates are listed in Table S2. Of the multiple isoforms of *hecd-1* reported on the WormBase website (http://www.wormbase.org), isoform d was used, as it is the most abundant.[29] Independently isolated variant or control strains were labeled with # and a number (e.g., Arg350Gly #1 and Arg350Gly #2). All strains were outcrossed two times with the wild type (VC2010 strain) to reduce genetic background differences. The entire *hecd-1* gene was sequenced in all edited strains to ensure that no off-target changes were inadvertently introduced during editing.

Measurement of *C. elegans* crawl speed, thrash rate, and body length

WormLab (MBF Bioscience) was used to acquire videos of worms (i.e., 24 h post L4 larval stage) crawling on 60-mm NGM plates seeded with *E. coli* and thrashing in PBS liquid as previously described.[28,30] WormLab software (version 2019.1.1) was used to calculate crawl speed, head turn count, and body length. Thrash rate is defined as the number of head turn cycles per minute (where 1 turn cycle is equal to 2 turn counts).

Quantification of *C. elegans* ubiquitin-GFP accumulation

Ubiquitin green fluorescent protein (GFP) marker strain (allele *odIs77*) was characterized by Liu et al.[31] The strain expresses UB^{G76V}-GFP (abbreviated as UbGFP) and mRFP (red fluorescent protein) under the control of the *col-19p* promoter, which drives expression in the hypoderm. HECD-1 polyubiquitylates the UbGFP and therefore marks it for degradation. mRFP was used to control for gene expression. UbGFP levels were quantified in 2-day-old adult animals (i.e., 48 h post L4 larval stage). Animals were homozygous or heterozygous for *hecd-1* (controls or variants) and homozygous for the UbGFP marker. UbGFP and mRFP were quantified using the CellInsight CX7 high-content imager (Thermo Scientific). Approximately ten worms were placed into a well of a 384-well plate containing PBS buffer + 0.05% pluronic acid, a surfactant to prevent worms from sticking to the wall of the well. The worms were then anesthetized with levamisole (1.5 mg/mL) in the same buffer for 5 min before image acquisition. GFP and RFP thresholds were set at 25% maximum intensity of wild-type worms expressing the *odIs77* transgene. Total intensities from GFP and RFP channels were measured, and UbGFP accumulation was expressed as a ratio of GFP/RFP signal intensities.

Proteasomal inhibitor (bortezomib) treatment of *C. elegans* expressing Ub-GFP

Bortezomib eight-point dose-response studies were performed in 384-well plates. One-day-old adult animals were treated with 100 μM, 31.25 μM, 10 μM, 3.125 μM, 1 μM, 0.3125 μM, 0.1 μM, and 0.01 μM concentrations of bortezomib (Millipore Sigma, cat. #5.04314) or 0.2%, 0.0625%, 0.02%, 0.00625%, 0.002%, 0.000625%, 0.0002%, or 0.00002% DMSO (which corresponds to the DMSO concentration in the corresponding drug concentrations). Animals were exposed to the drug or DMSO for 18 h, anesthetized using levamisole, and imaged using the CellInsight CX-7 high-content imager as previously described.[32] Levels of UbGFP and mRFP were quantified and expressed as a ratio of GFP/mRFP.

Quantification of *C. elegans* unfolded protein response using HSP-4/GFP

The HSP-4/GFP reporter line (allele *zcIs4*) was used as a marker for endoplasmic reticulum (ER) stress and unfolded protein response (UPR) as previously described.[33] HSP-4/GFP was driven under *hsp-4* promoter and was expressed at low intensity under normal growing conditions. HSP-4/GFP expression was measured in 3-day-old adult animals. GFP total intensity was measured on the CellInsight CX7 high-content imager as described above. Data were expressed as GFP total intensity per animal.

Statistics and graphs

Data from at least three biological replicates from separate individual worms or worm populations were reported. Normality was determined by QQ plots, D'Agostino and Pearson tests, Anderson-Darling tests, Shapiro-Wilk tests, and Kolmogorov-Smirnov tests. Datasets with non-normal distribution were graphed as median + interquartile range. Kruskal-Wallis tests (non-parametric alternative of ANOVA) were used to determine statistical significance between different genotypes. For normally distributed datasets, equal variance was determined using Bartlett's tests. Normally distributed datasets were graphed as mean + 95% confidence interval. Of those, ordinary one-way ANOVA was used to determine difference between genotypes in equal-variance datasets. Brown-Forsythe and Welch one-way ANOVA tests were used to determine statistical significance between genotypes in unequal-variance datasets. All statistical tests and graphs were generated using GraphPad Prism version 10.2.3.

Results

Genetics findings

Our index individual (individual 2) presented for evaluation at 8 years of age with new-onset focal epilepsy. Clinical trio exome sequencing reported a *de novo* missense variant of uncertain significance in the candidate gene *HECTD1*. Through responses to GeneMatcher, we identified 13

Table 1. **Proband *HECTD1* variants**

Individual ID	Sequence variant (c.DNA)	Amino acid change	Inheritance	gnomAD MAF	Other variants
1	c.140C>T	p.Thr47Ile	unknown	0	–
2	c.710T>C	p.Leu237Ser	*de novo*	0	–
3	c.1111C>G	p.Arg371Gly	*de novo*	0	–
4	c.2082C>G	p.Phe694Leu	*de novo*	0	–
5	c.3346G>A	p.Asp1116Asn	*de novo*	0	*COL5A1* VUS
6	c.3350T>C	p.Ile1117Thr	*de novo*	0	–
7	c.3709T>A	p.Tyr1237Asn	*de novo*	0	–
8	c.3994C>T	p.Leu1332Phe	*de novo*	0	–
9	c.7135C>T	p.Leu2379Phe	*de novo*	0	*TTN* likely pathogenic
10	c.1333−6_1333−4del	Splice variant (in intron 8 from −4 to −6 of splice site)	*de novo*	0	–
11	c.2349dup	p.Asp784ArgfsTer8	unknown	0	*DLL1* likely pathogenic
12	c.3670G>T	p.Gly1224Ter	*de novo*	0	–
13	c.7433dup	p.Leu2478PhefsTer7	unknown	0	–
14	c.6536delC	p.Ala2179ValfsTer35	compound heterozygous	0	–
14	c.476A>G	p.His159Arg	compound heterozygous	0	–

RefSeq mRNA: NM_015382.4; RefSeq protein: NP_056197.3; VUS, variant of uncertain significance; MAF, minor allele frequency.

additional unrelated individuals with *HECTD1* variants (Table 1). The individuals' current ages range from 6 months to 28 years old. There were eight males and six females. From these 14 individuals, we identified 15 *HECTD1* variants, including compound heterozygous frameshift and missense variants in individual 14, both inherited from apparently healthy parents. Of the other 13 variants, ten were *de novo* and three were of unknown inheritance.

Five variants were predicted to result in a non-functional ubiquitin ligase due to truncation before the catalytic HECT domain. Among these were three frameshift variants (including one in individual 14 that occurred as a compound heterozygote with a missense variant), one nonsense, and one splicing variant (Table 1). None of these variants are in the last exon of the gene, and thus the variant-containing mRNAs are likely to undergo nonsense-mediated decay. Notably, as reported in the gnomAD database, *HECTD1* is highly intolerant to loss-of-function variation, with a loss-of-function-intolerant (pLI) score of 1 and a loss-of-function observed/expected upper bound fraction (LOEUF) score of 0.27, consistent with *HECTD1* being a likely haploinsufficient disease gene.[14,34,35]

Additional genetic findings that may be relevant to clinical phenotypes in this cohort include a paternally inherited variant of uncertain significance (VUS) in *COL5A1* (MIM: 120215) (Ehlers-Danlos syndrome, classic type [MIM: 130000]) in individual 5, which may explain his arachnodactyly, pectus carinatum, kyphosis, and aortic dilatation. A likely pathogenic frameshift variant in *DLL1* (MIM: 606582) of unknown inheritance was identified in individual 11, which was previously associated with neurodevelopmental phenotypes (MIM: 618709), including epilepsy and cortical malformations.[36] Individual 3 had severe mitochondrial complex I deficiency and moderate mitochondrial complex IV deficiency identified in liver electron transport chain enzymology, with no known genetic etiology, which likely explains his persistent transaminase elevations. No other individuals had reportable genetic findings that could explain the observed NDDs.

Human missense variants

The *HECTD1* gene (RefSeq: NP_056197.3; RefSeq: NM_015382.4) is highly constrained for missense variation, with a missense *Z* score of 6.42.[34,35] Ten missense variants occurred throughout multiple domains of the protein (Figure 1A) with no apparent three-dimensional clustering (Figure 1B). Only one missense variant (c.7135C>T [p.Leu2379Phe]) was identified in the HECT domain, which is essential for the ubiquitylation activity of HECTD1.[37] The majority of missense variants localize to protein interaction domains within HECTD1. Three variants (c.140C>T [p.Thr47Ile], c.476A>G [p.His159Arg], and c.710T>C [p.Leu237Ser]) are localized within the N-terminal ARM (armadillo-like helical) domain. The c.1111C>G (p.Arg371Gly) and c.2082C>G (p.Phe694Leu) variants flank the ankyrin repeat domain. Two adjacent amino acids within the SUN (Sad1 and UNC-84) domain, which may play a role in the localization of HECTD1 within the cell,[38] are altered by variants c.3346G>A

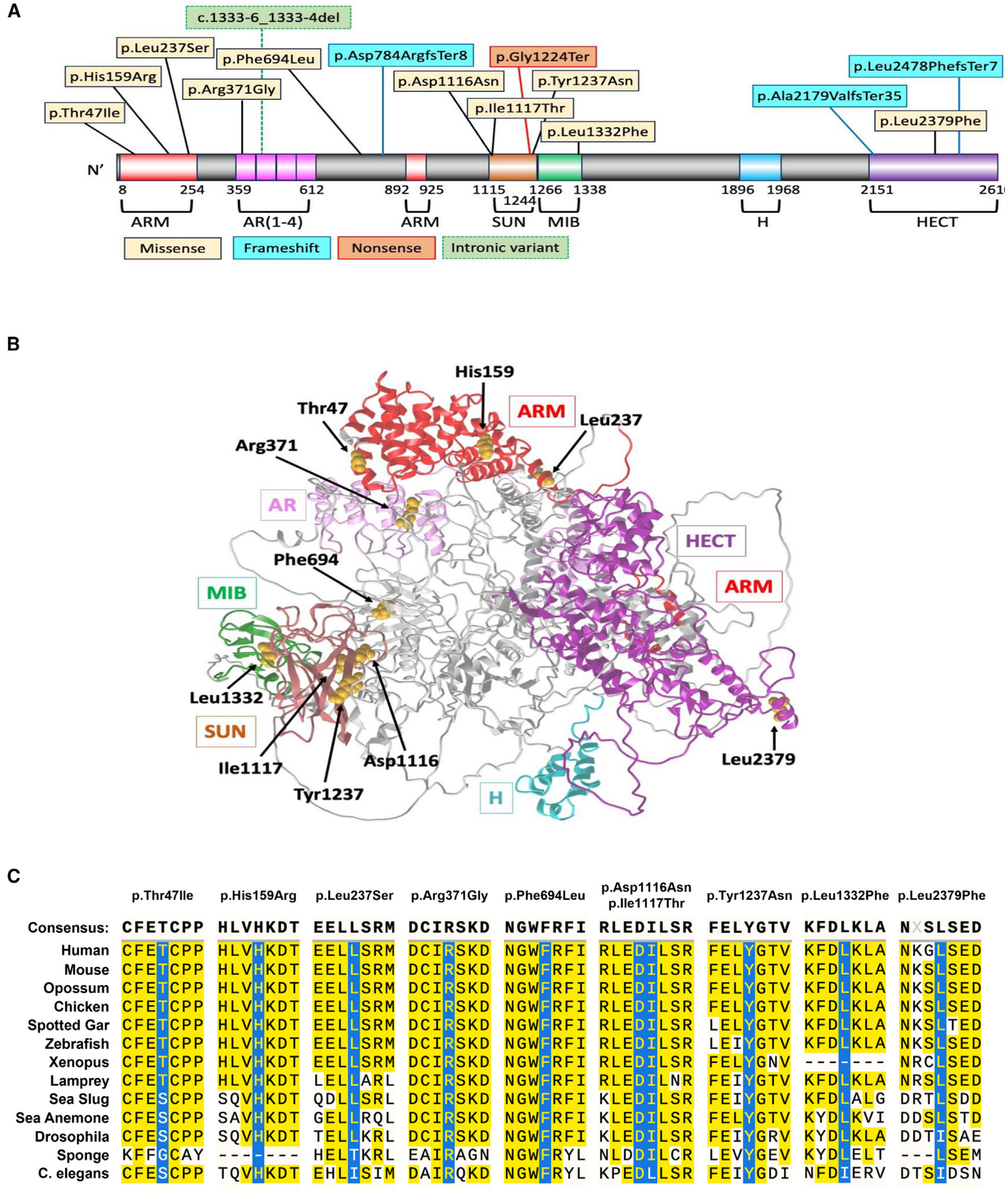

Figure 1. Location of *HECTD1* variants
(A) Variant subtypes and their locations within protein domains.
(B) Three-dimensional model showing location of variants near protein domains.
(C) Multiple sequence alignment for missense variants obtained through taxonomic filters on blastp with MUSCLE. Variant p.His159Arg is a compound heterozygote with p.Ala2179Vfs*35 in individual 14.

(p.Asp1116Asn) and c.3350T>C (p.Ile1117Thr), with a third variant c.3709T>A (p.Tyr1237Asn) in close physical proximity. The final missense variant, c.3994C>T (p.Leu1332Phe), is localized within the MIB-HERC2 (mind bomb and HECT and RLD domain containing E3 ubiquitin protein ligase 2) domain, which is present in

many ubiquitin ligases and may mediate interactions with substrates.[39] The localization of missense variants within or near key protein interaction domains suggests they may disrupt interaction with specific HECTD1-interacting proteins and substrates.

Numerous *in silico* models were used to predict the impact of the *HECTD1* missense variants. Multiple sequence alignment demonstrated a high degree of conservation between numerous vertebrate and invertebrate species (Figure 1C), and missense variants were predicted to be damaging by most algorithms (Table S3). All missense variants altered amino acids that are conserved among vertebrates. Variants p.Arg371Gly, p.Phe694Leu, p.Asp1116Asn, and p.Tyr1237Asn changed amino acids that are conserved across all species examined (Figure 1C). Moreover, residues not conserved in invertebrates were similar and had identical flanking residues.

Clinical phenotypes

Clinical descriptions for each of the 14 individuals are available in the supplemental case reports. Overall, abnormal growth parameter trends were not identified within this cohort of individuals with *HECTD1* variants, and no distinctive facial features were noted. Congenital anomalies were infrequent but diverse (Table 2). Oropharyngeal dysphagia, manifesting as a poor latch in infancy, aspiration, or failure to thrive, was common, resulting in gastric feeding tube placement in 5 of the 14 individuals (36%). Three of eight males had cryptorchidism (38%). Individual 9 had multiple congenital anomalies resulting in demise at age 6 months, including severe pulmonary vein stenosis, enlarged left kidney with cyst, duodenal atresia with tracheoesophageal fistula, biliary atresia with absent gallbladder, and imperforate anus. Individual 7 had tetralogy of Fallot and Pierre Robin sequence with facial clefting and developed postnatal microcephaly. Individual 4 had a laryngeal cleft. Individual 14, who had compound heterozygous variants consisting of both frameshift and missense variants that were inherited from each parent, had significant growth impairment affecting weight (−4.3 SD) and height (−5.5 SD), global developmental disabilities, and microcephaly. There were no consistent phenotypic differences between individuals with predicted loss-of-function variants and those with missense variants (Table 3).

Neurological and neurodevelopmental disability phenotypes

Eleven of the 14 individuals (79%) were reported to have varying degrees of NDDs (Table 2). Individual 9 was unable to be assessed due to death at age <6 months from multiple congenital anomalies, including severe pulmonary vein stenosis. Individual 6 had normal development at the last clinical update at 6 months of age. Individual 3 had global developmental delays that normalized by 3.5 years of age. NDDs primarily involved speech and language, with all but two persons of verbal age displaying impaired acquisition of language. The degree of language impairment was mild to moderate, with most speaking in single words and short phrases. Fine motor skills were impaired in 8/12 (67%) individuals. Gross motor skills were impaired in 4/12 (33%) individuals, and abnormal muscle tone was noted in 6/13 (46%).

Behavioral and psychiatric phenotypes

Psychiatric outcomes were reported for 11 individuals who were older than 1 year. Of these, 10 (91%) carried one or more diagnoses of psychiatric or behavioral conditions. In addition to the developmental delays described above, nearly half carried a diagnosis of autism spectrum disorder (5/11, 45%). Seven individuals (7/11, 64%) were diagnosed with ADHD. Six individuals displayed behavioral issues (6/11, 55%), including aggression (2/9, 22%), auditory hallucinations (2/9, 22%), self-injurious behavior (2/9, 22%), and anxiety (4/9, 44%).

Epilepsy

Six individuals in our cohort (6/14, 43%) developed epilepsy and had abnormal electroencephalograms (EEGs). Individual 1 had seizures and an abnormal EEG with rare left frontal sharp waves. Brain MRI demonstrated a 3-mm non-specific focus of increased T2 signal of the anterior left temporal lobe. Individual 2 began having focal seizures with dyscognitive features at 8 years of age. EEG demonstrated focal epileptiform abnormalities and seizure onset in the occipital and temporal lobes. Brain MRI demonstrated focal cerebral calcifications, multiple progressive subcortical white matter T2/FLAIR hyperintense foci, cystic encephalomalacia, and volume loss of the occipital lobes (Figures 2A and 2B). Cortical resection for intractable epilepsy demonstrated International League Against Epilepsy (ILAE) Type IIb focal cortical dysplasia characterized by the presence of dysmorphic and balloon neurons (Figures 2C and 2D) that were further variably highlighted by glial fibrillary acidic protein (GFAP), synaptophysin, and neuronal nuclei (NeuN) immunostainings (Figures 2E–2G). Individual 3 developed temporal lobe seizures, which were recorded on EEG; however, the brain MRI was normal. Individual 6 developed neonatal seizures on day 3 of life, characterized by staring and slow blinking, behavioral arrest, and apnea followed by profound desaturation and whole-body cyanosis. Two seizures, lasting between 1.5 and 2.5 min, were recorded on EEG, which demonstrated evolving sharp waves in the occipital regions, with spread into the left temporal region. Brain MRI on day 5 of life was unremarkable, demonstrating a trace (1–2 mm) right posterior convexity subdural hematoma. Individual 8 had seizure onset at 15 months old, with EEG demonstrating both generalized and bi-occipital interictal activity. The brain MRI was unremarkable, and the individual was treated with a vagal nerve stimulator for intractable epilepsy. Individual 11 had clinical seizures with EEGs showing mild diffuse background slowing as well as

Table 2. Clinical phenotypes of individuals with *HECTD1* variants

	Individual													
	**1**	**2**	**3**	**4**	**5**	**6**	**7**	**8**	**9**	**10**	**11**	**12**	**13**	**14**
	**Missense**									**Likely gene disruptive**				
Amino acid change	p.Thr47Ile	p.Leu237Ser	p.Arg371Gly	p.Phe694Leu	p.Asp1116Asn	p.Ile1117Thr	p.Tyr1237Asn	p.Leu1332Phe	p.Leu2379Phe	splice var.	p.Asp784ArgfsTer8	p.Gly1224Ter	p.Leu2478PhefsTer7	p.Ala2179ValfsTer35, p.His159Arg
Sex	F	M	M	F	M	M	M	F	M	M	F	F	M	F
Age	10 years	28 years	5 years	7 years	18 years	6 months	9 months	9 years	6 months	17 years	5 years	19 years	10 years	5 years
**Development**														
Language	+	+	−	+	+	−	+	−	N/A	+	+	+	+	+
Fine motor	−	+	−	+	+	−	+	+	N/A	N/A	−	+	+	+
Gross motor	−	−	−	+	−	−	+	−	N/A	N/A	−	+	−	+
Intellectual disability	N/A	+	N/A	N/A	+	N/A	N/A	N/A	N/A	N/A	N/A	+	N/A	N/A
**Neurological and psychiatric phenotypes**														
Microcephaly	−	−	−	−	−	−	+	−	−	−	+	−	−	+
Epilepsy	+	+	+	−	−	+	−	+	−	N/A	+	−	−	−
Abnormal muscle tone	−	−	−	+	−	+	+	+	−	N/A	−	+	+	−
ADD/ADHD	N/A	+	−	N/A	+	N/A	N/A	+	N/A	+	−	+	+	+
ASD	+	−	−	+	−	N/A	N/A	−	N/A	+	+	−	+	−
Anxiety	N/A	−	−	−	−	N/A	N/A	+	N/A	N/A	+	+	+	−
Self-injurious behavior	N/A	−	−	+	−	N/A	N/A	−	N/A	N/A	−	+	−	−
Hallucinations	N/A	+	−	−	−	N/A	N/A	−	N/A	N/A	−	−	+	−
Aggressive behavior	N/A	+	−	+	−	N/A	N/A	−	N/A	N/A	−	−	−	−
Abnormal brain MRI	+	+	−	+	−	−	−	−	+	N/A	+	−	N/A	−
**Systemic**														
Dysphagia	−	−	−	+	+	+	+	−	−	N/A	−	−	−	−
Tube feeding	−	−	+	+	−	−	+	+	+	N/A	−	−	−	−

(*Continued on next page*)

Table 2. *Continued*

	Individual													
	1	2	3	4	5	6	7	8	9	10	11	12	13	14
	**Missense**									**Likely gene disruptive**				
Cryptorchidism	N/A	–	–	N/A	+	–	+	N/A	–	–	N/A	N/A	+	N/A
Congenital heart disease	–	–	–	–	–	–	+	–	+	–	–	–	–	–

RefSeq protein: NP_056197.3; N/A, data was not reported; ADD, attention deficit disorder; AHDH, attention deficit/hyperactivity disorder; ASD, autism spectrum disorder; MRI, magnetic resonance imaging.

left occipital epileptiform discharges and brain MRI reportedly demonstrating right hippocampal atrophy. However, this individual also has a likely pathogenic variant in *DLL1* that also is associated with epilepsy.[36] Individual 7 had abnormal movements in infancy but a normal EEG.

Brain abnormalities

In addition to the variable cortical abnormalities identified for three of the six individuals with epilepsy described above, brain abnormalities were also reported at the age of 5 years old in individual 4, who had a small pars intermedia pituitary cyst and mild diffuse thickening of the corpus callosum but was not reported to have seizures. Three individuals had microcephaly.

Hectd1 is required for normal hippocampal and corpus callosum morphogenesis in a mouse model

As the function of Hectd1 in the nervous system is currently unknown, we assessed whether mutations in *Hectd1* in the mouse resulted in structural brain abnormalities. The $Hectd1^{opm}$ allele was generated in an ENU mutagenesis screen, which introduced a stop codon at amino acid 144 (p.Leu144Ter), resulting in a null allele.[7,8] Homozygous *Hectd1* loss-of-function mutant mice die during mid-gestation due to placental and heart defects.[7–9,11,12] To investigate the requirement of *Hectd1* for early mouse brain development, we generated a conditional knockout model using *Sox1-cre*-mediated recombination of a floxed *Hectd1* allele. *Sox1-cre* drives recombination in the neural lineage beginning at the neural plate stage.[25,40] $Hectd1^{flox/flox}$ females were mated with $Hectd1^{opm/+}$;*Sox1-cre* males and the brains of litters examined. The brain mass of conditional knockout mice that survived to P30 was reduced by ~30% compared to their wild-type littermates ($n = 8$, $p < 0.0001$) (Figures 3A–3C). In contrast, the body mass of $Hectd1^{opm/flox}$;*Sox1-cre* mice at P30 was variable, with some conditional knockout pups weighing less than their littermate controls and others with similar weights (Figure 3D). Thus, microcephaly was constant and uncorrelated with body mass (Figure 3E).

Compared to wild-type brain (Figures 4A–4E), histological analysis of surviving cKO P30 brains revealed significant morphological phenotypes that did not correlate with body mass (Figures 4F–4J). Complete agenesis of the corpus callosum was fully penetrant in the conditional knockout model (Figures 4F′–4I′) and was accompanied by other midline white matter tract defects, including dysgenesis of the hippocampal commissure and dilation of the third ventricle. The conditional knockout mice also exhibited abnormal organization of the hippocampal CA3 region that was most prominently observed in caudal sections (Figure 4J′). Corpus callosal and hippocampal dysgenesis were fully penetrant and consistent across all cKO mice ($n = 7$) (chi-squared = 10.0, degrees of freedom = 1, probability = 0.002).

Table 3. **Comparison of clinical features of individuals with *HECTD1* missense and likely gene-disruptive variants**

Features (HPO code)	No. with missense variants affected	No. with likely gene-disruptive variants affected
**Development[a]**		
Speech and language development (HP:0000750)	5/8	5/5
Fine motor development (HP:0010862)	5/8	3/4
Gross motor development (HP:0002194)	2/8	2/4
Intellectual disability (HP:0001249)	2/2	1/1
**Psychiatric[a]**		
ADD/ADHD (HP:0007018)	3/4	4/5
ASD (HP:0000729)	2/6	3/5
Anxiety (HP:0000739)	1/5	3/4
Self-injurious behavior (HP:0100716)	1/5	1/4
Hallucinations (HP:0000738)	1/5	1/4
Aggressive behavior (HP:0000718)	2/5	0/4
**Neurological**		
Epilepsy (HP:0001250)	5/9	1/4
Abnormal muscle tone (HP:0003808)	4/9	2/4
Gastrointestinal	–	–
Dysphagia (HP:0002015)	4/9	0/4
Tube feeding (HP:0033454)	5/9	0/4
**Other**		
Cryptorchidism (HP:0000028) in males	2/6	1/2

HPO, human phenome ontology; ADD, attention deficit disorder; ADHD, attention deficit/hyperactivity disorder; ASD, autism spectrum disorder.
[a]Not assessed in all due to age; individuals 10 (splice variant) and 14 (compound heterozygous variants) are included in the second column.

Functional analysis of *HECTD1* variants in *C. elegans*

To provide support of the human variants being damaging to HECD-1 function and to determine whether the genetic mechanism is consistent with heterozygous inheritance, we performed functional studies in *C. elegans*. We modeled two missense variants, p.Arg371Gly (individual 3) and p.Asp1116Asn (individual 5), and one nonsense variant, p.Gly1224Ter (individual 12), in the *C. elegans hecd-1*, which is the ortholog of *HECTD1*. These three variants had conserved amino acids at the corresponding locations in HECD-1 (Figure 1C), allowing CRISPR-Cas9 genome editing to precisely introduce the variants into the corresponding residues of *hecd-1* to generate p.Arg350Gly, p.Asp1239Asn, and p.Gly1345Ter variant edits in *C. elegans* (Figure S1A). We also generated control edits, p.Arg350Arg, p.Asp1239Asp, and p.Gly1345Gly, that are wild type at the variant residue in *C. elegans* but carry the same synonymous changes introduced during CRISPR-Cas9 editing to prevent Cas9 recleavage following homology-directed repair (Figure S1B). The premature termination codon, p.Gly1345Ter in HECD-1, located in the middle of the gene, likely leads to nonsense-mediated mRNA decay. A *hecd-1* null mutant (Δ) (*ok1437*) was also obtained from the CGC for comparison.

We evaluated the variant effects on *C. elegans* development and locomotion by measuring crawl speed and body length using WormLab (MBF Bioscience) (Figure S2A).[28] The homozygous null mutant (Δ) and two independent homozygous nonsense p.Gly1345Ter variant strains (#1 and #2) displayed significantly decreased crawl speed (Figure 5A) and reduced body length (Figure S2B) compared to the wild type and p.Gly1345Gly controls. The null mutants and the p.Gly1345Ter variant animals also had significantly reduced thrashing (swimming) when placed in liquid (Figure S2C). The p.Gly1345Ter variant animals behaved similarly to the null mutant for the three phenotypes tested. No crawling, thrashing, or body length phenotypes were detected in the two missense variants, p.Arg350Gly and p.Asp1239Asn (Figures S2D–S2F).

To test whether the variants alter the known ubiquitin (Ub)-mediated protein degradative function of HECD-1,[31,41,42] we utilized a previously described reporter, Ub^{G76V}-GFP, for assaying polyubiquitylation and proteasomal degradation to measure the loss of *hecd-1* activity in *C. elegans*.[31] In this assay, worms expressed an engineered non-cleavable variant of ubiquitin where the C-terminal Gly76 residue of Ub is mutated and fused to GFP (hereafter

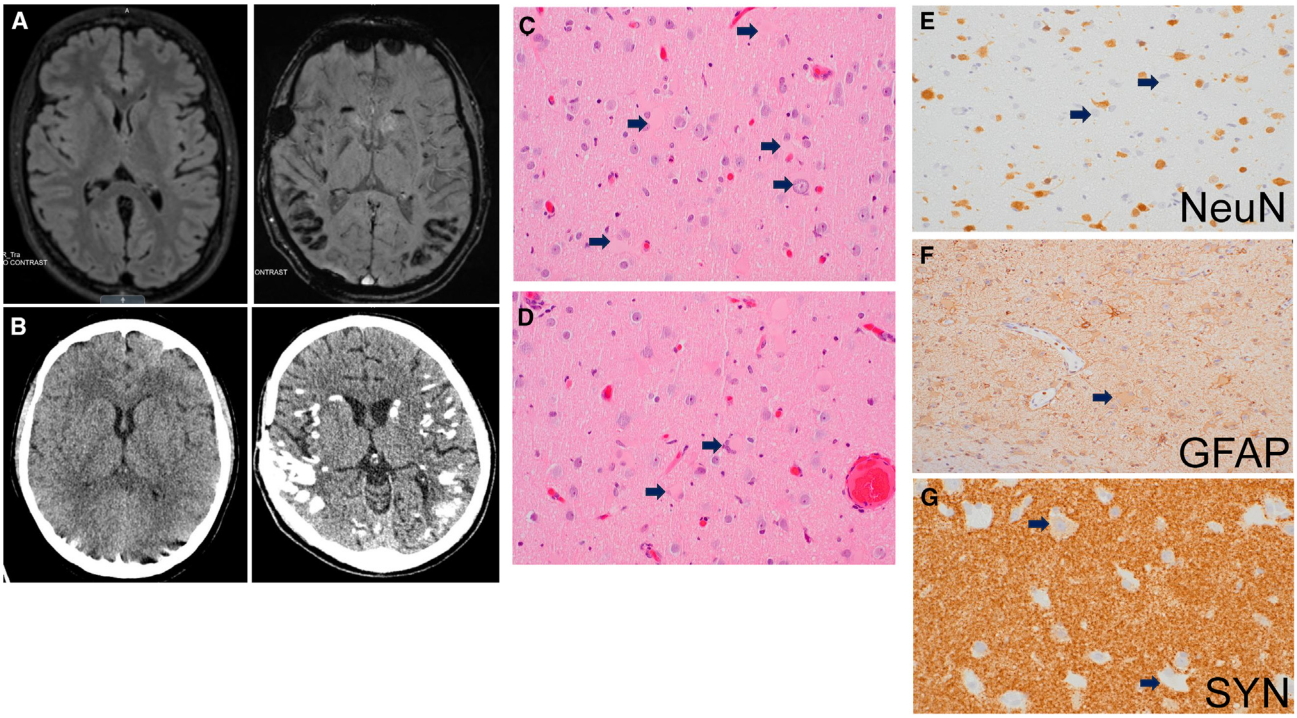

Figure 2. Cortical abnormalities in temporal lobe resected for drug-resistant epilepsy in individual 2 with *de novo HECTD1* p.Leu237Ser variant

(A) Magnetic resonance and (B) computed tomographic brain images in an axial section comparing control (left) to individual 2 (right).
(C and D) Hematoxylin and eosin-stained sections demonstrating dysmorphic and bi- to multinucleate balloon neurons with moderate to abundant glassy eosinophilic cytoplasm (arrows).
(E–G) Immunostaining highlights architectural disarray, i.e., cortical dyslamination (NeuN) and weak labeling of dysmorphic/balloon neurons with glial fibrillary acidic protein (GFAP) and synaptophysin (SYN).

referred to as UbGFP) (Figure 5B), resulting in the accumulation of polyubiquitin chains and fusion to GFP protein, resulting in UbGFP being targeted to the proteasome for degradation.[31,43] The reporter strain also co-expresses mRFP, which is not readily degraded by the UPS and serves as a normalization control. Proteasomal inhibition using bortezomib almost completely inhibited UbGFP degradation in wild-type animals, confirming that UbGFP was degraded by UPS (Figure S3). Moreover, Liu et al. also showed that loss of *hecd-1* in *C. elegans* led to a significant increase in accumulation of UbGFP and endogenous polyubiquitylated proteins.[31] Consistent with these findings, UbGFP levels (expressed as a ratio of GFP/mRFP) were elevated in the *hecd-1* (Δ) null mutants (Figures 5C and 5D). Treatment of *hecd-1* null mutants with bortezomib did not increase UbGFP levels (Figure S3), indicating that HECD-1-mediated degradation of UbGFP was proteasome dependent. UbGFP was also elevated in the p.Gly1345Ter variant animals at a similar level as the null (Δ) mutants (Figures 5C and 5D). A modest but significant increase in UbGFP accumulation was detected in the p.Asp1239Asn variant homozygous animals (Figure 5E). In contrast, the UbGFP level in the p.Arg350Gly variant homozygous animals was lower than in the control (p.Arg350Arg) and wild-type animals (Figure 5F), contrary to that of the p.Gly1345Ter variant homozygotes. Together, these results suggest the p.Gly1345Ter, p.Asp1239Asn, and p.Arg350Gly variants are all damaging to HECD-1 function. Moreover, the magnitude and direction of the changes of the GFP/mRFP ratio suggest that the p.Gly1345Ter variant behaves like a loss-of-function allele, while the p.Asp1239Asn and p.Arg350Gly variants behave like a weak hypomorph and a change of function (possibly a hyperactive gain of function), respectively.

Accumulation of ubiquitin-tagged proteins can also trigger cellular stress and activate the UPR pathway,[44] which can be studied in *C. elegans* using the *hsp-4p*::*GFP* reporter.[33] *hsp-4p*::*GFP* expression was modestly upregulated in p.Gly1345Ter variant homozygous animals compared to the wild-type controls (Figure S2G). No upregulation of *hsp-4*::*GFP* was observed in the p.Asp1239Asn variant. In contrast, *hsp-4*::*GFP* was decreased in the p.Arg350Gly variant compared to the wild-type control (Figure S2G). The opposing phenotype of the p.Arg350Gly variant compared to the p.Gly1345Ter loss-of-function variant provides additional support for the p.Arg350Gly variant being a change-of-function allele.

To determine whether the genetic mechanisms of the variants in *C. elegans* are consistent with a dominant presentation in humans, we assessed the UbGFP phenotype in variant heterozygotes. The UbGFP fluorescence in the *hecd-1* null heterozygotes was significantly elevated compared to the wild-type controls, indicating that *hecd-1* is haploinsufficient in *C. elegans* for this phenotype

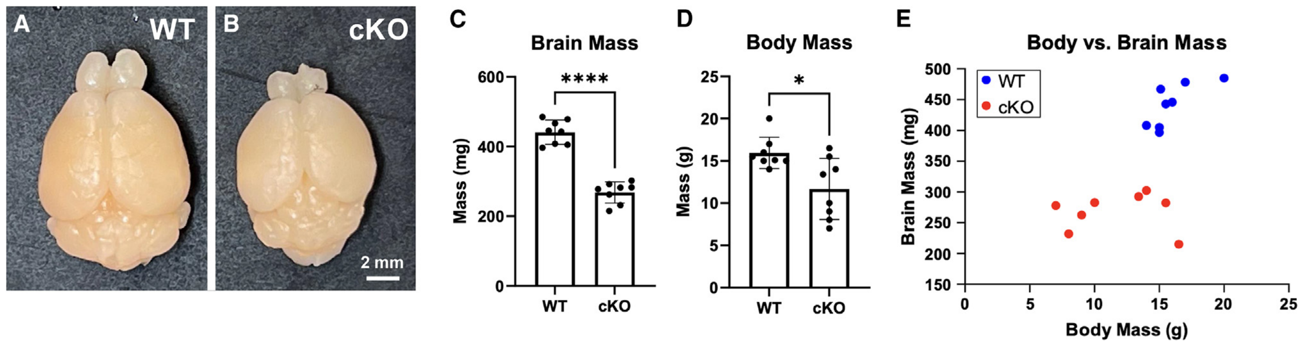

Figure 3. Sox1-Cre-mediated conditional knockout of *Hectd1* results in microcephaly
(A and B) Representative wild-type (WT) and conditional KO brains showing gross microcephaly.
(C) P30 cKO mice have significantly smaller brain mass compared to WT ($n = 8$, $p < 1e{-}4$).
(D) P30 cKO mice have variable but significantly diminished body weight compared to WT ($n = 8$, $p = 0.01$).
(E) No correlation between brain and body mass as all cKO brains are smaller, but only half of the mice had reduced body mass.

(Figure 5G). Elevated UbGFP fluorescence was also detected in the p.Asp1239Asn heterozygotes, albeit to a lesser extent. In contrast, the UbGFP fluorescence was decreased in the p.Arg350Gly heterozygotes (Figure 5H), again supporting this variant being a change-of-function allele.

In summary, evaluation of the p.Arg350Gly, p.Asp1239Asn, and p.Gly1345Ter variants indicated that all three variants act dominantly and are damaging to HECD-1 function in *C. elegans*. The p.Gly1345Ter variant was indistinguishable from the null mutant (Δ) for the phenotypes tested. The p.Asp1239Asn variant was characteristic of a weak hypomorphic allele, while the p.Arg350Gly variant behaved as a change-of-function variant. All variants showed phenotypes in heterozygous *C. elegans*. By extension, the human p.Arg371Gly (individual 3), p.Asp1116Asn (individual 5), and p.Gly1224Ter (individual 12) variants are likely damaging to *HECTD1* function and may act dominantly. Our data suggest that the human p.Arg371Gly variant may behave as a gain of function, while the p.Asp1116Asn and p.Gly1224Ter variants may behave in a haploinsufficient manner. While we did not specifically test the compound heterozygous variants found in individual 14, this individual had a frameshift variant inherited from an apparently healthy parent, which may suggest incomplete penetrance of the dominant inheritance in humans as well as the potential for additive effects of two damaging alleles.

Enrichment of *HECTD1* variants in published NDD and CHD cohorts

To further investigate the role of *HECTD1* variants in human disease, we performed an enrichment analysis of *de novo* variants in an independent cohort of 53,305 unique individuals diagnosed with either NDDs or CHD within published cohorts (Table S4) whose trio-based sequencing results are publicly available (Table S5). Only missense or likely gene-disruptive (stop-gained, splice-donor, splice-acceptor, frameshift) variants in *HECTD1* were included.[45] There were 12 missense and two likely gene-disruptive *de novo* variants in *HECTD1* in these published cohorts (Table S6). To test for enrichment, variants were analyzed with denovolyzeR.[46] These results show a 1.65-fold enrichment in *de novo* variants in *HECTD1* in the aggregate published cohorts using the denovolyzeR analysis ($p = 0.05$) (Table S7) and significant enrichment (4.02×10^{-6}) in the chimpanzee-human *de novo* variant test (Table S8), which incorporates locus-specific transition/transversion/indel rates and chimpanzee-human coding sequence divergence to estimate the expected *de novo* rates.[47] Both statistical tests showed greater enrichment of *HECTD1* missense variants than likely gene-disruptive variants.

Discussion

Our data reveal a critical role for *HECTD1*, a key mediator of the UPS pathway, in neural development and a genetic cause of human NDDs. Individuals with *HECTD1* variants exhibit a range of phenotypes, including neurodevelopmental disability, autism, ADHD, and epilepsy. In addition to our discovery of *de novo* likely gene-disruptive and missense variants and our demonstration of enrichment of *HECTD1* variants in published cohorts, several metrics of genomic constraint from gnomAD population data support *HECTD1* as a disease-causing gene.[14] First, *HECTD1* is constrained for missense variantion with a missense *Z* score of 6.43. Second, HECTD1 is constrained for loss-of-function variation with an observed/expected ratio of 0.27, indicating that it is likely a haploinsufficient gene.[22] In addition to the 14 individuals described here, at least four affected individuals in the DECIPHER database have <4 Mb deletions that include *HECTD1*, with no other known NDD genes located within the deleted interval.[48] Cross-disorder dosage sensitivity maps compiled from rare copy number variant data also support *HECTD1* as having a high probability of being both haploinsufficient (0.99) and triplosensitive (0.99).[49]

HECTD1 regulates numerous pathways that may contribute to NDDs, including signal transduction

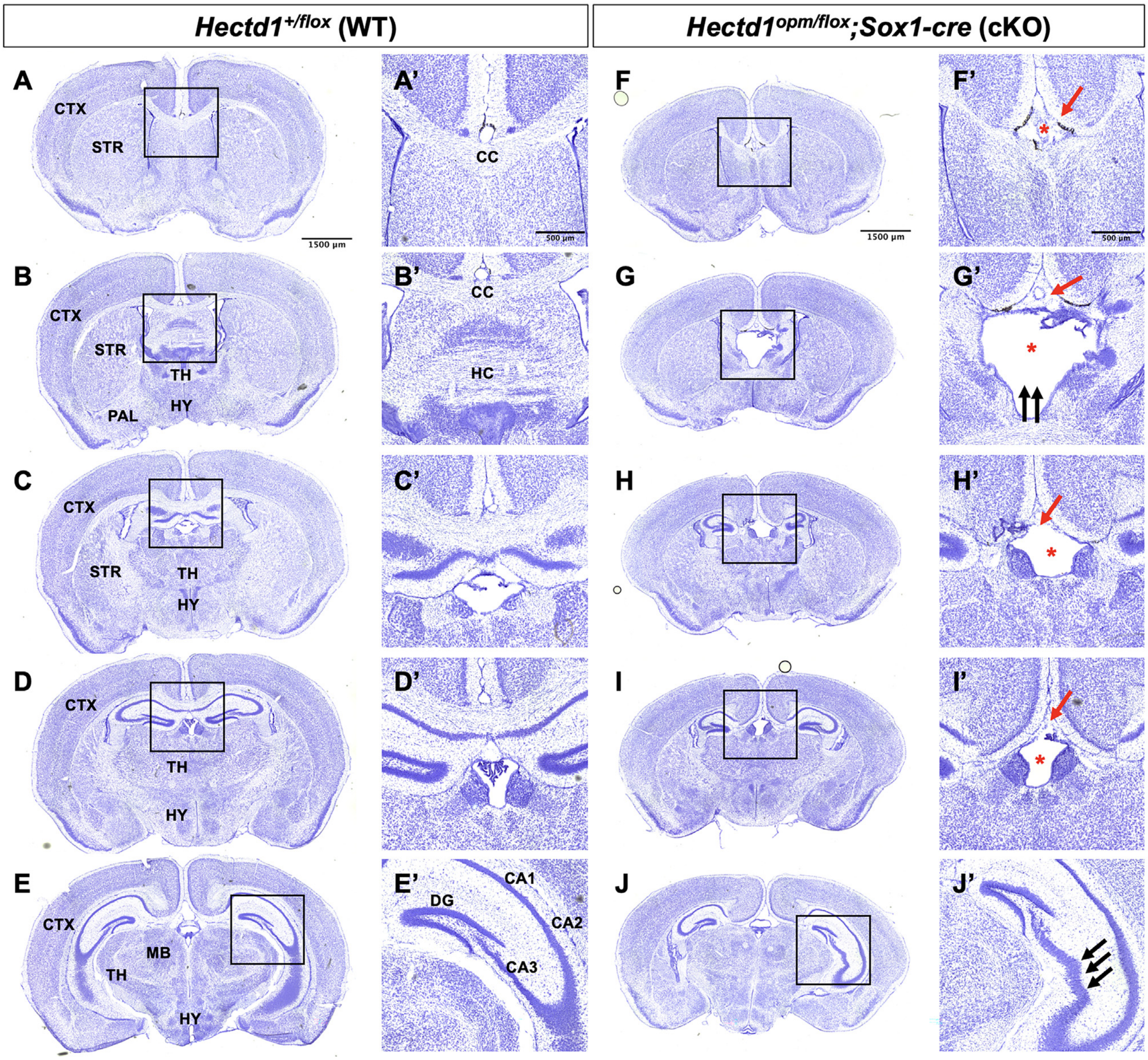

Figure 4. *Sox1-Cre*-mediated conditional knockout of *Hectd1* results in brain morphological phenotypes
(A–E) Nissl staining of a P30 wild-type (WT) littermate brain shown in serial sections from rostral to caudal.
(F–J) cKO sections are also shown from rostral to caudal. cKO brains appear smaller and exhibit several morphological abnormalities. Complete agenesis of the corpus callosum and ventricular dilation in cKO brain are shown by red arrow and star in (F′), (G′), (H′), and (I′). The CA3 hippocampal layer exhibits dysmorphology with abnormal bending and dispersion (black triple arrow in J′).
Data are representative of seven cKO and three WT sectioned brains from 11 litters, respectively. CTX, cerebral cortex: STR, striatum; TH, thalamus; HY, hypothalamus; PAL, pallidum; CC, corpus callosum; HC, hippocampal commissure; MB, midbrain; DG, dentate gyrus; CA1, CA2, and CA3 subfields.

pathways essential for brain development, such as Notch, Wnt, retinoic acid, and estrogen receptor signaling.[41,50–53] *HECTD1* regulates the striatin-interacting phosphatase and kinase (STRIPAK) complex, chromatin remodeling, cholesterol export, and protein translation,[10,42,50,54–56] influencing proliferation, apoptosis, autophagy, DNA-damage repair, and mitochondrial function.[55–62] *HECTD1* is also involved in inflammation in astrocytes, microglia, and other cell types and can regulate synaptic function by regulating the expression of glutamate transporter 1 (GLT-1) in astrocytes.[59,63–66] Effects on human development are not surprising given that *hecd-1*, the *C. elegans* ortholog of human *HECTD1*, also regulates Notch signaling, chromatin remodeling, and the expression of developmentally critical genes via the switch/sucrose non-fermentable (SWI/SNF) chromatin remodeling complex and katanin in mitosis and meiosis, thereby impacting the cell cycle.[41,42,56]

Additional individuals will need to be identified to establish genotype-phenotype correlations with specific *HECTD1* variants, particularly as this large protein regulates multiple substrates and pathways described above.

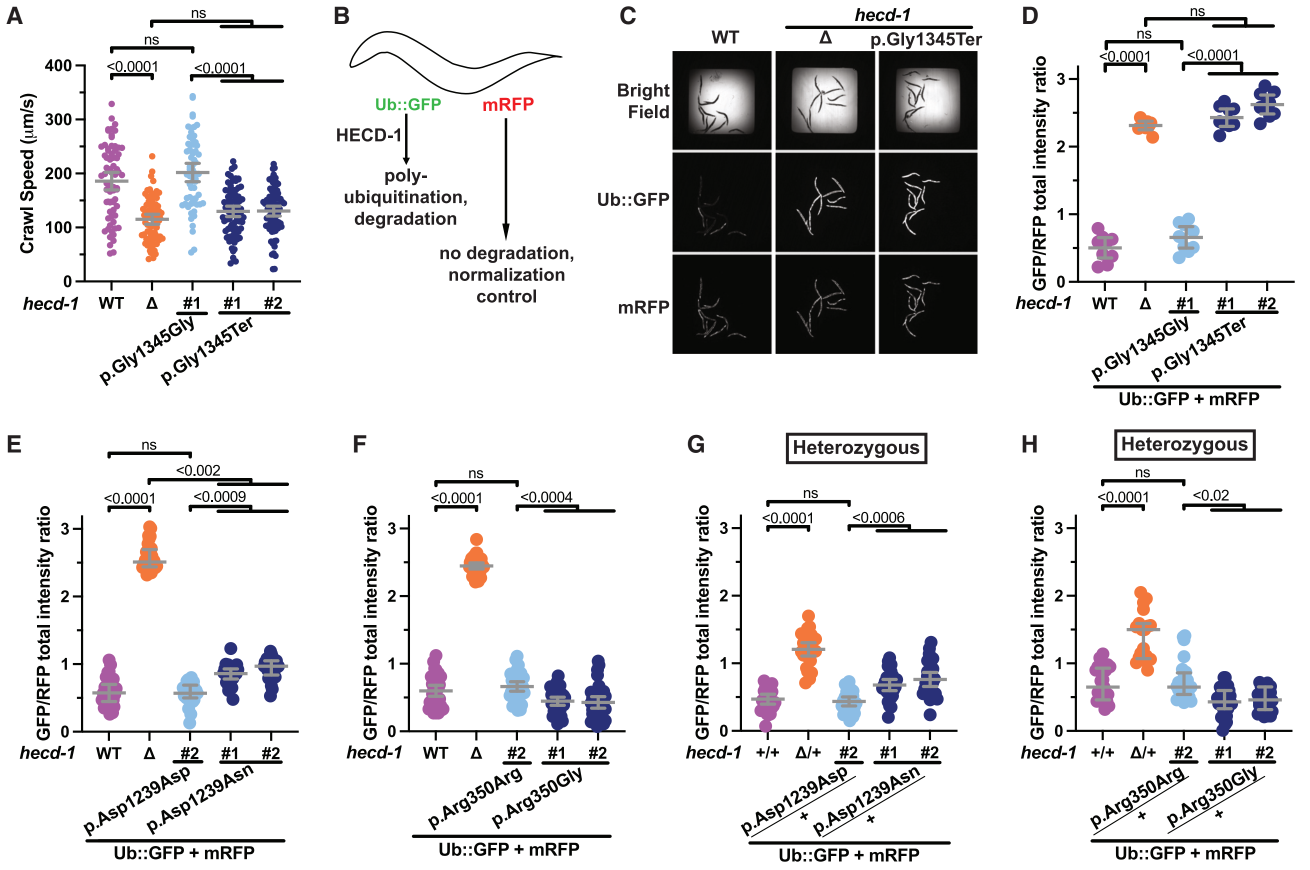

Figure 5. Functional analysis in *C. elegans* indicates that missense and nonsense variants are damaging to HECD-1 function
(A) Crawl speed of homozygous wild-type (WT, purple), null (Δ) mutants (orange), control edit (light blue), and two independent CRISPR-edited variant lines with the p.Gly1345Ter variant (dark blue) worms on agar plates determined by WormLab (MBF Bioscience). Each dot represents an animal. $n = 61–83$ for each genotype.
(B) Schematic showing how the Ub^{G76V}-GFP (abbreviated as UbGFP) reporter assay works. HECD-1 mediates the polyubiquitylation of the mutant, UbGFP. The mutation targets GFP for proteasomal degradation but not mRFP protein, which serves as a normalization control.
(C) Representative bright-field, GFP, and RFP images of UbGFP animals captured on the CX7 high-content imager (Thermo Fisher Scientific).
(D–F) Quantification of UbGFP accumulation (expressed as a ratio of GFP/RFP) in homozygous *hecd-1* p.Gly1345Ter (D), p.Asp1239Asn (E), and p.Arg350Gly (F) variants. Each dot represents a well containing approximately ten animals. $n = 9$ wells for (D); $n = 30–31$ wells for (E); $n = 35$ wells for (F).
(G and H) Quantification of UbGFP accumulation in heterozygous p.Asp1239Asn (G) and p.Arg350Gly (H) variants. $n = 25–27$ wells for (G); $n = 19–32$ wells for (H).
Error bars display the mean plus 95% confidence interval in (A), (D), (F), and (G) or the median plus interquartile range in (E) and (H).

While our conclusions are limited by the small number of variants we studied, our identification of variants that increase and decrease ubiquitylation in *C. elegans* suggests that there may be multiple genetic mechanisms of disease pathogenesis, including change of function and loss of function. While we saw no apparent phenotypic differences between individuals with missense variants and those with likely gene-disruptive variants, the one individual we identified with compound heterozygous inheritance had growth impairment along with NDD. Notably, this child inherited the missense and frameshift variants from apparently healthy parents, suggesting that genetic or environmental modifiers may be required to develop the phenotype. Microcephaly was present in this individual and two others, which may indicate more severe impairment in HECTD1 function, as seen with *Hectd1* deletion in the brain of the conditional mouse model. Although not all individuals underwent brain imaging, the imaging abnormalities were heterogeneous and more subtle than the striking hippocampal and callosal abnormalities observed in the conditional mouse model, likely due to the presence of a wild-type allele.

The phenotypic variability, ranging from mild speech delay to multiple congenital anomalies, suggests that individual variants may alter specific HECTD1 interactions with substrates and co-factors or that genetic or environmental modifiers contribute to these differences. For example, in mice, a heterozygous mutation of *Hectd1* interacts with vitamin A deficiency to modify aortic arch development.[12,52] Congenital cardiac anomalies were present in

only two of our 14 individuals, suggesting a higher penetrance of NDD phenotypes. Prior data from a study of >7,000 individuals with congenital heart disease described a single individual with compound heterozygous splice variants in *HECTD1*,[13] and our recent study identified rare *HECTD1* variants in individuals with NTDs.[14] Unfortunately, the co-occurrence of NDD phenotypes in the small number of individuals with CHD or NTDs and *HECTD1* variants was not explored in the prior studies, likely due to their ascertainment in infancy. Neural tube defects are notorious for complex oligogenic patterns of inheritance, where multiple genetic variants interact with environmental factors, and *HECTD1* has been proposed to contribute to human NTDs in this manner.[67,68] In aggregate, the human and mouse data demonstrate a requirement for *HECTD1* for neural tube, hematopoietic stem cell, heart, brain, and placental development[7–12] and suggest that *HECTD1* contributes to critical aspects of multiple early developmental processes.

Conclusions

We report here data indicating a role for *HECTD1* variants in neural development, including significant enrichment of *HECTD1 de novo* variants in an independent cohort of 53,305 published individuals with NDDs and CHD as well as brain abnormalities in conditional *Hectd1* mutant mice. Our investigation of a limited number of variants in a *C. elegans* model supports likely dominant inheritance with both change-of-function and loss-of-function/haploinsufficient mechanisms. Further work is needed to identify genotype-phenotype correlations for *HECTD1* variants, particularly because of the potential for distinct mechanisms caused by individual missense variants.

Data and code availability

This study did not generate or analyze datasets.

Acknowledgments

We thank the individuals and their family members who participated in this study.

Research reported in this publication was supported by the Washington University Institute of Clinical and Translational Sciences (UL1TR002345) from the National Center for Advancing Translational Sciences of the National Institutes of Health and the Eunice Kennedy Shriver National Institute of Child Health & Human Development (NICHD) of the National Institutes of Health under award number P50HD103525 to the Intellectual and Developmental Disabilities Research Center at Washington University. Support was also provided by R01HD110556 from NICHD (S.C.P. and colleagues L.S.-K., A.N.J., K.L.K., and D.M.O.) and by the Children's Discovery Institute, Brendan's Buddies, and St. Louis Children's Hospital Foundation (G.A.S. and S.C.P.). This work was supported by R01HD098861 from NICHD (I.Z.) and the molecular imaging core of the District of Columbia Intellectual and Developmental Disabilities Research Center (DC-IDDRC) award P50HD105328 from NICHD. One individual was sequenced by UCI-GREGoR funded by the National Human Genome Research Institute of the National Institutes of Health through the following grant, as part of GREGoR Consortium: 1U01HG011745. One individual underwent genome sequencing through the International Precision Child Health Partnership Gene-STEPS study, supported by the Boston Children's Hospital Children's Rare Disease Collaborative, the One8 Foundation, and the Robinson Family Initiative for Transformational Research. We thank Suk Regmi for technical assistance.

Author contributions

E.O., H.D.H., T.K., W.Y., J.G.C., K.I., S.S., K.L., B.W., and I.E.Z. undertook laboratory work and/or data analysis. G.Z.-J., E.O., H.D.H., T.S., S.C.P., I.E.Z., and C.A.G. conceived and led the study. G.Z.-J., E.O., T.N.T., J.G.C., S.C.P., I.E.Z., and C.A.G. wrote the manuscript. The other authors contributed individual data and case reports. All authors read and approved the manuscript prior to submission.

Declaration of interests

K.G.M. and M.J.G.S. are employees of GeneDx, LLC.

Web resources

NCBI RefSeq database, https://www.ncbi.nlm.nih.gov/refseq/.

Supplemental information

Supplemental information can be found online at https://doi.org/10.1016/j.ajhg.2025.01.001.

Received: June 20, 2024
Accepted: January 2, 2025
Published: January 28, 2025

References

1. Ebstein, F., Küry, S., Papendorf, J.J., and Krüger, E. (2021). Neurodevelopmental Disorders (NDD) Caused by Genomic Alterations of the Ubiquitin-Proteasome System (UPS): the Possible Contribution of Immune Dysregulation to Disease Pathogenesis. Front. Mol. Neurosci. *14*, 733012. https://doi.org/10.3389/fnmol.2021.733012.
2. Cruz Walma, D.A., Chen, Z., Bullock, A.N., and Yamada, K.M. (2022). Ubiquitin ligases: guardians of mammalian development. Nat. Rev. Mol. Cell Biol. *23*, 350–367. https://doi.org/10.1038/s41580-021-00448-5.
3. Ambrozkiewicz, M.C., Cuthill, K.J., Harnett, D., Kawabe, H., and Tarabykin, V. (2020). Molecular Evolution, Neurodevelopmental Roles and Clinical Significance of HECT-Type UBE3 E3 Ubiquitin Ligases. Cells *9*, 2455. https://doi.org/10.3390/cells9112455.
4. Kishino, T., Lalande, M., and Wagstaff, J. (1997). UBE3A/E6-AP mutations cause Angelman syndrome. Nat. Genet. *15*, 70–73. https://doi.org/10.1038/ng0197-70.
5. Matsuura, T., Sutcliffe, J.S., Fang, P., Galjaard, R.J., Jiang, Y.H., Benton, C.S., Rommens, J.M., and Beaudet, A.L. (1997). De novo truncating mutations in E6-AP ubiquitin-protein ligase gene (UBE3A) in Angelman syndrome. Nat. Genet. *15*, 74–77. https://doi.org/10.1038/ng0197-74.

6. Jolly, L.A., Kumar, R., Penzes, P., Piper, M., and Gecz, J. (2022). The DUB Club: Deubiquitinating Enzymes and Neurodevelopmental Disorders. Biol. Psychiatry *92*, 614–625. https://doi.org/10.1016/j.biopsych.2022.03.022.
7. Zohn, I.E., Anderson, K.V., and Niswander, L. (2007). The Hectd1 ubiquitin ligase is required for development of the head mesenchyme and neural tube closure. Dev. Biol. *306*, 208–221. https://doi.org/10.1016/j.ydbio.2007.03.018.
8. Sarkar, A.A., and Zohn, I.E. (2012). Hectd1 regulates intracellular localization and secretion of Hsp90 to control cellular behavior of the cranial mesenchyme. J. Cell Biol. *196*, 789–800. https://doi.org/10.1083/jcb.201105101.
9. Sarkar, A.A., Nuwayhid, S.J., Maynard, T., Ghandchi, F., Hill, J.T., Lamantia, A.S., and Zohn, I.E. (2014). Hectd1 is required for development of the junctional zone of the placenta. Dev. Biol. *392*, 368–380. https://doi.org/10.1016/j.ydbio.2014.05.007.
10. Lv, K., Gong, C., Antony, C., Han, X., Ren, J.G., Donaghy, R., Cheng, Y., Pellegrino, S., Warren, A.J., Paralkar, V.R., and Tong, W. (2021). HectD1 controls hematopoietic stem cell regeneration by coordinating ribosome assembly and protein synthesis. Cell Stem Cell *28*, 1275–1290.e9. https://doi.org/10.1016/j.stem.2021.02.008.
11. Sarkar, A.A., Sabatino, J.A., Sugrue, K.F., and Zohn, I.E. (2016). Abnormal labyrinthine zone in the Hectd1-null placenta. Placenta *38*, 16–23. https://doi.org/10.1016/j.placenta.2015.12.002.
12. Sugrue, K.F., Sarkar, A.A., Leatherbury, L., and Zohn, I.E. (2019). The ubiquitin ligase HECTD1 promotes retinoic acid signaling required for development of the aortic arch. Dis. Model. Mech. *12*, dmm036491. https://doi.org/10.1242/dmm.036491.
13. Jang, M.Y., Patel, P.N., Pereira, A.C., Willcox, J.A.L., Haghighi, A., Tai, A.C., Ito, K., Morton, S.U., Gorham, J.M., McKean, D.M., et al. (2023). Contribution of Previously Unrecognized RNA Splice-Altering Variants to Congenital Heart Disease. Circ. Genom. Precis. Med. *16*, 224–231. https://doi.org/10.1161/CIRCGEN.122.003924.
14. Oxman, E., Li, H., Wang, H.Y., and Zohn, I.E. (2024). Identification and functional analysis of rare HECTD1 missense variants in human neural tube defects. Hum. Genet. *143*, 263–277. https://doi.org/10.1007/s00439-024-02647-4.
15. Kosmicki, J.A., Samocha, K.E., Howrigan, D.P., Sanders, S.J., Slowikowski, K., Lek, M., Karczewski, K.J., Cutler, D.J., Devlin, B., Roeder, K., et al. (2017). Refining the role of de novo protein-truncating variants in neurodevelopmental disorders by using population reference samples. Nat. Genet. *49*, 504–510. https://doi.org/10.1038/ng.3789.
16. Turner, T.N., Wilfert, A.B., Bakken, T.E., Bernier, R.A., Pepper, M.R., Zhang, Z., Torene, R.I., Retterer, K., and Eichler, E.E. (2019). Sex-Based Analysis of De Novo Variants in Neurodevelopmental Disorders. Am. J. Hum. Genet. *105*, 1274–1285. https://doi.org/10.1016/j.ajhg.2019.11.003.
17. Lin, Y., Afshar, S., Rajadhyaksha, A.M., Potash, J.B., and Han, S. (2020). A Machine Learning Approach to Predicting Autism Risk Genes: Validation of Known Genes and Discovery of New Candidates. Front. Genet. *11*, 500064. https://doi.org/10.3389/fgene.2020.500064.
18. Sobreira, N., Schiettecatte, F., Valle, D., and Hamosh, A. (2015). GeneMatcher: a matching tool for connecting investigators with an interest in the same gene. Hum. Mutat. *36*, 928–930. https://doi.org/10.1002/humu.22844.
19. Kircher, M., Witten, D.M., Jain, P., O'Roak, B.J., Cooper, G.M., and Shendure, J. (2014). A general framework for estimating the relative pathogenicity of human genetic variants. Nat. Genet. *46*, 310–315. https://doi.org/10.1038/ng.2892.
20. Ioannidis, N.M., Rothstein, J.H., Pejaver, V., Middha, S., McDonnell, S.K., Baheti, S., Musolf, A., Li, Q., Holzinger, E., Karyadi, D., et al. (2016). REVEL: An Ensemble Method for Predicting the Pathogenicity of Rare Missense Variants. Am. J. Hum. Genet. *99*, 877–885. https://doi.org/10.1016/j.ajhg.2016.08.016.
21. Adzhubei, I.A., Schmidt, S., Peshkin, L., Ramensky, V.E., Gerasimova, A., Bork, P., Kondrashov, A.S., and Sunyaev, S.R. (2010). A method and server for predicting damaging missense mutations. Nat. Methods *7*, 248–249. https://doi.org/10.1038/nmeth0410-248.
22. Karczewski, K.J., Francioli, L.C., Tiao, G., Cummings, B.B., Alföldi, J., Wang, Q., Collins, R.L., Laricchia, K.M., Ganna, A., Birnbaum, D.P., et al. (2020). The mutational constraint spectrum quantified from variation in 141,456 humans. Nature *581*, 434–443. https://doi.org/10.1038/s41586-020-2308-7.
23. Jumper, J., Evans, R., Pritzel, A., Green, T., Figurnov, M., Ronneberger, O., Tunyasuvunakool, K., Bates, R., Žídek, A., Potapenko, A., et al. (2021). Highly accurate protein structure prediction with AlphaFold. Nature *596*, 583–589. https://doi.org/10.1038/s41586-021-03819-2.
24. Varadi, M., Bertoni, D., Magana, P., Paramval, U., Pidruchna, I., Radhakrishnan, M., Tsenkov, M., Nair, S., Mirdita, M., Yeo, J., et al. (2024). AlphaFold Protein Structure Database in 2024: providing structure coverage for over 214 million protein sequences. Nucleic Acids Res. *52*, D368–D375. https://doi.org/10.1093/nar/gkad1011.
25. Takashima, Y., Era, T., Nakao, K., Kondo, S., Kasuga, M., Smith, A.G., and Nishikawa, S.I. (2007). Neuroepithelial cells supply an initial transient wave of MSC differentiation. Cell *129*, 1377–1388. https://doi.org/10.1016/j.cell.2007.04.028.
26. Raymond, C.S., and Soriano, P. (2007). High-efficiency FLP and PhiC31 site-specific recombination in mammalian cells. PLoS One *2*, e162. https://doi.org/10.1371/journal.pone.0000162.
27. Brenner, S. (1974). The genetics of Caenorhabditis elegans. Genetics *77*, 71–94. https://doi.org/10.1093/genetics/77.1.71.
28. Huang, H., Pan, J., Spielberg, D.R., Hanchard, N.A., Scott, D.A., Burrage, L.C., Dai, H., Murdock, D., Rosenfeld, J.A., Mohammad, A., et al. (2022). A dominant negative variant of RAB5B disrupts maturation of surfactant protein B and surfactant protein C. Proc. Natl. Acad. Sci. USA *119*, e2105228119. https://doi.org/10.1073/pnas.2105228119.
29. Sternberg, P.W., Van Auken, K., Wang, Q., Wright, A., Yook, K., Zarowiecki, M., Arnaboldi, V., Becerra, A., Brown, S., Cain, S., et al. (2024). WormBase 2024: status and transitioning to Alliance infrastructure. Genetics *227*, iyae050. https://doi.org/10.1093/genetics/iyae050.
30. Marom, R., Zhang, B., Washington, M.E., Song, I.W., Burrage, L.C., Rossi, V.C., Berrier, A.S., Lindsey, A., Lesinski, J., Nonet, M.L., et al. (2023). Dominant negative variants in KIF5B cause osteogenesis imperfecta via down regulation of mTOR signaling. PLoS Genet. *19*, e1011005. https://doi.org/10.1371/journal.pgen.1011005.
31. Liu, G., Rogers, J., Murphy, C.T., and Rongo, C. (2011). EGF signalling activates the ubiquitin proteasome system to modulate C. elegans lifespan. EMBO J. *30*, 2990–3003. https://doi.org/10.1038/emboj.2011.195.

32. Dawson, Z.D., Sundaramoorthi, H., Regmi, S., Zhang, B., Morrison, S., Fielder, S.M., Zhang, J.R., Hoang, H., Perlmutter, D.H., Luke, C.J., et al. (2024). A fluorescent reporter for rapid assessment of autophagic flux reveals unique autophagy signatures during C. elegans post-embryonic development and identifies compounds that modulate autophagy. Autophagy Rep. *3*, 2371736. https://doi.org/10.1080/27694127.2024.2371736.
33. Darom, A., Bening-Abu-Shach, U., and Broday, L. (2010). RNF-121 is an endoplasmic reticulum-membrane E3 ubiquitin ligase involved in the regulation of beta-integrin. Mol. Biol. Cell *21*, 1788–1798. https://doi.org/10.1091/mbc.e09-09-0774.
34. Gudmundsson, S., Singer-Berk, M., Watts, N.A., Phu, W., Goodrich, J.K., Solomonson, M., Genome Aggregation Database Consortium, Rehm, H.L., MacArthur, D.G., and O'Donnell-Luria, A. (2022). Variant interpretation using population databases: Lessons from gnomAD. Hum. Mutat. *43*, 1012–1030. https://doi.org/10.1002/humu.24309.
35. Lek, M., Karczewski, K.J., Minikel, E.V., Samocha, K.E., Banks, E., Fennell, T., O'Donnell-Luria, A.H., Ware, J.S., Hill, A.J., Cummings, B.B., et al. (2016). Analysis of protein-coding genetic variation in 60,706 humans. Nature *536*, 285–291. https://doi.org/10.1038/nature19057.
36. Fischer-Zirnsak, B., Segebrecht, L., Schubach, M., Charles, P., Alderman, E., Brown, K., Cadieux-Dion, M., Cartwright, T., Chen, Y., Costin, C., et al. (2019). Haploinsufficiency of the Notch Ligand DLL1 Causes Variable Neurodevelopmental Disorders. Am. J. Hum. Genet. *105*, 631–639. https://doi.org/10.1016/j.ajhg.2019.07.002.
37. Kane, E.I., and Spratt, D.E. (2021). Structural Insights into Ankyrin Repeat-Containing Proteins and Their Influence in Ubiquitylation. Int. J. Mol. Sci. *22*, 609. https://doi.org/10.3390/ijms22020609.
38. Starr, D.A. (2011). KASH and SUN proteins. Curr. Biol. *21*, R414–R415. https://doi.org/10.1016/j.cub.2011.04.022.
39. Domsch, K., Acs, A., Obermeier, C., Nguyen, H.T., and Reim, I. (2017). Identification of the essential protein domains for Mib2 function during the development of the Drosophila larval musculature and adult flight muscles. PLoS One *12*, e0173733. https://doi.org/10.1371/journal.pone.0173733.
40. Arnold, S.J., Huang, G.J., Cheung, A.F.P., Era, T., Nishikawa, S.I., Bikoff, E.K., Molnár, Z., Robertson, E.J., and Groszer, M. (2008). The T-box transcription factor Eomes/Tbr2 regulates neurogenesis in the cortical subventricular zone. Genes Dev. *22*, 2479–2484. https://doi.org/10.1101/gad.475408.
41. Chen, Y., and Greenwald, I. (2014). hecd-1 modulates notch activity in Caenorhabditis elegans. G3 (Bethesda) *5*, 353–359. https://doi.org/10.1534/g3.114.015321.
42. Lampersberger, L., Conte, F., Ghosh, S., Xiao, Y., Price, J., Jordan, D., Matus, D.Q., Sarkies, P., Beli, P., Miska, E.A., and Burton, N.O. (2023). Loss of the E3 ubiquitin ligases UBR-5 or HECD-1 restores Caenorhabditis elegans development in the absence of SWI/SNF function. Proc. Natl. Acad. Sci. USA *120*, e2217992120. https://doi.org/10.1073/pnas.2217992120.
43. Dantuma, N.P., Lindsten, K., Glas, R., Jellne, M., and Masucci, M.G. (2000). Short-lived green fluorescent proteins for quantifying ubiquitin/proteasome-dependent proteolysis in living cells. Nat. Biotechnol. *18*, 538–543. https://doi.org/10.1038/75406.
44. Qu, J., Zou, T., and Lin, Z. (2021). The Roles of the Ubiquitin-Proteasome System in the Endoplasmic Reticulum Stress Pathway. Int. J. Mol. Sci. *22*, 1526. https://doi.org/10.3390/ijms22041526.
45. McLaren, W., Gil, L., Hunt, S.E., Riat, H.S., Ritchie, G.R.S., Thormann, A., Flicek, P., and Cunningham, F. (2016). The Ensembl Variant Effect Predictor. Genome Biol. *17*, 122. https://doi.org/10.1186/s13059-016-0974-4.
46. Ware, J.S., Samocha, K.E., Homsy, J., and Daly, M.J. (2015). Interpreting de novo Variation in Human Disease Using denovolyzeR. Curr. Protoc. Hum. Genet. *87*, 7.25.1–7.25.15. https://doi.org/10.1002/0471142905.hg0725s87.
47. Coe, B.P., Stessman, H.A.F., Sulovari, A., Geisheker, M.R., Bakken, T.E., Lake, A.M., Dougherty, J.D., Lein, E.S., Hormozdiari, F., Bernier, R.A., and Eichler, E.E. (2019). Neurodevelopmental disease genes implicated by de novo mutation and copy number variation morbidity. Nat. Genet. *51*, 106–116. https://doi.org/10.1038/s41588-018-0288-4.
48. Firth, H.V., Richards, S.M., Bevan, A.P., Clayton, S., Corpas, M., Rajan, D., Van Vooren, S., Moreau, Y., Pettett, R.M., and Carter, N.P. (2009). DECIPHER: Database of Chromosomal Imbalance and Phenotype in Humans Using Ensembl Resources. Am. J. Hum. Genet. *84*, 524–533. https://doi.org/10.1016/j.ajhg.2009.03.010.
49. Collins, R.L., Glessner, J.T., Porcu, E., Lepamets, M., Brandon, R., Lauricella, C., Han, L., Morley, T., Niestroj, L.M., Ulirsch, J., et al. (2022). A cross-disorder dosage sensitivity map of the human genome. Cell *185*, 3041–3055.e25. https://doi.org/10.1016/j.cell.2022.06.036.
50. Li, W., Hu, Y., Oh, S., Ma, Q., Merkurjev, D., Song, X., Zhou, X., Liu, Z., Tanasa, B., He, X., et al. (2015). Condensin I and II Complexes License Full Estrogen Receptor alpha-Dependent Enhancer Activation. Mol. Cell *59*, 188–202. https://doi.org/10.1016/j.molcel.2015.06.002.
51. Oikonomaki, M., Bady, P., and Hegi, M.E. (2017). Ubiquitin Specific Peptidase 15 (USP15) suppresses glioblastoma cell growth via stabilization of HECTD1 E3 ligase attenuating WNT pathway activity. Oncotarget *8*, 110490–110502. https://doi.org/10.18632/oncotarget.22798.
52. Sugrue, K.F., and Zohn, I.E. (2019). Reduced maternal vitamin A status increases the incidence of normal aortic arch variants. Genesis *57*, e23343. https://doi.org/10.1002/dvg.23343.
53. Tran, H., Bustos, D., Yeh, R., Rubinfeld, B., Lam, C., Shriver, S., Zilberleyb, I., Lee, M.W., Phu, L., Sarkar, A.A., et al. (2013). HectD1 E3 ligase modifies adenomatous polyposis coli (APC) with polyubiquitin to promote the APC-axin interaction. J. Biol. Chem. *288*, 3753–3767. https://doi.org/10.1074/jbc.M112.415240.
54. Aleidi, S.M., Yang, A., Sharpe, L.J., Rao, G., Cochran, B.J., Rye, K.A., Kockx, M., Brown, A.J., and Gelissen, I.C. (2018). The E3 ubiquitin ligase, HECTD1, is involved in ABCA1-mediated cholesterol export from macrophages. Biochim. Biophys. Acta. Mol. Cell Biol. Lipids *1863*, 359–368. https://doi.org/10.1016/j.bbalip.2017.12.011.
55. Beard, S.M., Smit, R.B., Chan, B.G., and Mains, P.E. (2016). Regulation of the MEI-1/MEI-2 Microtubule-Severing Katanin Complex in Early Caenorhabditis elegans Development. G3 (Bethesda) *6*, 3257–3268. https://doi.org/10.1534/g3.116.031666.
56. Lu, T., Smit, R.B., Soueid, H., and Mains, P.E. (2022). STRIPAK regulation of katanin microtubule severing in the Caenorhabditis elegans embryo. Genetics *221*, iyac043. https://doi.org/10.1093/genetics/iyac043.

57. Bennett, L., Madders, E.C.E.T., and Parsons, J.L. (2020). HECTD1 promotes base excision repair in nucleosomes through chromatin remodelling. Nucleic Acids Res. *48*, 1301–1313. https://doi.org/10.1093/nar/gkz1129.
58. Duhamel, S., Goyette, M.A., Thibault, M.P., Filion, D., Gaboury, L., and Côté, J.F. (2018). The E3 Ubiquitin Ligase HectD1 Suppresses EMT and Metastasis by Targeting the +TIP ACF7 for Degradation. Cell Rep. *22*, 1016–1030. https://doi.org/10.1016/j.celrep.2017.12.096.
59. Liao, S., Zheng, Q., Shen, H., Yang, G., Xu, Y., Zhang, X., Ouyang, H., and Pan, Z. (2023). HECTD1-Mediated Ubiquitination and Degradation of Rubicon Regulates Autophagy and Osteoarthritis Pathogenesis. Arthritis Rheumatol. *75*, 387–400. https://doi.org/10.1002/art.42369.
60. Segref, A., Kevei, É., Pokrzywa, W., Schmeisser, K., Mansfeld, J., Livnat-Levanon, N., Ensenauer, R., Glickman, M.H., Ristow, M., and Hoppe, T. (2014). Pathogenesis of human mitochondrial diseases is modulated by reduced activity of the ubiquitin/proteasome system. Cell Metab. *19*, 642–652. https://doi.org/10.1016/j.cmet.2014.01.016.
61. Uemoto, Y., Katsuta, E., Kondo, N., Wanifuchi-Endo, Y., Fujita, T., Asano, T., Hisada, T., Terada, M., Kato, A., Okuda, K., et al. (2022). Low HECTD1 mRNA expression is associated with poor prognosis and may be correlated with increased mitochondrial respiratory function in breast cancer. Am. J. Cancer Res. *12*, 1593–1605.
62. Vaughan, N., Scholz, N., Lindon, C., and Licchesi, J.D.F. (2022). The E3 ubiquitin ligase HECTD1 contributes to cell proliferation through an effect on mitosis. Sci. Rep. *12*, 13160. https://doi.org/10.1038/s41598-022-16965-y.
63. Li, Y., Huang, B., Yang, H., Kan, S., Yao, Y., Liu, X., Pu, S., He, G., Khan, T.M., Qi, G., et al. (2020). Latexin deficiency in mice up-regulates inflammation and aggravates colitis through HECTD1/Rps3/NF-kappaB pathway. Sci. Rep. *10*, 9868. https://doi.org/10.1038/s41598-020-66789-x.
64. Tang, Y., Zhou, M., Huang, R., Shen, L., Yang, L., Zhou, Z., Ren, H., and Bai, Y. (2021). Involvement of HECTD1 in LPS-induced astrocyte activation via sigma-1R-JNK/p38-FOXJ2 axis. Cell Biosci. *11*, 62. https://doi.org/10.1186/s13578-021-00572-x.
65. Zhang, Y., Du, L., Bai, Y., Han, B., He, C., Gong, L., Huang, R., Shen, L., Chao, J., Liu, P., et al. (2020). CircDYM ameliorates depressive-like behavior by targeting miR-9 to regulate microglial activation via HSP90 ubiquitination. Mol. Psychiatry *25*, 1175–1190. https://doi.org/10.1038/s41380-018-0285-0.
66. Zeng, X., Dong, X., Xiao, Q., and Yao, J. (2022). Vitamin C Inhibits Ubiquitination of Glutamate Transporter 1 (GLT-1) in Astrocytes by Downregulating HECTD1. ACS Chem. Neurosci. *13*, 676–687. https://doi.org/10.1021/acschemneuro.1c00845.
67. Finnell, R.H., Caiaffa, C.D., Kim, S.E., Lei, Y., Steele, J., Cao, X., Tukeman, G., Lin, Y.L., Cabrera, R.M., and Wlodarczyk, B.J. (2021). Gene Environment Interactions in the Etiology of Neural Tube Defects. Front. Genet. *12*, 659612. https://doi.org/10.3389/fgene.2021.659612.
68. Wolujewicz, P., Steele, J.W., Kaltschmidt, J.A., Finnell, R.H., and Ross, M.E. (2021). Unraveling the complex genetics of neural tube defects: From biological models to human genomics and back. Genesis *59*, e23459. https://doi.org/10.1002/dvg.23459.

The American Journal of Human Genetics, Volume *112*

Supplemental information

Sequence variants in *HECTD1* result in a variable neurodevelopmental disorder

Gazelle Zerafati-Jahromi, Elias Oxman, Hieu D. Hoang, Wu-Lin Charng, Tanvitha Kotla, Weimin Yuan, Keito Ishibashi, Sonia Sebaoui, Kathryn Luedtke, Bryce Winrow, Rebecca D. Ganetzky, Anna Ruiz, Carmen Manso-Basúz, Nino Spataro, Peter Kannu, Taryn Athey, Christina Peroutka, Caitlin Barnes, Richard Sidlow, George Anadiotis, Kari Magnussen, Irene Valenzuela, Alejandro Moles-Fernandez, Seth Berger, Christina L. Grant, Eric Vilain, Gudny A. Arnadottir, Patrick Sulem, Telma S. Sulem, Kari Stefansson, Shavonne Massey, Natalie Ginn, Annapurna Poduri, Alissa M. D'Gama, Rozalia Valentine, Sara K. Trowbridge, Chaya N. Murali, Rachel Franciskovich, Yen Tran, Bryn D. Webb, Kim M. Keppler-Noreuil, April L. Hall, Bobbi McGivern, Kristin G. Monaghan, Maria J. Guillen Sacoto, Dustin Baldridge, Gary A. Silverman, Sonika Dahiya, Tychele N. Turner, Tim Schedl, Joshua G. Corbin, Stephen C. Pak, Irene E. Zohn, and Christina A. Gurnett

Case Reports

Individual 1

Individual 1 is a 10 year old female with epilepsy, autism and language delay. She was born at 37 weeks gestational age via C-section due to maternal diabetes. Growth parameters at the time of last evaluation at 9 years of age included OFC of 55.5cm (>97%ile), weight of 80.7kg (100%ile) and height of 145.4cm (86%ile). She has been diagnosed with obesity. Hearing and vision assessments were normal. Her first words were at 3 years of age, and presently she does not speak in full sentences. She has also been diagnosed with autism. She receives PT, OT, ST and also has an IEP at school which includes special education. She has frontal lobe epilepsy, with EEG demonstrating left frontal focal epileptiform abnormalities. Brain MRI demonstrated a 3mm area of T2 signal abnormality in the inferior left temporal lobe. Cardiac evaluation was normal.

Individual 2

Individual 2 is a 28 year old male who presented with focal onset status epilepticus at age 8. He was diagnosed with ADHD and mild cognitive impairment prior to the onset of epilepsy. He had short stature but otherwise normal growth parameters. By age 9, he developed drug resistant epilepsy with seizures arising from the left temporal lobe. MRI showed bilateral posterior temporooccipital lobe cerebral calcifications and white matter T2/FLAIR hyperintense foci involving the left temporal lobe. Left temporal lobe resection at age 9 for intractable epilepsy demonstrated ILAE Type IIb focal cortical dysplasia characterized by presence of dysmorphic and balloon neurons (Figure 2). After surgery, there were no further generalized tonic clonic seizures, but other seizures continued. His course has subsequently been characterized by progressive neurocognitive dysfunction, epilepsy, stroke-like episodes particularly of the occipital lobes, cortical visual impairment, suspected transient ischemic attacks with vision loss, weakness or right facial droop. His seizures are characterized by (1) visual hallucinations lasting up to hours with retained awareness, (2) staring and unresponsiveness, and (3) rare generalized tonic clonic activity. He has transient episodes of unclear etiology with sudden full loss of vision in both eyes or inability to walk lasting several minutes with nearly complete resolution. Brain MRIs have shown progressive occipital white matter hyperintensities and subcortical white matter T2/FLAIR hyperintense foci that progressively evolved over time to cystic encephalomalacia with volume loss of the occipital lobes (Figure 2). In addition to multiple antiepileptic medications, he was treated with IVIG and mycophenolate mofetil which decreased the frequency of the episodes for a presumed autoimmune condition despite negative autoantibodies. He also has kidney stones with end stage kidney failure, and bradycardia of unclear etiology with normal echocardiogram. He is employed part-time at a grocery store.

Individual 3

Individual 3 is a 5-year-old male with epilepsy, mild global developmental delay and persistently elevated transaminases. He was born at 37 weeks via induced vaginal delivery. Growth parameters at delivery included weight of 3232g (74%ile, Z-score 0.63) and length of 45.7cm (14%ile, Z-score -1.06). At time of last evaluation at 3 years of age, OFC was at 20%ile (Z-score 0.86), weight was at 29%ile (Z-score 0.55) and height was at 15%ile (Z-score 1.00). No facial dysmorphisms were noted. Hearing and vision evaluations were normal. However, developmental abnormalities were reported in all domains, requiring physical therapy and speech therapy. He walked at 15 months, but he was noted to have imbalance out of proportion to his age. He demonstrated left hand preference at 12

months of age. He said his first word at 15 months of age and was not speaking in full sentences at 20 months old, however speech rapidly normalized with placement of myringotomy tubes. He also required G-tube feedings until 3 years of age. He developed temporal lobe epilepsy, with EEG demonstrating seizures arising from the left temporal lobe at one month of age, with clinical semiology of spasticity and cyanosis. Brain MRI, however, was normal. At the time of last evaluation at 3.5 years of age, he had complete seizure control and normalization of the EEG on levetiracetam, as well as normal development. Bloodwork demonstrated elevated transaminases, therefore liver biopsy was performed, demonstrating abnormal mitochondria on electron microscopy, absence of complex I activity, decreased complex IV activity, and abnormal complex V assembly. Cardiac evaluation was normal.

Individual 4

Individual 4 is a 7 year old girl with global developmental delay, behavioral problems as well as movement disorder. She was born at 41 weeks gestational age by vaginal delivery. She was noted to be macrocephalic at birth and weighed 3940g (74%ile). At the time of last evaluation, OFC was 54cm (>97%ile), weight was 29.8kg (92%ile), and height was 124cm (67%ile). On exam she was noted to have bitemporal narrowing, high palate and hypotonia. She demonstrated poor latch and choking in infancy, which progressed to oral dysphagia by age two, thereby necessitating placement of a G-tube by which she receives all nutrition. The most recent swallow evaluation within the last year was normal. She was also diagnosed with severe central and obstructive sleep apnea at 14 months of age. She underwent tonsillectomy and adenoidectomy but was not able to tolerate BiPAP. Presently her central sleep apnea has mostly resolved. Although she walked on time around 13-14 months of age, she is noted to be clumsy and displays toe walking. She does not use any tools such as utensils. She spoke her first words around 12 months of age but subsequently stopped gaining new words, and now is non-verbal aside from the use of an augmentative communication device for single word communication. She was initially diagnosed with sensorineural hearing loss, however at age 4 was found to have normal hearing at which point hearing aids were discontinued. She has been diagnosed with severe autism and has self-injurious behaviors, intense tantrums, rigidity, aggression, poor emotional regulation, and poor frustration tolerance. She also displays stereotypic movements diagnosed as possible tic disorder. She receives PT, OT, ST and ABA, and also receives special education. Brain MRI at 5 years old demonstrated stable small pars intermedia pituitary cyst, as well as mild diffuse thickening of the corpus callosum. EEG at 5 years old was normal. At age 7, she had 3 short events concerning for seizure activity characterized by eyelid fluttering, and 1 event with lack of awareness, incontinence and full body shaking. Repeat EEG was again normal. She is not prescribed any anti-seizure medications and has not had any further events. The most recent brain MRI at 7 years old demonstrated mild under rotation and dysgenesis of the left hippocampus, which was felt to be unrelated to these events. ECHO and EKG were normal. Holter monitor demonstrated sinus bradycardia.

Individual 5

Individual 5 is an 18 year old male with mild intellectual disability and language impairments. He was born at 40 weeks by vaginal delivery. Growth parameters at birth included weight of 3317g (29%ile, Z-score -0.54). He had poor latch in infancy, and as an older child has frequent GI symptoms concerning for irritable bowel syndrome. He was noted to have fine motor difficulties as well as difficulties with verbal communication. Hearing and vision evaluations were normal. He has also been

diagnosed with ADHD and mild intellectual disability, although he did not receive therapy services or special education. At the time of the last evaluation at 18 years of age, OFC was 53cm (<3%ile), weight was 57kg (13%ile), and height was 185cm (89%ile). Malar hypoplasia, prominent jaw, arachnodactyly, pectus carinatum, pes planus, mild kyphosis, and cryptorchidism were noted on exam. Brain MRI was normal. ECHO demonstrated mild dilation of the ascending aorta. Marfan/LDS panel was sent, demonstrating paternally-inherited VUS in COL5A1. Bloodwork demonstrated decreased level of IgA.

Individual 6

Individual 6 is a 9 month old full-term boy with epilepsy and sleep apnea. He was born at 40 weeks gestational age by spontaneous vaginal delivery. Growth parameters at delivery were OFC of 36cm (75%ile), weight of 3480g (43%ile) and length of 47cm (3%ile). At 2 months of age, OFC was 40.5cm (65%ile), weight was 4920g (31%ile) and length was 57.5cm (40%ile). Exam demonstrated mildly low axial tone. No facial dysmorphisms were noted on exam. He passed a hearing screening on day of life 1. On day of life 3 he developed focal seizures characterized by staring, paucity of movement, apnea, profound desaturation and whole-body cyanosis. EEG captured 2 focal seizures arising from the left occipital region which evolved to spread into the left temporal region. Brain MRI on day of life 5 demonstrated trace right posterior convexity subdural hematoma but was otherwise unremarkable. Sleep study at 15 days old demonstrated moderate to severe central apnea and mild obstructive apnea, requiring continuous supplemental oxygen by nasal cannula. Modified barium swallow study at 16 days old demonstrated aspiration, which resolved with use of preemie flow nipple. As of the most recent clinical update at 6 months of age, he was weaned off supplemental oxygen and thus far his development has been normal.

Individual 7

Individual 7 is a 9 month old male with history of Tetralogy of Fallot status post cardiac surgery, Pierre Robin sequence (with Veau class II cleft palate), and dysmorphic features. Pregnancy was complicated by mother with type 2 diabetes mellitus and obesity. Prenatal history is notable for diagnosis of a congenital heart defect after 20-week anatomy scan and fetal echocardiogram. He was born via C-section at 38 weeks gestational age due to breech presentation, prolonged rupture of membranes, and known congenital heart disease. Growth parameters at delivery included weight of 3.18kg (36 %ile), length of 50cm (52%ile) and OFC of 33cm (12 %ile). He was diagnosed with Tetralogy of Fallot (TOF) with severe pulmonary vein stenosis and small right ventricular outflow tract. His first cardiac surgery was at 2 weeks of age, with subsequent repair of TOF completed at 4 months of age. He also was found to have Pierre-Robin sequence with Veau class II cleft palate and had reflux and aspiration. Newborn hearing screen was normal. At 3 weeks of age a G-tube was placed, a right inguinal hernia was repaired, and left orchioplexy was attempted but was not successful. At 5 weeks of age, a hip ultrasound revealed congenital bilateral dislocation of the hips. At 7 weeks of age, a supraglottoplasty was performed due to laryngomalacia. At 6 months old, OFC was 40.2 cm (O.44%, Z=-2.62), weight was 6.18 kg (0.32%, Z=-2.72), and height was 65.8 cm (4.75%, Z=-1.67). Dysmorphic features included microcephaly, large, simple, and protruding ears, blue sclera, downslanting palpebral fissures, epicanthal folds, long philtrum, a small, downturned mouth, and microretrognathia. He had prominent truncal and axial hypotonia and was unable to roll over or push himself up when prone. He is G-tube dependent. Due to concerns for abnormal movements an EEG was obtained, which was normal. At 8 months old, a sleep study revealed sleep apnea with tongue

obstruction. At 9 months of age, he underwent bilateral mandibular distractor placement for mandibular distraction osteogenesis prior to cleft palate repair. He has gross motor delays and is unable to sit unsupported. He has fine motor delays and is now starting to reach for objects with one hand. He vocalizes with different vowel sounds and is receiving speech therapy to improve lingual movement and support oral motor skills.

Individual 8

Individual 8 is a 9 year old female with epilepsy, ADHD and psychiatric abnormalities. She was born via C-section at 40 weeks gestational age. Birth weight was 3260g (38%ile) and length was 48.3cm (17%ile). At time of last evaluation at 9 years of age OFC was 51.5cm (~62.5%ile), weight was 25.8kg (25%ile) and height was 129cm (26%ile). She developed epilepsy at 15 months of age, necessitating VNS placement. Several EEGs have demonstrated both generalized and focal interictal activity. Brain MRI at around 5 years old was unremarkable. She has been diagnosed with ARFID and failure to thrive, necessitating G-tube placement. She also has ADHD, anxiety, depression, and behavioral outbursts. She receives occupational therapy for fine motor difficulties. She has an IEP at school which includes special education and is currently performing at the kindergarten level despite being in second grade for the second time.

Individual 9

Individual 9 is a former 31 week gestational age male who died at 6 months of age. He was delivered via C-section due to prolonged rupture of membranes and non-reassuring fetal heart tones. At birth OFC was 30.7cm (77%ile), weight was 2720g (99%ile) and length was 44.5cm (79%ile) based on Fenton growth charts. A hearing assessment was normal. On exam he was noted to have dysconjugate gaze, hypertelorism, long eyelashes, upturned ears with fleshy lobes and mid-lobe indentation, a short, upturned nose, and wide philtrum with deep grooves. At 6 months of age, OFC was 45.5cm (89%ile), weight was 9.8kg (96%ile) and length was 61.2cm (0.51%ile) based on CDC growth charts. He had numerous congenital anomalies including severe bilateral pulmonary vein stenosis, enlarged left kidney with cortical cyst, 8th thoracic hypoplastic butterfly vertebrae, duodenal atresia, esophageal atresia with tracheoesophageal fistula, biliary atresia with absent gallbladder, and imperforate anus with rectocutaneous fistula. He ultimately underwent a Kasai procedure for biliary atresia, tracheostomy, G-tube placement and colostomy. Serial head ultrasounds demonstrated mildly prominent lateral and third ventricles and enlarged extra-axial spaces with evolving left caudothalamic subependymal hemorrhage. He subsequently was diagnosed with portal hypertension, cholestasis, necrotizing enterocolitis, ascites, and abdominal abscesses. A CMA and karyotype were normal. A cholestasis gene panel demonstrated likely pathogenic variant in *TTN* gene. He died at 6 months of age due to pulmonary hypertension secondary to inoperable bilateral pulmonary vein stenosis.

Individual 10

Individual 10 is an 18 year old male with language delays, ADHD and autism. He was born at term after induction of labor. Growth parameters at delivery included weight of 3460g (41%ile) and length of 51cm (47%ile). Audiology evaluation at 5 years of age was normal. At the time of the last evaluation at 9 years old, his weight was 27.2kg (38%ile) and height was 138.2cm (77%ile).

Individual 11

Individual 11 is a 6 year old female with epilepsy, autism and speech delay. She was born at 39 weeks gestational age by vaginal delivery. At birth, she weighed 2920g (24.1%ile). At the most recent evaluation at 5 years of age, OFC was 48.2cm (2.78%ile), weight was 17kg (35%ile) and height was 109cm (61%ile). Exam was notable for deep-set eyes, long philtrum and full cheeks. In terms of development, she was noted to have speech delay necessitating speech therapy. She was ultimately diagnosed with autism. She also displays disruptive behaviors at school and labile mood. Presently she is in a mixed classroom with mainstream and special education support. She has been diagnosed with epilepsy, with EEGs demonstrating left occipital epileptiform discharges as well as mild diffuse background slowing. Brain MRI reportedly demonstrated right hippocampal atrophy.

Individual 12

Individual 12 is a 19 year old female with global developmental delay, intellectual disability and ADHD. She was born at 37 weeks via vaginal delivery. Weight at delivery was 2140g (5%ile, Z-score -1.66). She was noted to have gross motor delay and severe language delay, did not walk until 20 months of age and did not speak until 2 years old. She was ultimately diagnosed with mild intellectual disability requiring special education and speech therapy. She also has fine motor difficulties. She was diagnosed with ADHD and noted to have several psychiatric abnormalities including anxiety, self-injurious behavior and behavioral disorder. At the time of the last evaluation at 19 years of age, OFC was 55cm (50%ile), weight was 46.1kg (5%ile, Z-score -1.62), and height was 158.9cm (8.7%ile, Z-score -1.36). Facial dysmorphisms were noted, including long face, broad forehead, epicanthus and hypertelorism with low set ears, narrow palate, bulbous nose and thick lips. Evaluation of hearing was normal, however she was noted to have strabismus. Brain MRI was normal.

Individual 13

Individual 13 is a 10 year old male with autism, ADHD, language delay, and psychiatric abnormalities. He was born at 40 weeks gestational age. Weight at time of delivery was 3345g (32%ile) and length was 49.5cm (23%ile). Exam demonstrates cryptorchidism as well as wide nasal bridge, broad nasal tip, and a long upper lip. He demonstrated delayed language acquisition for which he required speech therapy. His first words were at 2 years of age and he did not speak in sentences until around 4 years of age. He also displayed fine motor delays. He was diagnosed with autism, ADHD, anxiety, and also has auditory hallucinations. He is currently receiving special education.

Individual 14

Individual 14 is a 5 year old girl with global developmental delay and ADHD. She was born at 38 weeks gestational age following an uncomplicated delivery. Growth parameters at birth included OFC of 32cm (13%ile), weight of 2530g (12%ile) and length of 41cm (0%ile). At the time of last evaluation at 4 years of age, OFC was 42.5cm (<3%ile), weight was 9.9kg (0%ile), and height was 84.4cm (0%ile). She is currently undergoing evaluation for growth hormone therapy. Exam at that time demonstrated mildly low hanging columella, proximally placed thumb, and 5th finger clinodactyly. She has had normal cardiac evaluation, renal ultrasound, and brain MRI. She demonstrated delays in all domains and requires PT and OT. She has been diagnosed with ADHD which is being treated with stimulant medication as well as an IEP at school.

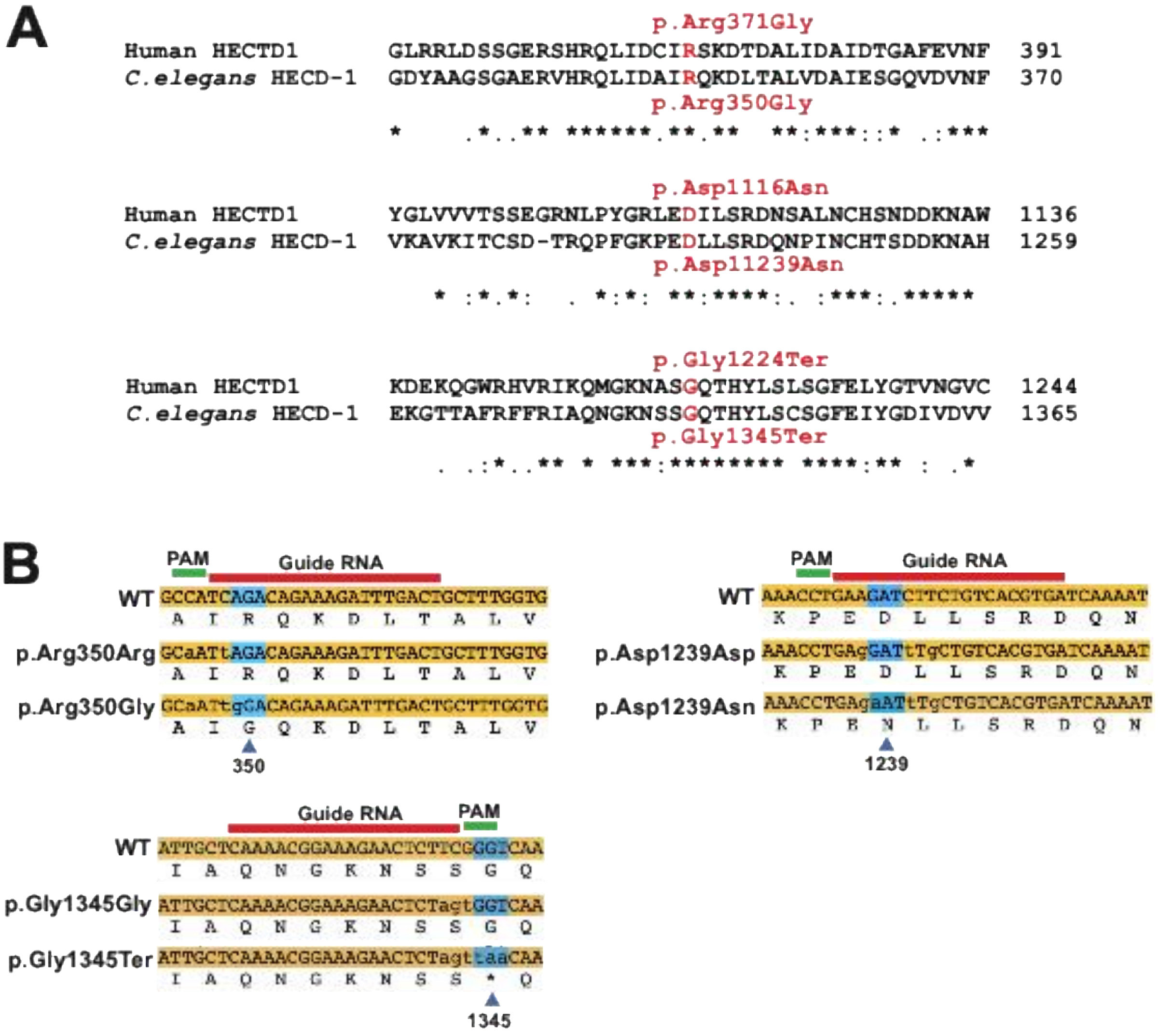

Figure S1. Comparison of human *HECTD1* and *C. elegans* HECD-1 and CRISPR/Cas9 editing details.

(A) The *C. elegans* ortholog of *HECTD1* is *hecd-1*. Human *HECTD1* variants p.Arg371Gly, p.Asp1116Asn and p.Gly1224Ter correspond to *C. elegans* HECD-1 p.Arg350Gly, p.Asp1239Asn, and p.Gly1345Ter, respectively. (B) CRISPR/Cas9 genome editing details. Variant residues are highlighted in *blue* and indicated by an *arrowhead*. Guide RNAs are indicated with a *red bar*. PAM sites are indicated with a *green bar*. Synonymous changes introduced during the CRISPR/Cas9 editing to disrupt gRNA binding following homology directed repair and facilitate allele genotyping are shown in *lower case*.

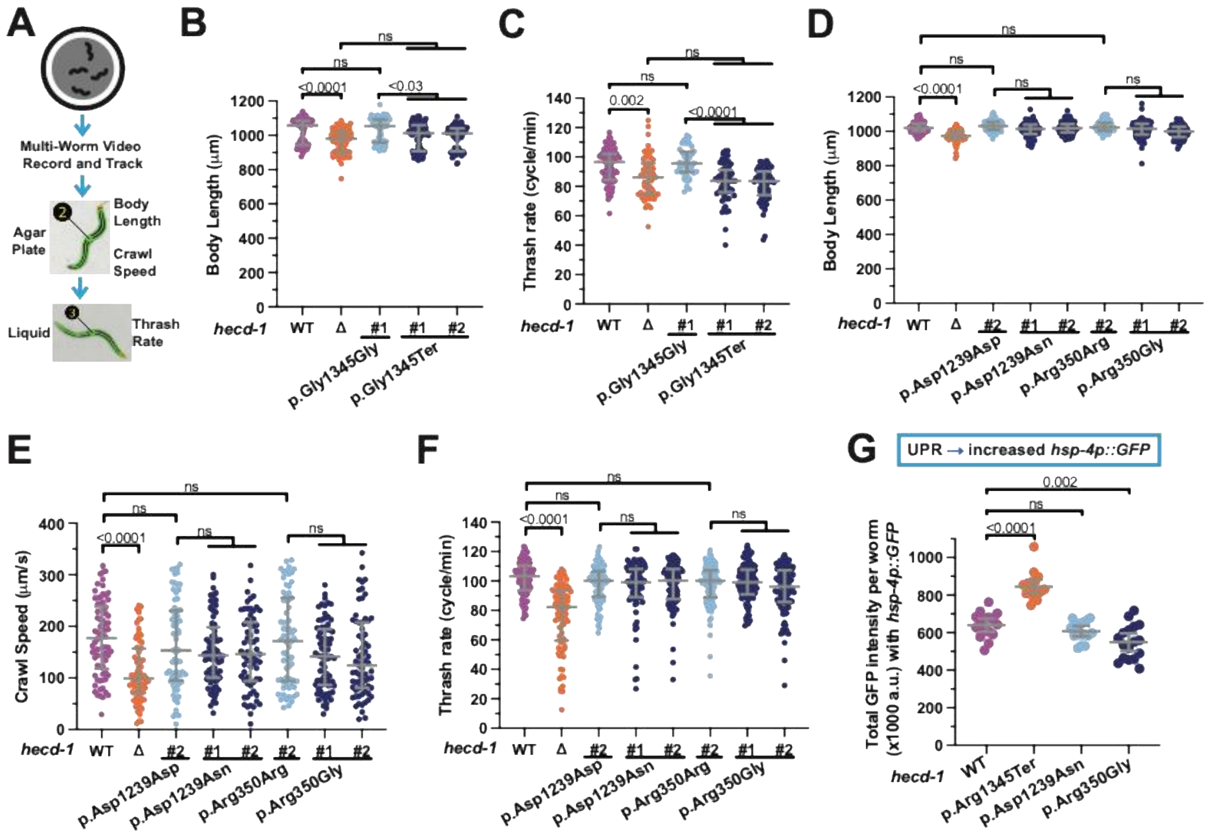

Figure S2. Phenotypic analyses of *hecd-1* variants in *C. elegans*
(A) A schematic of the WormLab workflow. One-minute videos of multiple worms crawling on an agar plate and thrashing in liquid were acquired using the WormLab (MBF Bioscience). Video analysis using the built-in software was used to calculate the body size, crawl speed and thrash (swimming) rates. (B) Body lengths and (C) thrash rates of p.Gly1345Ter variant animals. Each dot represents one animal. [n= 61-83 animals for (B). n=44-91 for (C)]. (D) Body lengths, (E) crawl speeds, and (F) thrash rates of p.Asp1239Asn and p.Arg350Gly variant animals. n=65-78 animals for (D) and (E). n=75-87 animals for (F). (G) UPR activation as measured by HSP-4::GFP expression. Each dot represents the average GFP intensity per worm in a well containing about 10 worms. n=25-27 wells for (G). In (B)-(F), error bars show median plus interquartile range. In (G), error bars show mean plus 95% confidence interval.

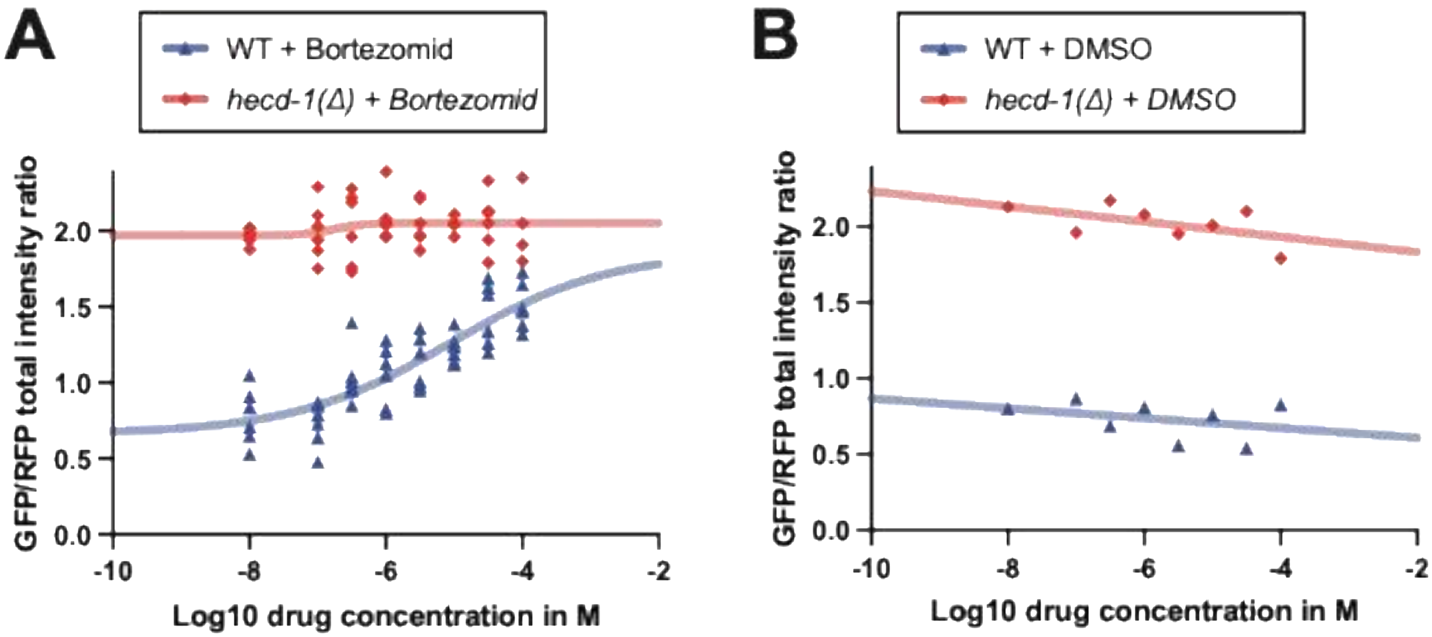

Figure S3. Bortezomib inhibition of proteasome activity in *C. elegans* as measured by Ub::GFP degradation

(A) Treatment of wild type animals expressing Ub::GFP with proteasomal inhibitor bortezomib (dissolved in DMSO) resulted in a dose-dependent increase in Ub::GFP accumulation. *hecd-1* null *(Δ)* animals have elevated Ub::GFP (see Figure 5) which was not altered by bortezomib treatment. Each dot represents a well containing about 10 animals. (n=4-6 wells) (B) Control experiment showing DMSO treatment does not change Ub::GFP levels in wild type or *hecd-1* null *(Δ)* animals. Each dot represents a well containing about 10 animal (n=1 well).

STRAIN NAME	GENOTYPE	DESCRIPTION AND REFERENCES
VC2010	wild type	WT[1]
UDN100517	*hecd-1(udn257)*	p.Arg350Gly #1 (variant, line #1)
UDN100518	*hecd-1(udn258)*	p.Arg350Gly #2 (variant, line #2)
UDN100521	*hecd-1(udn261)*	p.Arg350Arg #2 (control, line #2)
UDN100512	*hecd-1(udn252)*	p.Asp1239Asn #1 (variant, line #1)
UDN100513	*hecd-1(udn253)*	p.Asp1239Asn #2 (variant, line #2)
UDN100516	*hecd-1(udn256)*	p.Asp1239Asp #2 (control, line #2)
UDN100545	*hecd-1(udn262)*	p.Gly1345Ter #1 (variant, line #1)
UDN100540	*hecd-1(udn263)*	p.Gly1345Ter #2 (variant, line #2)
UDN100546	*hecd-1(udn265)*	p.Gly1345Gly #1 (control, line #1)
UDN100522	*hecd-1(ok1437)*	Deletion (Δ) allele. Carries a 1239bp deletion in *hecd-1* gene and results in a truncated HECD-1 protein losing its C-terminal half, from residue 1153 to the end. Outcrossed from strain RB1319.
UDN100523	*odIs77 [col−19p∷UbG76V-GFP, col−19p∷mRFP]*	Ub::GFP reporter alone
UDN100529	*hecd-1(udn257); odIs77*	p.Arg350Gly #1 with Ub::GFP reporter
UDN100530	*hecd-1(udn258); odIs77*	p.Arg350Gly #2 with Ub::GFP reporter
UDN100531	*hecd-1(udn261); odIs77*	p.Arg350Arg #2 with Ub::GFP reporter
UDN100526	*hecd-1(udn252); odIs77*	p.Asp1239Asn #1 with Ub::GFP reporter
UDN100527	*hecd-1(udn253); odIs77*	p.Asp1239Asn #2 with Ub::GFP reporter
UDN100528	*hecd-1(udn256); odIs77*	p.Asp1239Asp #2 with Ub::GFP reporter
UDN100551	*hecd-1(udn262); odIs77*	p.Gly1345Ter #1 with Ub::GFP reporter
UDN100561	*hecd-1(udn263); odIs77*	p.Gly1345Ter #2 with Ub::GFP reporter
UDN100552	*hecd-1(udn265); odIs77*	p.Gly1345Gly #1 with Ub::GFP reporter

UDN100525	*hecd-1(ok1437); odIs77*	Deletion (Δ) allele with Ub::GFP reporter
UDN100498	*zcIs4 [hsp-4p::GFP] V*	*HSP-4P::GFP* marker alone
UDN100584	*hecd-1(udn257); zcIs4*	p.Arg350Gly #1 with *hsp-4p::GFP* marker
UDN100583	*hecd-1(udn252); zcIs4*	p.Asp1239Asn #1 with *hsp-4p::GFP* marker
UDN100585	*HECD-1(UDN262); ZCIS4*	p.Gly1345Ter #1 with *hsp-4p::GFP* marker

Table S1. *C. elegans* strains
Description of *C. elegans* strains used in this study. *odls77* allele carries a multicopy insertion of the Ub::GRP reporter and mRFP under the *col-19* promoter. This reporter was used to assay protein degradation by the ubiquitin-proteosome system (UPS). *zcls4* allele carries a multicopy insertion of the plasmid, *hsp-4p*::GRP, which is activated during the unfolded protein response.

hecd-1(p.Gly1345) gRNA	CAAAACGGAAAGAACTCTTC
p.Gly1345Ter repair template	CAACCGCATTCAGATTCTTCAGAATTGCTCAAAACGG AAAGAACTCTagttaaCAAACACATTACTTGTCTTGTTCC GGATTCGAGATTTACGGAGACAT
p.Gly1345Gly repair template	CAACCGCATTCAGATTCTTCAGAATTGCTCAAAACGG AAAGAACTCTagtGGTCAAACACATTACTTGTCTTGTTC CGGATTCGAGATTTACGGAGACAT
hecd-1(p.Asp1239) gRNA	TCACGTGACAGAAGATCTTC
p.Asp1239Asn repair template	GTTAAAATCACTTGTTCGGATACTCGCCAACCATTCG GTAAACCTGAgaATtTgCTGTCACGTGATCAAAATCCTA TCAATTGTCATACTTCCGATGATA
p.Asn1239Asn repair template	GTTAAAATCACTTGTTCGGATACTCGCCAACCATTCG GTAAACCTGAgGATtTgCTGTCACGTGATCAAAATCCTA TCAATTGTCATACTTCCGATGATA
hecd-1(p.Arg350) gRNA	AGTCAAATCTTTCTGTCTGA
p.Arg350Gly repair template	TGCAGCAGGTAGTGGAGCTGAACGTGTTCATAGACA ATTGATTGACGCaATtgGACAGAAAGATTTGACTGCTTT GGTGGATGCCATCGAAAGTGGACAG
p.Arg350Arg repair template	TGCAGCAGGTAGTGGAGCTGAACGTGTTCATAGACA ATTGATTGACGCaATtAGACAGAAAGATTTGACTGCTT TGGTGGATGCCATCGAAAGTGGACAG

Table S2. CRISPR/Cas9 genome editing reagents for hecd-1
gRNA and repair template sequences used for *C. elegans* genome editing are shown. Single strand repair templates were used to introduce the variants and control edits. Lower-case letters in the repair templates denote changes compared to the wild-type sequence.

ID	Genomic location (hg38)	mRNA	Protein	Domain	Inheritance evidence	Inheritance	gnomAD MAF	Effect on protein	Splice AI	REVEL score	CADD phred	Poly phen 2
1	14:31205797_G>A	NM_015382.4:c.140C>T	NP_056197.3:p.(Thr47Ile)	ARM	NA	unknown	0			0.412	33	D
2	14:31173700_A>G	NM_015382.4:c.710T>C	NP_056197.3:p.(Leu237Ser)	ARM	PS2	*de novo*	0			0.514	27.4	D
3	14:31173201_G>C	NM_015382.4:c.1111C>G	NP_056197.3:p.(Arg371Gly)	AR	PS2	*de novo*	0			0.392	26.3	D
4	14:31150072_G>C	NM_015382.4:c.2082C>G	NP_056197.3:p.(Phe694Leu)	NA	PS2	*de novo*	0			0.379	24.6	D
5	14:31135104_C>T	NM_015382.4:c.3346G>A	NP_056197.3:p.(Asp1116Asn)	SUN	PS2	*de novo*	0			0.407	27.7	D
6	14:31135100_A>G	NM_015382.4:c.3350T>C	NP_056197.3:p.(Ile1117Thr)	SUN	PS2	*de novo*	0			0.555	27.1	D
7	14:31133545_A>T	NM_015382.4:c.3709T>A	NP_056197.3:p.(Tyr1237Asn)	SUN	PS2	*de novo*	0			0.68	29.5	D
8	14:31129377_G>A	NM_015382.4:c.3994C>T	NP_056197.3:p.(Leu1332Phe)	MIB	PS2	*de novo*	0			0.624	28	D
9	14:31106737_G>A	NM_015382.4:c.7135C>T	NP_056197.3:p.(Leu2379Phe)	HECT	PS2	*de novo*	0			0.203	24.5	D
10	14:31169471_AGAG>A	NM_015382.4:c.1333-6_1333-4del	splice variant	NA	PS2	*de novo*	0	BP4	0.01	NA	NA	NA
11	14:31148966_C>CT	NM_015382.4:c.2349dup	NP_056197.3:p.(Asp784ArgfsTer8)	NA	NA	unknown	0	PVS1		NA	NA	NA
12	14:31133584_C>A	NM_015382.4:c.3670G>T	NP_056197.3:p.(Gly1224Ter)	SUN	PM6	*de novo*	0	PVS1		NA	38	NA
13	14:31103032_T>TA	NM_015382.4:c.7433dup	NP_056197.3:p.(Leu2478PhefsTer7)	HECT	NA	unknown	0	PVS1		NA	NA	NA
14	14:31107648_AG>A	NM_015382.4:c.6536delC	NP_056197.3:p.(Ala2179ValfsTer35)	HECT	PM3	compound het	0	PVS1		NA	NA	NA
14	14:31175038_T>C	NM_015382.4:c.476A>G	NP_056197.3:p.(His159Arg)	ARM	PM3	compound het	0			0.735	26.1	P

Table S3: *HECTD1* Variant information and functional prediction scores

The variant classifications in "Inherit evidence" and "Effect on protein" columns are based on the ACMG criteria[2]. Truncating variants are classified as PVS1 while the splice variant with benign predicted Splie AI score is classified as BP4. Splice AI[3] , CADD phred[4], REVEL[5] and Polyphen2[6] are prediction programs for pathogenicity of variants. Domain abbreviations: SUN=Sad1 and UNC-84; ARM=armadillo-like helical; AR=ankyrin repeat; HECT=homologous to E6AP C-terminus; MIB= MIB/HERC2. MAF=minor allele frequency from gnomAD, het=heterozygous, D=damaging, P=possibly damaging, NA=Not applicable.

PMID	Phenotype
35654974	Autism
30675382	Autism
37543562	Autism
35982159	Autism
27525107	Autism
28263302	Autism
26582266	Autism
23260136	Autism
25363760	Autism
26749308	Autism
24650168	Autism
24501278	Autism
25418537	Autism
26352270	Autism
28965761	Autism
25961944	Autism
23160955	Autism
30504930	Autism
30545852	Autism
26785492	Congenital Heart Disease
27479907	Congenital Heart Disease
23665959	Congenital Heart Disease
33057194	Developmental Disorder
23934111	Epilepsy
23647072	Epilepsy
26795593	Epilepsy
22365152	Epilepsy

23086397	Epilepsy
27479843	Intellectual Disability
23033978	Intellectual Disability
27334371	Intellectual Disability
37158973	Neurodevelopmental Disorder
38114583	Neurodevelopmental Disorder

Table S4. Source and phenotype of published cohort data used in DenovolyzeR and chimpanzee-human *de novo* variant (DNV) analysis

Phenotype	Unique Number of Individuals
Autism	19,391
Congenital Heart Disease	2,072
Developmental Disorder	31,058
Epilepsy	532
Intellectual Disability	190
Neurodevelopmental Disorder	62
Total	53,305

Table S5. Phenotypes and counts of unique individuals in published cohorts after removing overlaps between studies

Chrom	Pos (b38)	Ref	Alt	Sample	PMID	Phenotype	Consequence	HGVSc	HGVSp
chr14	31106802	G	C	1-02563	26785492	congenital_heart_disease	missense	NM_015382.4:c.7070C>G	NP_056197.3:p.(Ala2357Gly)
chr14	31107685	T	C	DDD13k.02131	33057194	developmentalDisorder	missense	NM_015382.4:c.6500A>G	NP_056197.3:p.(Glu2167Gly)
chr14	31109507	G	A	DDD13k.04533	33057194	developmentalDisorder	stop_gained	NM_015382.4:c.6370C>T	NP_056197.3:p.(Arg2124Ter)
chr14	31112423	C	T	rumc_patient_476	33057194	developmentalDisorder	missense	NM_015382.4:c.6271G>A	NP_056197.3:p.(Ala2091Thr)
chr14	31113390	G	A	3456	33057194	developmentalDisorder	missense	NM_015382.4:c.5951C>T	NP_056197.3:p.(Pro1984Leu)
chr14	31121394	T	C	SP0130244	35982159	autism	missense	NM_015382.4:c.5227A>G	NP_056197.3:p.(Ile1743Val)
chr14	31128819	C	A	SP0123550	35982159	autism	missense	NM_015382.4:c.4552G>T	NP_056197.3:p.(Val1518Leu)
chr14	31133321	A	C	27672	33057194	developmentalDisorder	missense	NM_015382.4:c.3839T>G	NP_056197.3:p.(Val1280Gly)
chr14	31136577	T	C	71821	33057194	developmentalDisorder	missense	NM_015382.4:c.3068A>G	NP_056197.3:p.(Glu1023Gly)
chr14	31144125	G	GT	13711.p1	25363768	autism	frameshift	NM_015382.4:c.2764dup	NP_056197.3:p.(Thr922AsnfsTer6)
chr14	31144245	C	G	rumc_patient_872	33057194	developmentalDisorder	missense	NM_015382.4:c.2644G>C	NP_056197.3:p.(Glu882Gln)
chr14	31173239	G	A	5352	33057194	developmentalDisorder	missense	NM_015382.4:c.1073C>T	NP_056197.3:p.(Ser358Phe)
chr14	31173538	G	A	117451	33057194	developmentalDisorder	missense	NM_015382.4:c.872C>T	NP_056197.3:p.(Pro291Leu)
chr14	31173700	A	G	53609	33057194	developmentalDisorder	missense	NM_015382.4:c.710T>C	NP_056197.3:p.(Leu237Ser)

Table S6. Missense and likely gene disrupting *HECTD1 de novo* variants in published cohorts

Gene	Class	Observed	Expected	Enrichment	P-value
HECTD1	mis	12	7.5	1.6	0.0785
HECTD1	lof	2	1	2.03	0.259
HECTD1	prot	14	8.5	1.65	0.0504

Table S7. Enrichment of *HECTD1 de novo* variants in published cohorts derived from DenovolyzeR analysis

GeneName	Nonsynon	LoF	Allp	Nsp	Truncp
HECTD1	12	2	4.02E-06	2.47E-05	0.05218636

Table S8. Enrichment of *HECTD1 de novo* variants in published cohorts derived from Chimpanzee-Human De Novo Variant (DNV) test

REFERENCES

1. Yoshimura, J., Ichikawa, K., Shoura, M.J., Artiles, K.L., Gabdank, I., Wahba, L., Smith, C.L., Edgley, M.L., Rougvie, A.E., Fire, A.Z., et al. (2019). Recompleting the Caenorhabditis elegans genome. Genome Res *29*, 1009-1022. 10.1101/gr.244830.118.
2. Richards, S., Aziz, N., Bale, S., Bick, D., Das, S., Gastier-Foster, J., Grody, W.W., Hegde, M., Lyon, E., Spector, E., et al. (2015). Standards and guidelines for the interpretation of sequence variants: a joint consensus recommendation of the American College of Medical Genetics and Genomics and the Association for Molecular Pathology. Genet Med *17*, 405-424. 10.1038/gim.2015.30.
3. Jaganathan, K., Kyriazopoulou Panagiotopoulou, S., McRae, J.F., Darbandi, S.F., Knowles, D., Li, Y.I., Kosmicki, J.A., Arbelaez, J., Cui, W., Schwartz, G.B., et al. (2019). Predicting Splicing from Primary Sequence with Deep Learning. Cell *176*, 535-548 e524. 10.1016/j.cell.2018.12.015.
4. Kircher, M., Witten, D.M., Jain, P., O'Roak, B.J., Cooper, G.M., and Shendure, J. (2014). A general framework for estimating the relative pathogenicity of human genetic variants. Nat Genet *46*, 310-315. 10.1038/ng.2892.
5. Ioannidis, N.M., Rothstein, J.H., Pejaver, V., Middha, S., McDonnell, S.K., Baheti, S., Musolf, A., Li, Q., Holzinger, E., Karyadi, D., et al. (2016). REVEL: An Ensemble Method for Predicting the Pathogenicity of Rare Missense Variants. Am J Hum Genet *99*, 877-885. 10.1016/j.ajhg.2016.08.016.
6. Adzhubei, I.A., Schmidt, S., Peshkin, L., Ramensky, V.E., Gerasimova, A., Bork, P., Kondrashov, A.S., and Sunyaev, S.R. (2010). A method and server for predicting damaging missense mutations. Nat Methods *7*, 248-249. 10.1038/nmeth0410-248.
